# Integrative Analysis of Transcriptional Regulatory Network and Copy Number Variation in Intrahepatic Cholangiocarcinoma

**DOI:** 10.1371/journal.pone.0098653

**Published:** 2014-06-04

**Authors:** Ling Li, Baofeng Lian, Chao Li, Wei Li, Jing Li, Yuannv Zhang, Xianghuo He, Yixue Li, Lu Xie

**Affiliations:** 1 School of Life Sciences and Technology, Tongji University, Shanghai, P.R.China; 2 Shanghai Center for Bioinformation Technology, Shanghai Academy of Science and Technology, Shanghai, P.R.China; 3 School of Life Sciences and Technology, Shanghai Jiaotong University, Shanghai, P.R.China; 4 Key Lab of Systems Biology, Shanghai Institutes for Biological Sciences, Chinese Academy of Sciences, Shanghai, P.R.China; Queen's University Belfast, United Kingdom

## Abstract

**Background:**

Transcriptional regulatory network (TRN) is used to study conditional regulatory relationships between transcriptional factors and genes. However few studies have tried to integrate genomic variation information such as copy number variation (CNV) with TRN to find causal disturbances in a network. Intrahepatic cholangiocarcinoma (ICC) is the second most common hepatic carcinoma with high malignancy and poor prognosis. Research about ICC is relatively limited comparing to hepatocellular carcinoma, and there are no approved gene therapeutic targets yet.

**Method:**

We first constructed TRN of ICC (ICC-TRN) using forward-and-reverse combined engineering method, and then integrated copy number variation information with ICC-TRN to select CNV-related modules and constructed CNV-ICC-TRN. We also integrated CNV-ICC-TRN with KEGG signaling pathways to investigate how CNV genes disturb signaling pathways. At last, unsupervised clustering method was applied to classify samples into distinct classes.

**Result:**

We obtained CNV-ICC-TRN containing 33 modules which were enriched in ICC-related signaling pathways. Integrated analysis of the regulatory network and signaling pathways illustrated that CNV might interrupt signaling through locating on either genomic sites of nodes or regulators of nodes in a signaling pathway. In the end, expression profiles of nodes in CNV-ICC-TRN were used to cluster the ICC patients into two robust groups with distinct biological function features.

**Conclusion:**

Our work represents a primary effort to construct TRN in ICC, also a primary effort to try to identify key transcriptional modules based on their involvement of genetic variations shown by gene copy number variations (CNV). This kind of approach may bring the traditional studies of TRN based only on expression data one step further to genetic disturbance. Such kind of approach can easily be extended to other disease samples with appropriate data.

## Introduction

Transcriptional regulatory network (TRN) is a directed graph describing regulatory effect of transcriptional factors (TFs) on genes' expression by binding to target DNA. Over last decades, several methods of studying regulatory relationship between TFs and genes under a given set of conditions have been proposed and widely used, like ChIP-chip, genome-wide RNA interference and DNase I footprinting assay [1], [2]. Most of these technologies based on the molecular biology or biochemistry are experimental techniques with limitation on mass samples. Therefore, computational biologists have resorted to a forward engineering strategy which is based on searching of transcriptional factor binding sites in the putative target sequences [3]. To reduce the false positive rates of forward engineering method, Yu et al proposed a combinatorial inferring method that integrates forward engineering with reverse engineering of which relationships between TFs and targets are inferred based on expressional correlation [4].

Compared with other networks, TRN has advantages in properties of reflecting regulatory relationship, dynamics and scale-free topological structure. TRN depicts the transcriptional regulation of TFs on target genes which is an important regulatory mechanism of gene expression. Neph S et al studied TRN of 41 diverse cell and tissue types using DNase I footprinting technology and found that human TF networks are highly cell selective [5]. TRN is a scale-free network, in which the number of nodes that make a large number of connections with other nodes (referred to as “hubs”) is much lower than the number of nodes with few connections, whereby hubs play a central role in directing the cellular response to a specific stimulus [1]. All these features make TRN an irreplaceable tool in disease research. In 2012, Zeng et al found hepatocellular carcinoma metastasis related TF-regulated modules by comparing regulatory network between metastatic and non-metastatic liver cancer [6].

With the development of high-throughput technology, especially the flourish of SNP microarray, combined analysis of genome and transcriptome is becoming increasingly popular, and has greatly promoted our understanding of complex diseases. Copy number variation (CNV), an important kind of genomic variation, has gained increasing attention in recent years mainly due to SNP microarray technology which has made studying whole genome fast and economical. The importance of CNVs to occurrence and development of disease has been confirmed in many studies [7].

Until now, most studies of CNVs are focused on CNVs' impact on expression of genes located in verified regions, like eQTL [8], a linear-regression based method. Others may combine CNV with network method, like co-expression network [9] to analyze CNVs' impact on not just genes inside CNV regions but also outside CNV regions that are co-expressed.

But there is little work about interpreting influence of genomic variation on expression through its disturbance to TRN. Mutation in TFs can cause huge cascade effects as a TF targets a large amount of genes involving many biological processes [10]. For example, TP53, a well-known tumor suppressor transcription factor, its mutation has been reported associated with cell migration and invasion [11], [12]. In 2012, David et al detailed three mutated transcriptional factors NKX2-5, GATA4, and TBX5 and their affected pathways in congenital heart disease [13]. Essaghir et al introduced an integrated approach to construct minimal connected network to TFs in 305 different human cancer cell lines and found several universal cancer biomarkers [14]. These researches suggest the importance and feasibility of integrating TRN with CNVs.

Intrahepatic cholangiocarcinoma(ICC) is the second most common primary hepatic cancer with the highest occurring rate in Thailand and other eastern Asian areas due to chronic inflammation of bile ducts [15]. In 2013, Sia et al performed gene expression and copy number variation integrated analysis in ICC samples and classified these samples into two groups: proliferation and inflammation [16].

**Figure 1:**
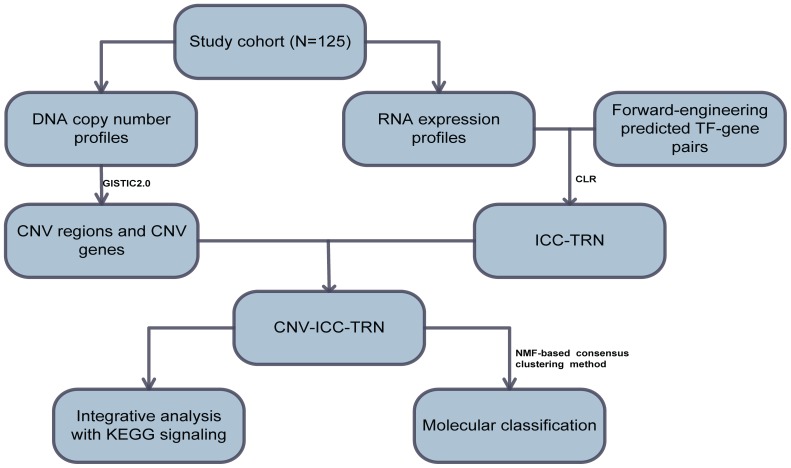
Workflow of integrative analysis of TRN and CNV in ICC.

In this research, we analyzed Sia et al data in a new perspective. We constructed CNV genes related TRN of ICC (CNV-ICC-TRN), integrated it with signaling pathways to see how CNV genes disturb signaling transduction, and used it to classify ICC samples into two molecular subtypes with distinct functional features. The work flow is shown in Figure 1.

## Methods

### Materials and preprocess

Paired gene-expression profiles and DNA copy number profiles of 125 ICC samples were downloaded from NCBI Gene Expression Omnibus (GEO, http://www.ncbi.nlm.nih.gov/geo/), the accession number is GSE33327 [16]. The platforms of these two kinds of datum are Illumina HumanCytoSnp-12 Beadchip version 1.0 and Illumina HumanRef-8 WG-DASL v3.0 respectively.

We downloaded probe-level expression profiles which were normalized by cubic spline algorithm, a non-linear normalization method using array signal distribution analysis and cubic splines [17], calculated gene-level expression profiles using R package dplR [18].Then we filtered out genes within the 5% smallest standard deviations among all samples, 17428 genes were retained for further analysis.

For DNA copy number profile, we ran segmentation analysis using Circular Binary Segmentation algorithm (CBS) [19]. Regions with amplifications or deletions were identified using GISTIC2.0 (GISTIC2.0 module, GenePattern http://www.broadinstitute.org/cancer/software/genepattern [20]). Copy number analysis was based on Human Genome Hg18. Thresholds used for defining copy number amplification and deletion are 0.3 and -0.3 respectively. Regions with q-value less than 0.25 were considered significant. Other parameters were default.

### CNV-ICC-TRN construction

Forward engineering prediction of TF-gene regulatory relationship was based on the sequence complementarity between regulators and their targets. Reverse engineering method uses expression datasets to filter condition-specific sub-network from reference network, of which connection between nodes was based on expressional correlation.

Firstly, we used forward-and-reverse combined engineering (File S1) method to construct ICC-specific TRN (ICC-TRN). Sequence-based TF-gene pairs were downloaded from our web platform for building combinatorial Gene Regulation Networks (cGRNB, http://www.scbit.org/cgrnb/), which defines TF-gene pair as that TF's binding site should be located between upstream 1 kb and downstream 0.5 kb of transcription start sites [21]. 203633 TF-gene pairs were obtained with expression profiles.

Secondly, the Context Likelihood of Relatedness (CLR), a mutual information based network inference method from R/Bioconductor package minet [22], was applied to compute expression correlation of these pairs. CLR computes the mutual information (MI) for each pair of genes and derives a score related to the empirical distribution of the MI values. Formally, the MI for two genes X and Y is defined as:
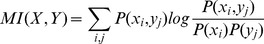
Where *xi, yj* represent particular expression levels of *X* and *Y*, *P*(*xi*) and *P*(*yj*) are the probabilities that *X = xi* and *Y* = *yj*, and *P(xi, yj)* is the joint distribution of *X* and *Y* (More description is provided in File S1). CLR returns an adjacency matrix, values of which represent edge weight. For each module, we supposed that correlation between a sequence-based TF-gene pair was stronger than a random one, so we selected significantly correlated pairs with CLR values larger than 95% of 1000 randomly selected non-sequence-based pairs. After this screening, 9196 pairs were retained including 164 modules and 4898 genes.

Finally, we performed filtration to extract CNV related ICC-TRN (CNV-ICC-TRN). We set two criteria that modules left should have clear biological function, and they must be regulated by CNV-TFs or enriched by CNV-genes. We therefore performed two kinds of enrichment analysis using Fisher's exact test: one is based on KEGG signaling pathway, another is based on CNV-genes. For the first kind of test, base set was all 17428 genes with expression; while for the second one, the base set was 4898 genes in ICC-TRN. And the significance threshold was FDR<0.05.

### Integrative analysis of CNV-ICC-TRN and KEGG signaling pathway

We overlapped nodes containing CNV or regulated by CNV-TF in CNV-ICC-TRN with KEGG signaling pathways, and neighbors of overlapped nodes in signaling pathways were embodied using R package KEGGgraph [23]. The integrative network was constructed by combining edges of CNV-ICC-TRN and edges of signaling pathways, both of which were connected with overlapped nodes, shown through Cytoscape [24].

### Unsupervised clustering and leave-one-out cross validation

Expression profiles of genes in CNV-ICC-TRN were used to perform unsupervised clustering using nonnegative matrix factorization (NMF)-based consensus clustering method (NMFconsensus module, GenePattern). NMF decomposes a nonnegative matrix *V* into two nonnegative matrices *W* and *H*, *V∼WH*. In the context of *p×n* expression matrix *V* consisting *p* genes' expression profiles in *n* samples, *W* is a *p×k* metagenes matrix of which each column represents a metagene, and *H* is a *k×n* expression matrix of which each row is expression pattern of a metagene in *n* samples. The rank *k* of the factorization represents the number of latent factors in the decomposition (in our case, *k* is the number of clusters) [25].

Then three leave-one-out cross-validation (LOOCV) based modules from GenePattern were used to evaluate the robustness of the clustering result: KNNXValidation, WeightedVotingXValidation, and CARTXValidation. In each round of cross-validation, LOOCV takes a single observation from the original sample as the validation data, and the remaining observations as the training data (More details about NMF and LOOCV can be found in File S1). All these analysis were performed using GenePattern, and parameters were default. T-test was used to select differentially expressed genes between two subgroups (significance threshold p-value<0.001).

## Result

### Chromosome aberration: CNV

We first studied DNA copy number profiles of 125 ICC samples, and found 42 regions with genomic variation including 12 amplified regions and 30 deleted regions. These CNV regions covered 4221 genes among which 39 were TFs (CNV-TF) and others were non-TF genes (CNV-gene). CNV regions containing TFs are shown in Table 1 and all variation information are shown in Table S1. Among these TF-containing regions, losses of 3p [26] and 9p [26], [27], have been reported with more than 20% prevalence in chromosome aberration studies of ICC. Most TFs are located in loss regions except RUNX1 who is the only TF located in gain region. An interesting region 19p13.2, even though mutated at low frequency, covered 15 TFs indicating its potential role in ICC development as some studies have figured out the importance of low frequency CNVs to cancer risk [28], [29].

**Table 1:** CNV-regions containing TFs.

Cytoband	q.value	Amp/Del	Frequence	TFs
**3p26.2**	1.69E-02	Del	0.16	PPARG
**6q21**	7.38E-06	Del	0.168	ESR1,FOXO3,HSF2,MYB,POU3F2,TBP,DACH2
**8p23.1**	2.87E-02	Del	0.104	EGR3,NKX3-1
**10q26.13**	4.56E-02	Del	0.04	EGR2,NFKB3,DAX2,PLAU,HMX3
**12q24.33**	7.84E-02	Del	0.048	NFYB,HNF1A,ALX1
**13q12.11**	2.61E-01	Del	0.144	FOXO1,ZIC2,KLF12
**19p13.2**	1.48E-01	Del	0.016	BAX,CEBPA,FOSB,JUND,JUNB,POU2F2,PSG1,RFX1,TCF3,USF2,M2F1,NFIC,RNASEH2A,DAND5,ZSCAN1
**16q24.3**	0.10881	Del	0.08	FOXL1
**14q32.32**	2.82E-03	Del	0.176	YY1
**21q22.12**	6.09E-03	Amp	0.04	RUNX1

Node: Amp is short for amplification, and Del is for Deletion. All chromosomal aberration regions are listed at Table S3.

### CNV -ICC-TRN

#### Network structure of CNV-ICC-TRN

**Figure 2:**
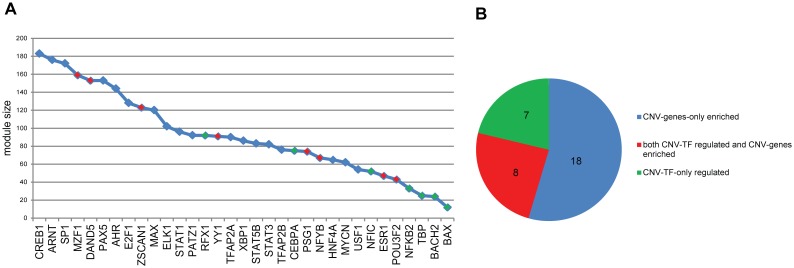
Overview of module subtype and size in CNV-ICC-TRN. In both A and B figures, blue color represents CNV-gene-only enriched module, green color represents CNV-TF-only regulated module, red color represents both CNV-TF regulated and CNV-gene enriched module.

We first constructed TRN of ICC (ICC-TRN) using forward-and-reverse combined engineering method, and then extracted modules from ICC-TRN to form CNV-ICC-TRN according to the following selection standards: 1) the selected modules should have biological significance; 2) the selected modules are regulated by CNV-TF, or enriched by CNV-genes. With such standards, we finally obtained a CNV-ICC-TRN containing 33 regulatory modules, each of them composed by one specific TF and all of its first-layer targets (Details of CNV-ICC-TRN shown at Table S2). The size and type of every regulatory module are shown in Figure 2. There were three types of modules according module selecting criteria: CNV-TF-only regulated modules, CNV-genes-only enriched modules, and both CNV-TF regulated and CNV-genes enriched modules. We could see that the top three largest modules are CNV-gene-only enriched modules, and CNV-TF-only regulated modules have relatively smaller sizes. Eight modules are both CNV-TF regulated and CNV-gene enriched, and among these eight CNV-TFs, YY1 [30], MZF1 [31], DAND5 [32], NFYB [33], ESR1 [34] have been reported in liver cancer development and metastasis.

CNV-ICC-TRN was composed of four kinds of nodes according whether they were inside or outside CNV regions: CNV-TF, CNV-gene, non-CNV-TF and non-CNV-gene, and seven kinds of edges between these nodes: CNV-TF to CNV-TF, CNV-TF to non-CNV-TF, non-CNV-TF to non-CNV-TF, CNV-TF to CNV-gene, CNV-TF to non-CNV-gene, non-CNV-TF to CNV-genes and non-CNV-TF to non-CNV-gene. The statistics about nodes and edges are shown in Table 2. Nodes inside CNV regions were about a quarter of all nodes, and edges connected with these nodes accounted forty-seven percent of total edges, so we might conclude that ICC-CNV-TRN is highly CNV-genes intensive.

**Table 2:** Overall statistics about the nodes and edges of CNV-ICC-TRN.

Category	Sub Category	Types	Num.
**Nodes**	TF	CNV-TF	24
		non-CNV-TF	54
	gene	CNV-gene	408
		non-CNV-gene	1898
**Edges**	TF-TF	CNV-TF to CNV-TF	5
		CNV-TF to non-CNV-TF	20
		non-CNV-TF to non-CNV-TF	36
	TF-gene	CNV-TF to CNV-gene	177
		CNV-TF to non-CNV-gene	868
		non-CNV-TF to CNV-gene	350
		non-CNV-TF to non-CNV-gene	1567

#### Biological functions tackled in modules of CNV-ICC-TRN

**Figure 3:**
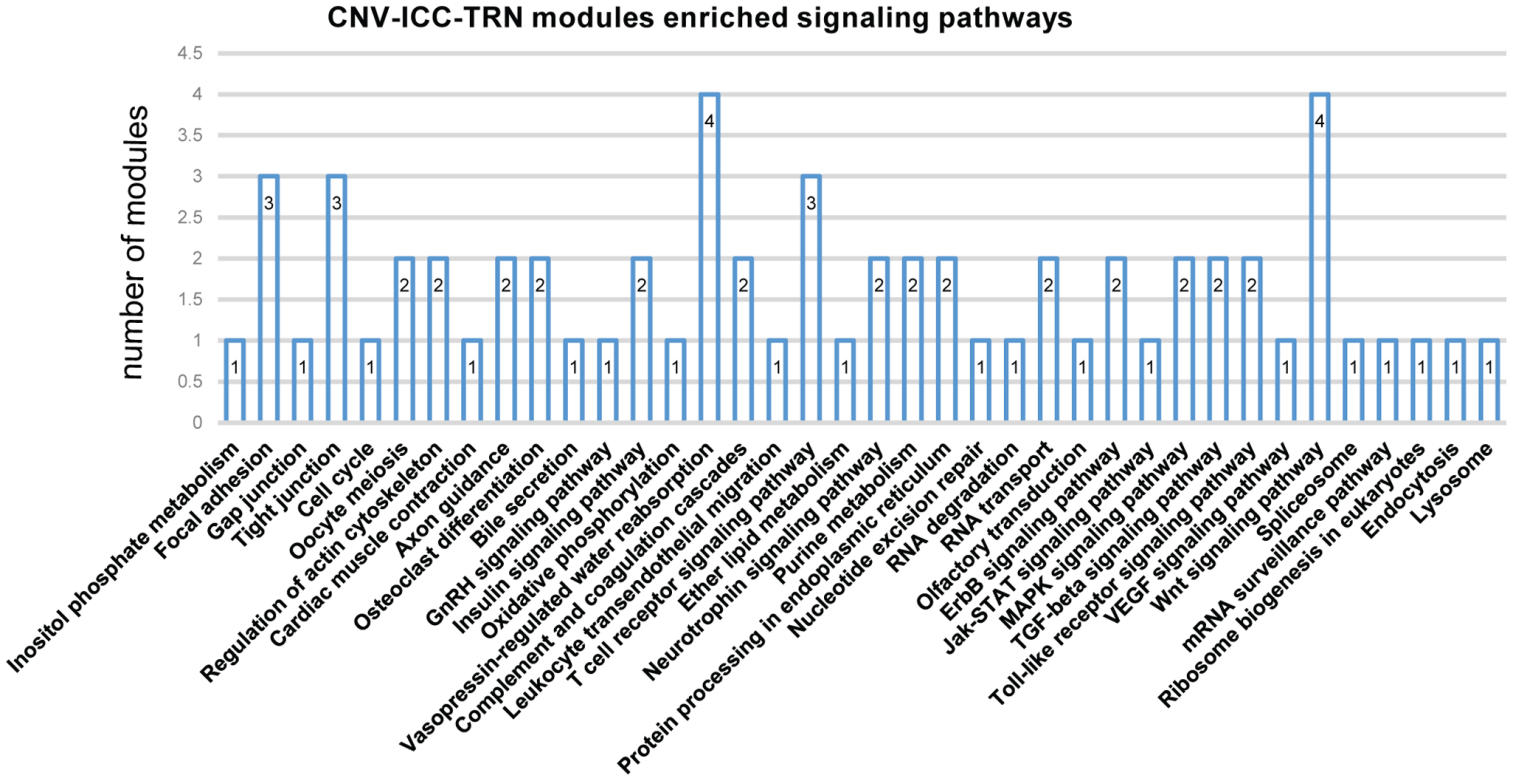
Biological functions tackled in modules of CNV-ICC-TRN. X-axis represents signaling pathways and IDs, y-axis represents the number of modules enriching to each pathway. Complete information is shown in Table S3.

To study biological functions of modules of CNV-ICC-TRN involved in, we implemented enrichment analysis based on KEGG signaling pathways, results are shown in Figure 3 and . These modules were extensively enriched into five categories: signal transduction, cell communication, immune system, metabolism, and disease and cancer related pathways, which coincide with ICC's clinical and pathological features of high degree malignancy, poor prognosis and inflammation [35]. Among all involved pathways, Wnt signaling pathway was enriched by four modules of CNV-ICC-TRN, regulated by AHR, TFAP2A, NFKB2 and PAX5. Wnt signaling activation was associated with low differentiation and high proliferation in human biliary tract cancer [36]. Vasopressin-regulated water reabsorption was also enriched by four modules. There has been no research relating this pathway to any cancers, but water balance is very important to homeostasis, disorder of which could break homeostasis that may ultimately contribute to cancer [37]. MAPK signaling pathway was enriched by NFKB and PSG1 modules of CNV-ICC-TRN. MAPK signaling has been reported involved in biliary epithelial cell growth [38]. Similarly, JAK/STAT signaling pathway enriched by TFAP2A was reported to be activated in 50% of ICC, and might affect more than 70% of the ICC inflammation subclass [15]. Moreover, ERBB2 signaling pathway enriched by SP1 and ELK1 has been implicated in the molecular pathogenesis of intrahepatic cholangiocarcinoma by interacting with other relevant signaling pathways, including linking to bile acid, vascular endothelial growth factor signaling [39].

On the other hand, some modules were very active as they were enriched to several signaling pathways, such as CNV-genes-only enriched modules: AHR, E2F1, PTAZ1, SP1, CNV-TF-only regulated modules: NFKB2, and both CNV-TF regulated and CNV-gene enriched modules: YY1, DAND5 and MZF1.

### Disturbance of genes in CNV regions to signaling pathways

**Figure 4:**
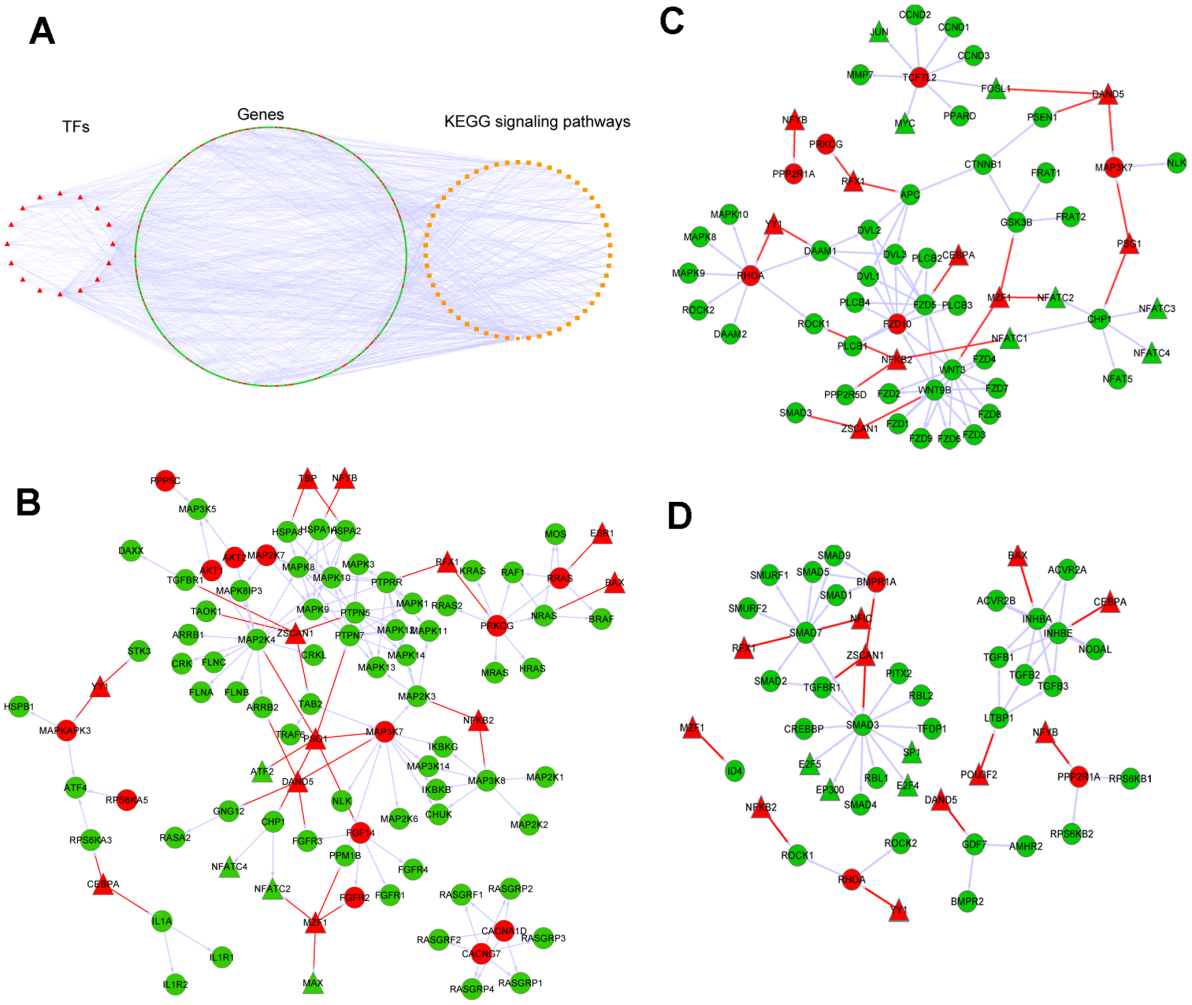
Integrative analysis of CNV-ICC-TRN and KEGG signaling pathways. (A). Integrated network of CNV-ICC-TRN and KEGG signaling pathways. Triangle represents TF, circle represents gene and rectangle represents signaling pathway; red color means gene inside CNV region, green color means gene outside CNV region. (B) Integrative analysis in MAPK signaling pathway. (C) Integrative analysis in Wnt signaling pathway. (D) Integrative analysis in TGF-β signaling pathway. In figure B, C, D, red edges are from CNV-ICC-TRN, and off-white edges are from signaling pathways.

Modules' biological function analysis showed that these modules were enriched to some ICC related signaling pathways. So we performed integrative analysis of CNV-ICC-TRN and KEGG signaling pathways to study how CNV-genes in network disrupt signaling pathways. Results shown in Figure 4A, reflected that some genes of signaling pathways had copy number variation, but most were only regulated by CNV-TFs. From this we might conclude that genomic variations could affect signaling pathways in two ways: at some cases, variation happens on genes of signaling pathways; at most cases, variation happens on regulators such as TFs that can lead to abnormal expression of genes in signaling pathways. We also found that CNV-TFs YY1, ZSCAN1, MZF1 and DAND5 regulated a large number of genes involved in a variety of signaling pathways; and some non-disease signaling pathways such as Wnt signaling pathway, MAPK signaling pathway and TFG-beta signaling pathway had more than thirteen percent of genes regulated by CNV-TFs or located in genomic variation regions. This indicates that genomic variation of ICC in these TFs regions can cause dysfunction of a variety of pathways, and some pathways may be fundamentally deregulated.

Then we investigated three pathways in detail which are closely related to the development of ICC: MAPK signaling pathway, Wnt signaling pathway and TGF-β signaling pathway.

### Integrative analysis of CNV-ICC-TRN and MAPK signaling pathway

The MAPK cascade is a highly conserved module which participates in various cellular functions, including cell proliferation, differentiation and migration, and it might be a personalized therapy target in ICC [40]. Result of CNV-ICC-TRN and MAPK signaling integration is shown in Figure 4B. CNV-genes MAP3K7 and MAP2K7 are two mitogen-activated protein kinase kinases, playing important roles in cell response to environmental stresses and inflammation [41], [42]. CNV-TF NFKB2 regulated two MAPK upstream kinases MAP2K3 and MAP3K8. CNV-TFs TBP and NFYB targeted HSPA2, HSPA8 and HSPA1A respectively, three members of heat shock protein 70 which could inhibit apoptosis in cancer cells through simultaneous and independent mechanisms [43]. CNV-genes AKT1 and AKT2 could interplay with MAPK signaling pathway in regulating cell apoptosis [44], and study has shown that there is a fine balance of cross-talk between mitogenic RAS/MAPK and survival PI3K/AKT pathways [45]. CNV-genes CACNA1 and CACNG7, two calcium channel subunits, compound with RAS guanyl nucleotide releasing proteins which are guanyl nucleotide exchange factors that activate Ras and related GTPases such as RAP [46]. CNV-genes FGFR2 could induce cholangiocarcinoma cell migration via activation of the MEK1/2 pathway [47]. CNV-TF PSG1 regulated upstream activator PTPN7, downstream target ATF2 [48] of MAPK pathway.

### Integrative analysis of CNV-ICC-TRN and Wnt signaling pathway

Wnt signaling transmitting signals from outside through cell surface receptors to the inside of the cell, is required for cell differentiation and proliferation, and inhibition of which can induce cell apoptosis and suppress cell proliferation in cholangiocarcinoma cells [49]. Result of CNV-ICC-TRN and MAPK signaling integration is shown in Figure 4C. CNV-gene FZD10, a cell-surface receptor for Wnt proteins, was reported negatively related with Wnt signal transduction in colorectal cancer [50]. CNV-gene RHOA, downstream target of Wnt signaling, is a member of Rho family of small GTPases which were promising cellular targets for novel anticancer drugs [51]. CNV-gene TCF7L2 played a key role in Wnt signaling and was associated with susceptibility of hepatocellular carcinoma [52]. Two core members of Wnt family WNT9B and WNT3 were targeted by two CNV-TFs MZF1 and ZSCAN1 respectively.

### Integrative analysis of CNV-ICC-TRN and TGF-β signaling pathway

TGF-β signaling takes part in in many cellular processes such as proliferation, apoptosis, differentiation and migration by activating SMAD signaling. Munker et al reported that TGF-β1 could contribute to ICC via SMAD dependent and independent pathway [53]. Result of CNV-ICC-TRN and TGF-β signaling integration is shown in Figure 4D. Three CNV-TFs ZSCAN1, RFX1 and NFIC targeted two SMAD proteins SMAD3 and SMAD7. Study of Huang et al showed that SMAD7 was highly expressed in cholangiocarcinoma and might be a potential prognostic indicator for clinical assessment [54]. ZSCAN1 also targeted TGFBR1 which can activate SMAD proteins.

### Clustering analysis reveals two ICC classes

**Figure 5:**
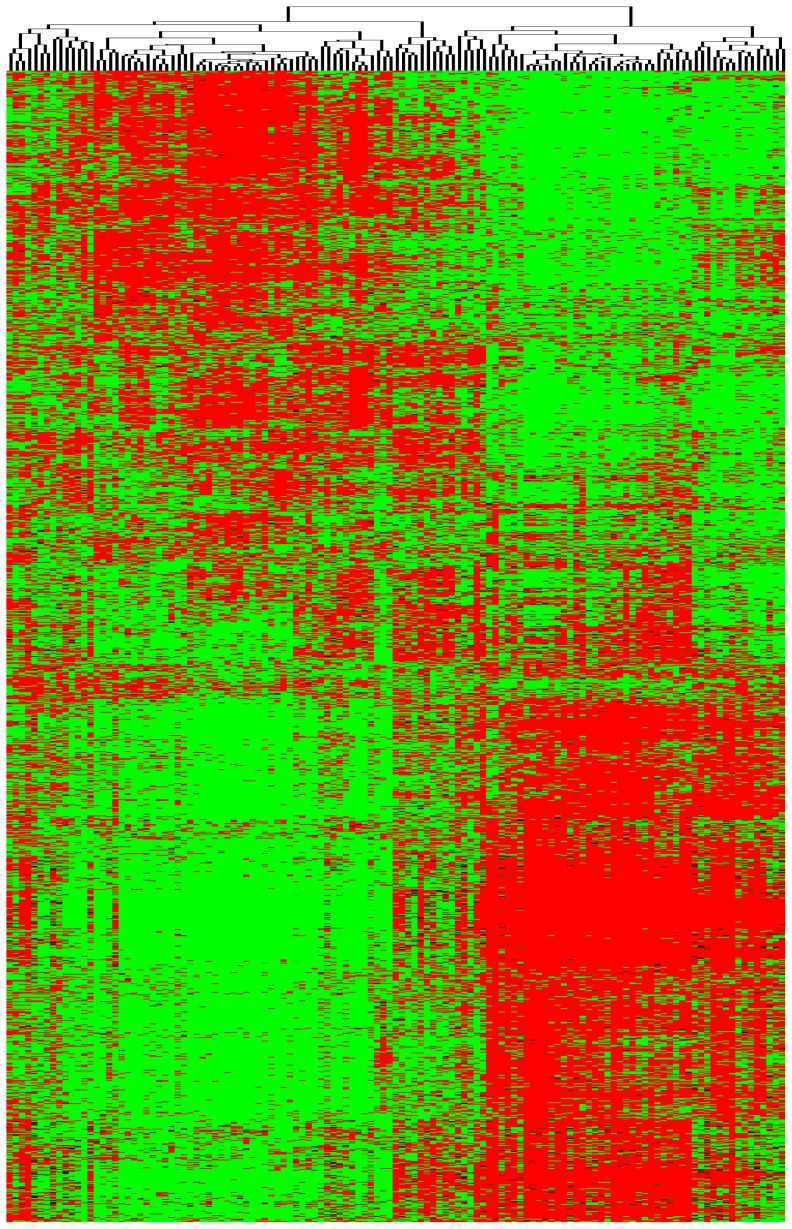
ICC subclasses. Based on expression profiles of genes in CNV-ICC-TRN, the non-negative matrix factorization–based algorithm divided ICC samples to two classes cluster I (right branch) and cluster P (left branch). This figure is heat map of differentially expressed genes between two classes.

The above biological function annotation showed that modules of CNV-ICC-TRN participated in several different aspects of biological processes. We then performed clustering analysis to check whether they can classify ICC samples into subtypes with distinct biological functions. The non-negative matrix factorization–based unsupervised clustering [25] was used based on genes of CNV-ICC-TRN, and results were confirmed by three LOOCV methods. Finally, we classified all samples in two clusters which were named cluster I (54 of 125; 43.2%) and cluster P (71 of 125; 56.8%) (Figure 5, Figure S1, Table S4 and Table S5). By using the same classification method, our result coincided with Sia et al result at the rate of 88.8% (111 of 125 matched), cluster I corresponding to their inflammation class and cluster P corresponding to their proliferation class. Signaling pathway enrichment analysis of differentially expressed genes of CNV-ICC-TRN between two clusters () suggested that these two classes had different malignancy features: highly expressed genes in cluster I were enriched to cell adhesion related pathways, such as focal adhesion and tight junction; highly expressed genes in cluster P were enriched to oncogenic signaling pathways such as MAPK signaling, Wnt signaling pathway (Table S7). These results demonstrate the potential application of our network in classification and prognosis analysis of ICC.

## Discussion

Pathogenesis studies based on gene expression profiling have evolved through several stages: single gene expression profiling; network construction and functional annotation; causal hub discovery and intervention design. Single gene expression profiling is straightforward and simple, numerous gene list signatures have been reported to either diagnose samples or predict outcome or prognosis. However it is hard to explain the functional categories of single genes. Network analysis allows structured grouping of genes, and functional module discovery can often lead to next-step research focus, which is a big progress compared to single gene profiling. The most popularly studied networks are probably the TRN and PPI (protein-protein interaction network). However functional modules in a network may still be dispersed and unconnected among each other, trying to find causal disturbances in a network has been a major goal of many computational biologists. For examples, our group have tried to develop algorithms to identify primary and secondary regulatory effects from a microRNA initiated TRN [4], have tried to identify possible hepatitis B- or C- virus protein disturbances to PPI network in hepatocellular cancer development and progression [30], [55], and we have even tried to validate causal TFs in constructed TRN by knocking out gene expression data and post-translational modification regulation data [56].

However, genetic variation was rarely considered in either our efforts or others' when trying to identify causal disturbances in a transcriptional regulation network. This probably was due to a lack of genomic sequencing and transcriptomic profiling on the same set of samples. Gene expression data alone largely prevail and bioinformatics PPI background networks are easily available too, these may have brought about some research biases in this field. However it should be readily conceived that if some functional modules in a TRN are already genetically modified, then they very likely may become the weakest points in a network that can divert the network function to adverse pathologic directions. Based on this rationale, and with the quickly increasing new generation genome sequencing data of disease samples, recently people start to investigate the genetic variation disturbance to gene expression networks. Xu et al. constructed CNV genes' co-expression network of breast cancer to study genomic variations' effect through co-expressed genes' function [9]. Zaman et al predicted breast cancer subtype-specific drug targets through signaling network assessment of mutations and copy number variations [57].

ICC is the secondly occurring liver cancer which involves a large human population, and yet it was much understudied comparing to hepatocellular carcinoma. Sia et al work represents the first comprehensive multi-level profiling of ICC samples, including RNA and SNP microarray data. Our work, based on their data, represents a primary effort to construct TRN in ICC, using our earlier developed forward-and-reverse combined engineering algorithms. Furthermore, we made another primary effort to try to identify key transcriptional modules based on their involvement of genetic variations shown by gene copy number variations. This kind of approach may bring the generally constructed TRN one step further to genetic disturbance, which may help greatly in discovering possible intervention targets for ICC. Such kind of approach can easily be extended to other disease samples with appropriate data.

On the other hand, we put forward a new method of interpreting impact of genomic variations on signaling pathways. Integrative analysis of regulatory modules and KEGG signaling pathway illustrated that the disturbance of genomic variation on signaling pathway can happen on components of pathway which was the focus of previous studies, such as variation of MAP3K7, MAP2K7 and FGFR2 in MAPK signaling, and FZD10 in Wnt signaling; but may also happen more effectively on regulators, such as variation of ZSCAN1, RFX1 which regulate SMAD proteins, the key joints of TGF-β signaling. Previous studies mostly focused on mutations in genes of signaling pathway, our studies extended to mutations in genes outside signaling pathway by integrating regulatory network. This approach broadens the way of exploring the potential impact of gene mutations.

At last, using the expression profiles of genes in CNV-ICC-TRN, we classified 125 ICC samples into two robust molecular clusters with distinct biological function features. This result at one hand helps to get insight into ICC molecular classification which is still ambiguous, on the other hand proves the application value of our innovation.

There are limitations to this early work of integrating genetic variation and TRN. We did not analyze single nucleotide polymorphisms (SNP) which may affect genes more specifically. We could not obtain clinic information to validate our subtype classification of patient samples. With the development of technology, more and more genetic variation information, such as SNP, chromosomal translocations, CNV, and so on, could be used to investigate their disturbance to TRN. On the other hand, more annotation to TRN construction itself, such as referencing protein-protein interaction relationship, kinase-substrate relationship, other post-translational modification relationship, should be carried out. Progresses in both these two directions will help in finding causal network modules and modulators. With the increment of drug-target database volume, or increase of novel drug development strategy, such kind of bioinformatics analyses which integrate genetic variation with network construction will bring experimental data closer to possible clinical intervention.

## Supporting Information

Figure S1 **Non-negative matrix factorization consensus clustering of CNV-ICC-TRN nodes' expression data from 125 samples.** (A) Consensus matrices showing internal correlation of 125 samples when 2-5 classes assumed. Red color means high robust co-clustering of samples, and clear boundary indicates good distinction among classes. (B) Plot of cophenetic coefficients distribution along different assuming numbers of classes, k. Plot shows that when k is 2, cophenetic coefficient is the highest meaning two classes assumption is the most robust.
(TIF)

Table S1 **Summary of chromosomal focal-level genomic DNA copy number alterations.** Columns cluster I and cluster P represent distributions of these focal alterations in two classes.
(DOC)

Table S2 **Full list of 33 regulatory modules of CNV-ICC-TRN.**
(DOC)

Table S3 **Enriched KEGG signaling pathways of 33 regulatory modules in CNV-ICC-TRN.** Enrichment analysis was performed using one-side Fisher's exact test, and significance threshold was FDR<0.05. Modules are represented by their regulators' names.
(DOC)

Table S4 **Samples distribution between ICC classes.** Samples distribution of our clustering result and Sia's clustering result.
(DOC)

Table S5 **Leave-one-out cross validation of ICC classes.** Modules are represented by their regulators' names.
(DOC)

Table S6 **Differentially expressed genes from CNV-ICC-TRN between cluster I and cluster P.** Differential expression analysis was tested using t-test, and significance threshold was p-value<0.001.
(DOC)

Table S7 **KEGG pathways enriched by each class's relatively high-expressed genes.** Enrichment analysis was performed using one-side Fisher's exact test, and significance threshold was p-value<0.05.
(DOC)

File S1 **Addition to the method section.**
(DOC)
