# Supplementary figures and images for: Integrative Analysis of Transcriptional Regulatory Network and Copy Number Variation in Intrahepatic Cholangiocarcinoma
Source: PLoS One. 2014 Jun 4;9(6):e98653. doi: 10.1371/journal.pone.0098653 (PMC4045758; doi:10.1371/journal.pone.0098653)

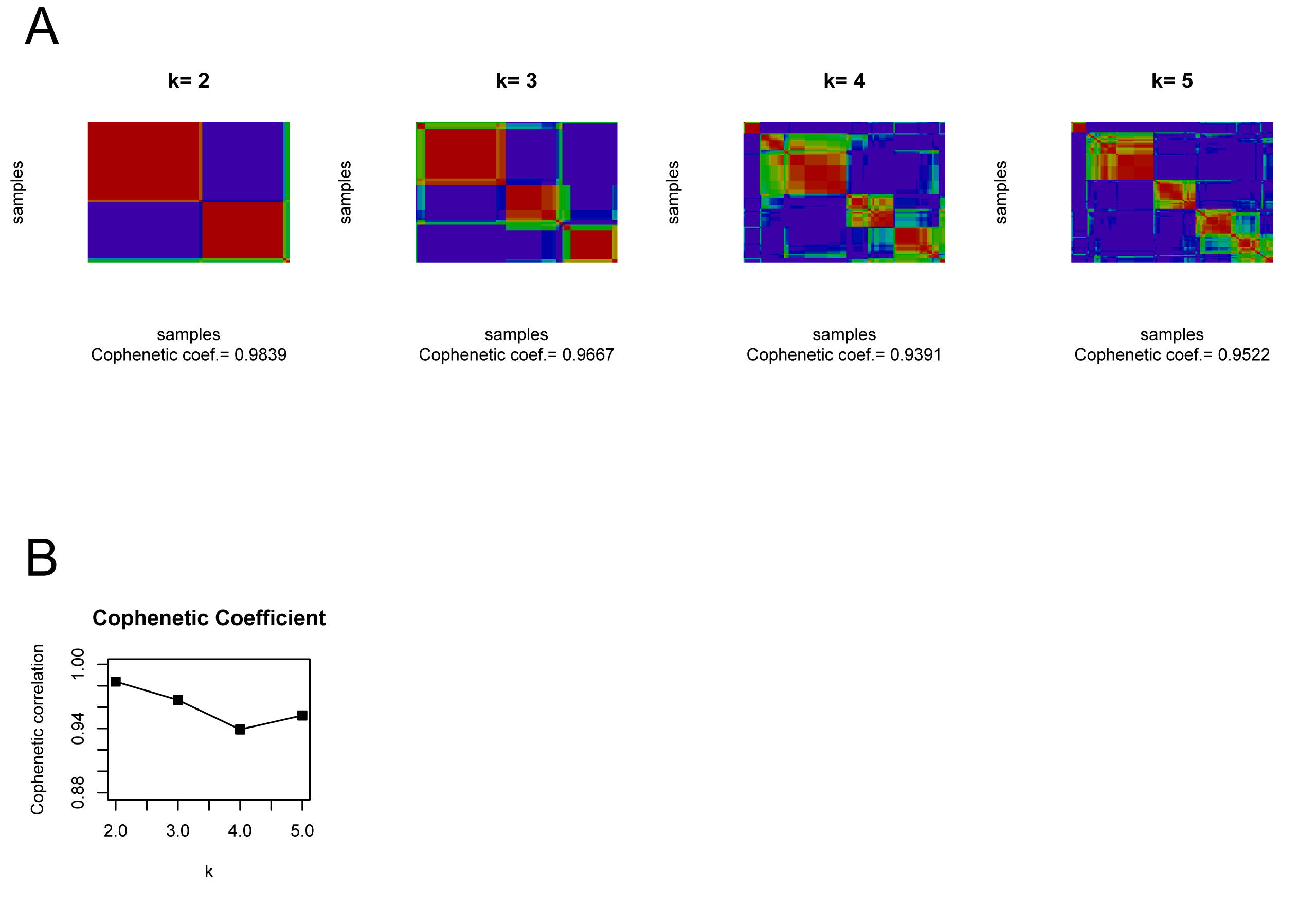

Supplement: Figure S1 — Non-negative matrix factorization consensus clustering of CNV-ICC-TRN nodes' expression data from 125 samples. (A) Consensus matrices showing internal correlation of 125 samples when 2-5 classes assumed. Red color means high robust co-clustering of samples, and clear boundary indicates good distinction among classes. (B) Plot of cophenetic coefficients distribution along different assuming numbers of classes, k. Plot shows that when k is 2, cophenetic coefficient is the highest meaning two classes assumption is the most robust. (TIF) [file pone.0098653.s001.tif]
