# Supplementary material for: Integrative Analysis of Transcriptional Regulatory Network and Copy Number Variation in Intrahepatic Cholangiocarcinoma
Source: PLoS One. 2014 Jun 4;9(6):e98653. doi: 10.1371/journal.pone.0098653 (PMC4045758; doi:10.1371/journal.pone.0098653)
Supplement: Table S1 — Summary of chromosomal focal-level genomic DNA copy number alterations. Columns cluster I and cluster P represent distributions of these focal alterations in two classes. (DOC) [file pone.0098653.s002.doc]

**SI-Table 1**.

| **Cytoband** | **Wide.Peak.Limits** | **q.values** | **Cluster I (n1=54)** | **Cluter P (n2=71)** | **All samples (N=125)** |
| --- | --- | --- | --- | --- | --- |
| **1p31.3** | chr1:64134549-64190142 | 0.0067893 | 4 | 5 | 9 |
| **1p13.1** | chr1:117422805-117569440 | 0.00019559 | 4 | 9 | 13 |
| **1q22** | chr1:153373175-153775505 | 0.0067893 | 14 | 10 | 24 |
| **1q24.2** | chr1:166956565-167090734 | 0.01585 | 6 | 12 | 18 |
| **1q41** | chr1:213336034-213384273 | 1.17E-09 | 6 | 13 | 19 |
| **7q22.1** | chr7:98618457-98668374 | 7.94E-10 | 7 | 10 | 17 |
| **8q22.1** | chr8:96827185-96893231 | 0.018818 | 7 | 4 | 11 |
| **11q13.2** | chr11:68555478-69647117 | 0.00029838 | 0 | 5 | 5 |
| **11q24.2** | chr11:126182339-126273299 | 0.00013094 | 3 | 7 | 10 |
| **12q12** | chr12:43835040-43925043 | 0.01585 | 1 | 7 | 8 |
| **20q11.21** | chr20:29628789-29667142 | 0.13038 | 0 | 6 | 6 |
| **21q22.12** | chr21:35502661-35537904 | 0.0060903 | 2 | 4 | 6 |
| **1p36.33** | chr1:1942643-2113394 | 7.41E-09 | 16 | 26 | 42 |
| **1p36.32** | chr1:2224493-2448714 | 5.55E-10 | 15 | 26 | 41 |
| **1p35.2** | chr1:1-247249719 | 1.45E-07 | 7 | 14 | 21 |
| **2q21.2** | chr2:133100326-134741235 | 0.10331 | 1 | 0 | 1 |
| **3p26.2** | chr3:1-87133686 | 0.016938 | 7 | 13 | 20 |
| **3p25.3** | chr3:1-77783730 | 0.010378 | 4 | 10 | 14 |
| **3q22.3** | chr3:138048239-138968184 | 0.14653 | 0 | 6 | 6 |
| **4q35.1** | chr4:185887816-186177454 | 0.00014581 | 5 | 8 | 13 |
| **5q33.1** | chr5:148540103-148711410 | 0.13234 | 2 | 1 | 3 |
| **6q16.3** | chr6:100167483-100943504 | 2.41E-07 | 10 | 16 | 26 |
| **6q21** | chr6:57273908-170899992 | 7.38E-06 | 8 | 13 | 21 |
| **6q25.1** | chr6:151464649-151795965 | 1.34E-06 | 6 | 12 | 18 |
| **6q26** | chr6:161612277-163093667 | 1.53E-06 | 5 | 13 | 18 |
| **7q36.1** | chr7:151773466-152160810 | 0.0010798 | 1 | 3 | 4 |
| **8p23.1** | chr8:1-38441502 | 0.02865 | 4 | 9 | 13 |
| **9p21.3** | chr9:21855843-22441030 | 8.31E-14 | 9 | 13 | 22 |
| **9p21.3** | chr9:22441032-25673219 | 0.025331 | 7 | 9 | 16 |
| **9q21.13** | chr9:74165313-74712654 | 0.03831 | 4 | 7 | 11 |
| **10q24.32** | chr10:103306809-103573715 | 0.0046648 | 3 | 5 | 8 |
| **10q26.13** | chr10:64222249-135374737 | 0.045596 | 2 | 3 | 5 |
| **10q26.13** | chr10:126092887-126442031 | 6.49E-05 | 2 | 7 | 9 |
| **12q24.33** | chr12:75909029-132349534 | 0.078369 | 4 | 2 | 6 |
| **13q12.11** | chr13:1-114142980 | 0.26104 | 6 | 12 | 18 |
| **14q22.1** | chr14:50157014-50265114 | 2.08E-08 | 4 | 11 | 15 |
| **14q32.32** | chr14:78762184-106368585 | 0.0028172 | 8 | 14 | 22 |
| **15q26.1** | chr15:88096965-88183623 | 0.010203 | 2 | 3 | 5 |
| **16q24.3** | chr16:83340267-88654580 | 0.10881 | 2 | 8 | 10 |
| **17p13.3** | chr17:3137284-3249737 | 0.0097996 | 4 | 5 | 9 |
| **19p13.2** | chr19:1-63811651 | 0.1481 | 1 | 1 | 2 |
| **19p13.11** | chr19:17040790-17260475 | 0.046836 | 3 | 2 | 5 |

**Summary of chromosomal focal-level genomic DNA copy number alterations.**

Columns cluster I and cluster P represent distributions of these focal alterations in two classes.

“Wide.Peak.Limits” represents boundaries of significantly aberrant regions.
