# Supplementary material for: Integrative Analysis of Transcriptional Regulatory Network and Copy Number Variation in Intrahepatic Cholangiocarcinoma
Source: PLoS One. 2014 Jun 4;9(6):e98653. doi: 10.1371/journal.pone.0098653 (PMC4045758; doi:10.1371/journal.pone.0098653)
Supplement: Table S2 — Full list of 33 regulatory modules of CNV-ICC-TRN. (DOC) [file pone.0098653.s003.doc]

**SI-Table 2**.

| **Module** | **Size** | **Type** | **Target** |
| --- | --- | --- | --- |
| **AHR** | 144 | CNV-genes-only enriched | ACTB, AGPAT9, AHCYL1, AKR1B10, ALDH3A1, AMACR, APLN, ARF4, ATP6AP2, BAD, BAP1, BCOR, BNC2, BRS3, C10orf114, MEIOB, C1GALT1, CAMK2A, CAMK2D, CCT8, CDC42EP5, CDC5L, CHMP5, CNBP, COLEC12, CREB3L1, DGKG, DIP2C, DSTYK, DTNA, DYNC1I1, DYNLL1, EAF2, EDEM2, EFNB2, EGLN3, EID2, EWSR1, DENND6A, FAM20B, FAM76A, FGF12, GALNT18, GDF7, GPR176, GRM6, HECA, HIST1H2AA, HIST2H2AC, HNRNPR, HOMER2, HS3ST3A1, HSP90B1, HSPA8, HTR5A, IER3IP1, INPP4B, INSL3, IWS1, KCNA4, KCNH3, KHDRBS1, EMC1, KLF4, KLF5, KLHDC2, KTI12, LAMB1, LIMA1, LMO4, LRRC59, MARCKS, METTL6, MFSD1, MORC2, MPZL2, MTF2, N4BP3, NACA, NCL, NFAT5, NFE2L2, OR5D16, PDCD10, PDE6D, PDIK1L, PDP1, PIAS3, PIM2, PKN2, POU3F2, PPARA, PPP3CA, PRDM8, PSMD9, PTGER4, RAB6A, RAC1, RASGEF1B, RBBP4, RBFOX1, RELN, RNASEH2A, RNF149, RNF214, RNPEP, RP2, RPL27A, RPS13, RPS6KA4, S100PBP, SASS6, SAT1, SEC11C, SH3BP4, SIPA1, SKIL, SLC12A6, SLC36A1, SLC6A4, SLK, SP4, SPAST, SPI1, TC2N, TCEB1, TCF4, TCTA, TIAL1, TIAM2, TIMM50, TMEM164, TMPO, TSPAN13, UBA6, UBE2E3, UBE3A, UBP1, USP19, YARS, ZC3HAV1L, ZEB1, ZFR, ZNF785 |
| **TFAP2A** | 90 | CNV-genes-only enriched | ADAM12, ADAMTS2, ANKRD28, ATP6V1A, ATRIP, BCL3, BCR, BMP1, BOLA3, TMEM259, CAMSAP1, CAMSAP2, CANT1, CCDC85B, CDC37L1, CLIP1, CLTB, CMTM3, COQ10A, CREBBP, DBN1, DDR1, DRAM2, ELOVL1, EPB41L1, EPB41L2, FAM155B, FOXJ3, GADD45GIP1, PAXBP1, GRIN1, HCFC1, HDAC4, HNRNPA3, IKZF1, IQGAP3, IRF1, IRF2BPL, VWA8, KLF3, LPCAT3, LPP, MAD2L2, MEGF9, MGAT4B, MON2, MYC, NFKB1, NRARP, PDE7A, PIBF1, PPP6C, PTMA, PTPN11, RAB10, RAB2B, RAB35, RARG, RBM25, RHOA, RNPC3, ROCK2, SCAF1, 5-Sep, SLC22A15, SLC25A28, SLC9A2, SMAD7, SOCS4, SOX9, SP4, SPAST, SPRYD3, SUMO2, SUV39H1, THOC7, TMEM178A, TNFAIP8, TPM1, UBE2N, UBE2Q1, UBE2Q2, UBL3, ULK1, USP15, VEZF1, WSB2, XPO4, YAP1, ZSWIM4 |
| **TFAP2B** | 76 | CNV-genes-only enriched | AARS2, ASIC2, ADAM10, ADCY8, FAM219A, CACNG4, CACNG7, CBLL1, CDKN1C, CDX2, CLU, CYC1, DBC1, DLC1, EIF4G2, AMER2, FAM135B, FBXO11, FGFRL1, FUCA1, GATAD2A, GPC3, GREB1L, GRID1, HHEX, IGDCC3, IRF1, ITFG1, AJUBA, KDM5B, GSE1, KRT8, KRT9, LHX9, LPCAT1, LRFN5, LSM11, MAP4K3, MEX3C, NR2F6, NSD1, OTUD5, PABPC1L2B, PAX3, PHF21A, PICALM, PITRM1, POU4F1, POU4F2, PPM1A, PRICKLE4, PTMA, PTPN3, PTTG1IP, RFX1, RNF152, RUNDC3B, SCN1B, SCN5A, SEZ6, SIX2, SPECC1, SPO11, ST8SIA3, SYVN1, TCF3, TDRD10, TRHDE, TSSK3, TTLL7, UBTF, UHMK1, ULK1, WNT16, ZNF664, ZNF771 |
| **ARNT** | 176 | CNV-genes-only enriched | ADAMTSL3, ADPRHL2, AIFM1, ANKRD37, ANKRD40, ANKS6, ANP32A, AQP3, ARL5A, ATF6B, ATP6V0C, BAHD1, BLOC1S1, BPIFB1, C11orf84, C19orf26, C3orf17, C4orf21, CABLES1, CALR, CAND1, CBX7, CDC42EP3, CHCHD6, CHD3, CPSF7, CREG2, CROCC, DCHS1, DHCR24, DIO3, DRD1, EDEM3, FAM134C, FANCL, FBXO7, FEV, FGFR1OP2, FLI1, FOXO4, GALNT14, GAP43, GINS1, GNAZ, GNMT, GPBP1L1, GRIA3, GRM5, GSK3A, GSR, HACE1, HDGF, HEXIM1, HIPK1, HIRA, HIVEP1, HIVEP2, HMBOX1, HNRNPH2, HOXC11, IGFBPL1, IKZF2, IPMK, ITPKC, KANK4, KAT5, KCNA4, KCND1, KCNS2, KIAA0586, KIAA1244, KIF1C, KRT81, KRT85, LAPTM4A, LINC00602, LRRC24, LRRC37A3, LRRC55, MAP3K10, MESDC1, MET, MEX3B, MIEN1, MXD3, NDNL2, NGFR, NME1-NME2, NOP58, OPN3, OPRD1, OR5M9, PAN3, PARP14, PDE4A, PFDN2, PIGA, PIP5K1A, PLAA, PLCB4, PLCL1, PLXND1, PMVK, POU6F1, PPARGC1B, PRDM10, PRPF4B, PRR3, PRRG4, PTGER2, PTP4A1, PTPN3, QPCT, RAB11FIP1, RAB38, RGS16, RGS9BP, RIC8B, RLF, RNF128, RNF165, RPIA, RPS6KA5, SART3, SDC1, SEMA4C, SENP3, SLC1A2, SLC26A2, SLC27A4, SLC43A1, SLCO5A1, SNAPIN, SOAT1, SOCS3, SPSB4, SREBF2, STAG2, STK32C, SWT1, SYT7, TENC1, TERF2IP, TFAP2D, TGIF2, TPRA1, TREH, TRIAP1, TRIM11, TRPC4, TSPAN14, TSR1, TUBA4B, TUBB4B, TXNIP, UBE2Q2P1, UBE2R2, USP15, UVRAG, VAV2, VMAC, VPRBP, WDR81, WDR83, NELFA, WSB1, XKR4, YTHDF3, ZBTB45, ZBTB7A, ZDHHC23, ZNF131, ZNF341, ZNF384, ZNF777, ZZEF1 |
| **BACH2** | 24 | CNV-TF-only regulated | ADPRHL2, ANAPC2, ATL1, C15orf48, C1orf43, COL5A1, DTL, FBXO11, GPX3, HIST2H2BE, HOMER2, IL1RN, IL6R, LAMC2, MET, NME1, PERP, PPP1R13L, S100A3, SRPX2, TCTEX1D4, TMED10, TTC19, USP49 |
| **BAX** | 12 | CNV-TF-only regulated | ALDH1A2, COL4A5, INHBA, MLF2, MSH2, MSRA, NRAS, OR11H6, PPT2, PSMC4, RAB2A, ZNF396 |
| **CEBPA** | 75 | CNV-TF-only regulated | ABCC6, ADSS, AHCY, ATP2B2, ATP5G1, C4orf19, C9, CEBPG, CES2, CES4A, CLDN1, COL4A3, CUL7, CWF19L2, DCAF6, DLGAP4, DPYS, DUSP13, EFNA1, EFS, ERBB3, FAM161B, FGB, FZD5, GAST, GATA4, GBX2, H3F3AP4, HERC2P4, HINT1, HMGA2, IKZF5, IL1A, INHBE, IQGAP2, KLHDC10, KRT81, KRTAP4-1, LTB, MAP7, MBNL2, MID1IP1, MLLT11, MTTP, N4BP2L2, NBR2, NCK1, NEUROD2, OR5M8, OR6B3, ORC4, ORC5, OSBP, PPP1R14C, PPP1R1A, PTPN3, RNF115, RPS6KA3, SEPHS2, SERPINC1, SFXN5, SIRT5, SLC25A12, SLC37A4, SPINK5, TAC4, TAF11, TBC1D9, TESK2, TGIF1, TPK1, TPM3, TRHDE, URGCP, VTN |
| **CREB1** | 183 | CNV-genes-only enriched | ASIC4, ACO1, ACTL6B, AK3, ANAPC16, ANKRD54, APOLD1, APPBP2, ARRDC5, B4GALT3, BAZ2B, BIRC6, BRD2, BSCL2, C15orf37, CMC2, C17orf104, TMEM256, TPGS1, C7orf41, C7orf50, C7orf55, CALCA, CAMSAP2, CAT, CDC42EP4, CDK2AP2, CHD4, CHKB, CHST8, CLCN6, CRTC1, CRY1, CSAD, CTNNA1, CUL4B, CYHR1, DALRD3, DAZAP1, DCUN1D4, DDX5, DNM1, DPYSL2, DUS4L, E2F3, ECE1, EID2B, EPS8L2, ESYT2, FAF2, FAM117B, FAM120A, FAM155A, FAM189B, FBXL5, GAL, GAS2L2, GHR, GLOD4, GMFB, GPN1, GPSM1, GSK3B, GTPBP4, HARS2, HAUS2, HCFC1, HDAC4, MROH7, HOOK2, HYOU1, IKZF1, ILVBL, ITFG2, ITGA5, ITGB1, JOSD1, KCNH6, KCNQ2, KANSL1, LEMD2, LOC595101, LONP1, LYRM1, LZTR1, MAPK8IP2, MAPKAPK3, MCRS1, MDGA1, ME3, MLH1, MMP28, MOK, MON2, MRFAP1L1, MRPL28, MTSS1, MYH10, NCALD, NCDN, NEURL, NLRX1, NUDT3, NUFIP2, OR2A25, OSBP, OXR1, PAF1, PAPOLA, PCDHA7, PCM1, PCNA, PHF23, PI4KA, POLE3, PPIC, PPM1K, PPP6C, PPRC1, PRMT10, PRPF6, PTGES2, PTMA, PTMS, PYGO2, RAB3GAP1, RAB7A, RAD23A, RALGAPA1, RBM25, RBM47, RHBDD1, RHOQ, RIPK4, RPP21, RPS25, RPS9, RPUSD2, SAMD4B, SCAMP1, SCYL2, SEPHS2, 2-Sep, SH3GLB1, SH3PXD2A, SHOX2, SLC11A2, SMAD1, SMARCA1, SNORD56, SP4, SRP68, SRRM2, SSNA1, TAF10, TAF15, TCEAL3, TCF25, TJAP1, TMED5, TMEM151A, TMEM237, TMEM82, TMEM9B, TP53, TPM3, TRMT2A, TSPAN31, UBE2G1, UBE2Q1, UBXN1, UNCX, USP31, VEZF1, VPS37B, WDFY1, XPO4, XRN2, XYLT2, YWHAG, YY1, ZDHHC9, ZNF622 |
| **DAND5** | 153 | both CNV-TF regulated and CNV-genes enriched | AAMP, ABP1, ADCY8, AFAP1, AGAP3, AGBL4, AGFG2, AGPAT9, AHCYL1, AHCYL2, AHR, AHSA2, AKAP1, AKT1S1, AQP1, ARL13B, ARRB2, ASPH, BAG1, BRSK1, SZRD1, C1orf86, C20orf111, MGARP, CBLN3, CCNK, CDKL2, CIAO1, CLIC3, CNN2, COL5A1, COX19, CPXM1, CREB3L1, CRELD2, CTHRC1, CYTH1, CYTH3, DACT2, DCBLD1, DDI2, DDIT4, DDX5, DENR, DICER1, DNAH11, DNAI1, DNAJA1, DPP10, DSCAML1, DYNC1H1, DYNC1I1, EMP3, ENTPD7, F3, FAM89A, FBXO24, FGFR3, FKBP2, FLJ22184, FOSL1, GABRQ, GDF7, GJB2, GJD2, GNG12, GRID1, HDAC6, HOXA4, HPRT1, IDI1, IMPA1, ING2, IP6K2, ITGA5, ITGA6, KCNB1, KCTD21, KLF5, KRT17, LIMK2, LINC00303, LPCAT2, LRRC37A2, LY75, MAF1, MAGI3, MAP3K7, MICALL1, MIER1, MON2, MRPS28, MSL3, MSN, MTFR1, MXD1, MYH7B, MYH9, NFKBIB, NFYA, NOMO2, NTPCR, PANK4, PARD6G, PCCA, PFKFB4, PIM2, POLE4, POLR3G, PPP2R3A, PRKCD, PSEN1, PTPRF, PYGL, RBBP7, RCN2, RGS10, RMI2, RNGTT, RPL27A, RPS10, RUFY4, RUNDC3B, S100PBP, SEMA4B, SERPINH1, SERTAD1, SFMBT1, SHOC2, SLC20A1, SLC5A3, SLC7A5, SLC9A3R2, SNTB2, SNX22, SOX12, SPCS1, SRR, SRSF2, SYNGR1, TMED10, TPM2, TSKU, TUBA1C, UBP1, UGDH, USP49, USP6NL, WDR91, WFS1, WTAP, YPEL5, ZBTB14 |
| **E2F1** | 128 | CNV-genes-only enriched | ADPRHL2, ADRM1, AKT1, AMIGO1, AMOTL2, ANP32E, ARF6, ARFIP2, ARHGAP44, ATF6B, BAZ1B, BCL6B, BCL7A, BTBD9, CAP1, CBFB, CBX1, CCDC124, CHD3, CHST1, CHSY3, CLDN4, COL4A2, CPB1, CPSF7, CSGALNACT2, CXXC4, DDX19B, DHH, DLG1, DLG5, DNMT3A, ENOX1, ERC1, FBXL7, FBXO42, FBXW7, FKBP10, FOXO1, FUT11, GAL3ST4, GALR3, GDF11, GDNF, GPR158, GPRIN1, GRIN2A, GRM5, GSC, GSC2, GSK3A, GTPBP1, H1FX, H2AFX, HIRA, HMGA1, HN1, IDH3G, IGFBPL1, INO80, IRF5, ISL2, JDP2, KCNA7, KCND3, KCTD1, KDM2B, KIAA0141, KIAA0355, KLF10, KLF12, LIN7A, LMBR1L, MAN2C1, MAP2K7, MAP3K10, MDFI, METTL15, MRI1, MXI1, NFATC1, NKX6-1, NMT2, NOP2, NUMBL, PCSK1, PIK3R3, PKP3, PLCL1, PLXNC1, PMM1, PPP5C, PRRT2, QSER1, REEP1, RER1, RIMS2, RNF168, RPL26L1, RPL37, SCFD2, SDC1, SIK2, SLC39A7, SPSB4, SRCAP, SS18L1, SYNPO, TAL1, TAOK1, TAOK2, TAP1, TCEAL5, TIMP2, TLX3, TPCN1, TRPS1, UNC5C, VAT1, WDR65, WDR73, WDR83, WNT9B, XIAP, ZC3H7B, ZCCHC7, ZNF444, ZNRF1 |
| **ESR1** | 47 | both CNV-TF regulated and CNV-genes enriched | ACTN2, ARHGAP1, ARHGEF18, C1orf52, C1orf85, CDH24, CHRM4, DDB1, DMD, DNAJB2, EIF5B, FAP, FRAS1, GRIN2C, H2AFJ, HDAC4, HHATL, ICA1, IL10, JOSD1, KANSL1, LAMTOR1, MRPS2, NAA15, NAGPA, NDUFB8, NPEPL1, NR2C2AP, OPN3, PITPNM1, POU3F4, POU6F2, PPME1, RARG, RDH10, RHOQ, RNF123, RRAS, RSPRY1, SAE1, 9-Sep, SMARCC1, SMG5, SUV39H1, TPM4, TUBA4A, ZFAND2B |
| **ELK1** | 102 | CNV-genes-only enriched | ADO, AGFG1, AKT1, AKTIP, APBA3, ARHGAP1, ASCL1, ATP6V1B2, BCAP31, BIN3, C19orf26, CAMSAP2, CBLB, CDYL, CHPF, COMT, CYB561D2, DDX31, DNLZ, E2F4, FAM63A, FAM96B, FICD, GMFB, H2AFY, HES6, HNRPLL, KANSL1, LAMTOR1, LEPROTL1, LOH12CR1, MAF1, MAP3K11, MARK2, MAT2A, MBTD1, MCM6, MCRS1, MCTP1, MON2, MSH3, MTA2, MTOR, NACC2, NCOA7, NPM3, NTHL1, NXT1, ODF2, PACSIN2, PCDHB8, PDAP1, PER2, PHLDA3, PLSCR3, POLE3, POLR2K, PPP1R11, PPRC1, PRKG1, PSMA6, PTMS, PUF60, RAB11A, RAD23A, RRAGA, RRAS, SCFD2, SIX5, SLC35B2, SLC43A2, SMARCD1, SNTB2, SPATA22, SRM, SYS1, TFCP2, TIGD6, TMED2, TMEM208, TMEM59, TMEM99, TMEM9B, TMOD3, TPSD1, TTC16, U2SURP, UBE2Q1, UBR7, URM1, USP11, USP4, USP9X, VPS37C, WBSCR16, WDR46, XPO5, YME1L1, ZCCHC7, ZDHHC14, ZNF362, ZRANB2 |
| **HNF4A** | 65 | CNV-genes-only enriched | ABCC6, ACY1, ADCY3, ADCY6, APOB, ARRB1, ATP6V1A, AUP1, BCL7C, BIRC6, KANSL2, TMEM259, C3orf18, CPED1, CAMSAP3, CDHR5, CRIP1, DCAF7, DEPDC7, DPYSL2, ENPEP, F10, FADS3, FAM120A, GMFG, GPX2, HNF1A, HNRNPUL1, IRF1, JUN, KIAA1161, KPNB1, LONP1, MBTD1, MPZL2, MTPAP, MYL12A, NDUFB10, NOX4, NR0B2, NR5A2, PCBP2, PCDH7, PJA2, PKLR, PKN1, PPP2R2A, PTPRCAP, PVRL3, RBM25, RYBP, SCAMP3, SLC25A28, SLC35D1, SLC40A1, SRFBP1, TFR2, TM4SF5, TMEM158, UBE2E1, UBE2Q1, UBXN1, WDR45, XPO4, XXYLT1 |
| **MAX** | 120 | CNV-genes-only enriched | ACACA, ADAM12, AKAP1, ALKBH1, ANKRD11, ANKRD12, B3GALT6, B4GALNT4, BACH1, BCL9, BEND3, BNIP3, SLC35F6, CELSR1, CLCN6, CLN3, CTSK, DVL2, ELK4, ELOVL3, ELOVL7, FAM155B, FBXL2, GON4L, HAL, HHEX, HNRNPA1, HOXB7, ILVBL, KIAA0232, KLF9, LDLRAD3, LGALSL, LHX6, LSAMP, METTL3, MFNG, MKS1, 2-Mar, MOXD1, MRRF, MUS81, MYRIP, NAA15, NKX6-2, NOTUM, ONECUT1, ORC4, OVOL1, PLEKHM1, PPAPDC1A, PPP1R26, PTGER2, PTP4A1, RABAC1, RAI1, RNMT, RSPO2, SELL, SIX3, SLC16A11, SLC39A9, SLC6A11, SUV39H1, TCF7L2, TIPARP, TMEM201, TMEM5, TRAPPC8, UBE2B, UBXN10, ZDHHC8, ZNF507, ZRANB3, ACSF2, ARHGEF35, BAG3, C1orf43, C7orf25, CEACAM7, CSTF3, EIF3G, ENPP2, PCED1A, FBXO27, FFAR2, FZD5, GAS7, GKAP1, GTF2H1, IRX1, ITPR1, KCNK12, LRRN4CL, MAT2A, MCRS1, MIF, MTDH, PANK3, PDZK1, PGAP2, PNISR, PPP2R5C, RNASEK, RPL23A, RRP15, SLC25A23, SLC9A2, SUB1, SUGP2, TBC1D9, TEX261, TGFB2, TIMM8A, TMEM108, TMEM204, TRIM71, TSN, ZBTB44, ZZZ3 |
| **RFX1** | 92 | CNV-TF-only regulated | ABI2, ABI3BP, AMDHD2, AMMECR1, ARL4A, ARL5A, ATF3, ATP6V0D1, BCR, BRIX1, KIAA1211L, CALM2, DDX6, DNAI2, DNAJB5, GPATCH2, HSP90B1, IFT74, NAA50, NFIA, NOTCH1, OTUD6B, PAFAH1B2, PCDHAC1, PCGF1, PHF15, PHKB, PRDX4, RAB10, RAB6A, RNGTT, RSL24D1, SLC25A46, SMAD7, TRIM27, TSPAN6, TUBD1, ZFX, ALMS1, APC, ARHGAP9, BCL9, BRWD3, TPGS2, C1orf65, C3orf30, CCDC64, CLPX, DNAJC10, DYNLL1, EFNA5, EIF3J, FAR2, FEV, GCK, HEMK1, HSD11B1L, HSPBAP1, HTR1B, IRS1, ITGB1, LYSMD1, MED12, MFNG, MTR, MUS81, NR2F6, OR10G2, PRKCG, PSMA4, PTPRA, PTPRR, RAD21, RAPGEF4, RNPC3, RPL36AL, SLC25A13, SLC27A3, SMARCAD1, SNRNP27, SRSF2, STXBP5, TADA1, TLCD1, TNNI1, TSC1, TXNDC17, UBE2N, ZBTB40, ZCCHC8, ZFP36L1, ZFR |
| **MYCN** | 62 | CNV-genes-only enriched | ALKBH5, ATP6V1A, BEX2, RHNO1, MGME1, CABP7, CD164, CELF1, COMT, COX5A, CRCT1, CSK, DLX1, E2F4, EIF3H, ERCC8, ETV1, FBL, GNL3, GTF2A1, HNRNPA1P10, HNRNPR, HTR4, JOSD1, KLF11, KLHL35, KPNB1, LAMTOR1, LMNB1, LMNB2, MAPRE1, MBTD1, MID1IP1, MRO, MSN, NAA15, NAT10, NCL, NOC4L, PLA2G4A, POGK, PPAT, PRPS2, QSOX2, RAN, RASSF3, REV3L, RHOA, RUNDC3B, SLC33A1, SLC38A7, SMARCA4, SNRPA1, SRSF9, STRAP, SUB1, TRIM8, UBE2Q2, USP13, USP9X, VKORC1L1, VWA2 |
| **MZF1** | 159 | both CNV-TF regulated and CNV-genes enriched | ACTR3, ADAMTS15, AFF4, AK3, AKAP1, AP1S1, APPBP2, ARID4B, ARMCX3, ATP1B1, BAZ1A, BCL3, BCL9, C14orf119, TMEM256, LURAP1, TMEM74B, PTCHD4, C6orf62, CADPS2, CBX5, CCNI, CHD7, CLASP1, CNKSR2, CREB1, CTDSPL, CUEDC1, CXXC5, DAB2IP, DCAF6, DCUN1D4, DDX5, DDX6, DENND4A, DMPK, DPP6, E2F3, EDF1, EIF4ENIF1, EIF4G3, EIF5B, EPHB2, FAF2, FAM117B, FGFBP3, FGFR2, FKBP11, FKBP9, FRYL, GALNT4, GHR, GSK3B, H3F3B, HCFC1, HHATL, ID4, IGFALS, KCNK12, KCNQ2, KCNS3, KCTD15, KANSL1, KLHDC4, LMO3, LUC7L3, LYRM1, MARCKS, MARVELD3, MAX, MIDN, MRM1, MRPL2, NCALD, NCOR1, NFATC2, NGFRAP1, NHS, NIPBL, NOTCH2NL, NSD1, NUFIP2, ONECUT3, ORAI3, OSBP, OTX2, PABPC1L2B, PAK3, PAPOLA, PCBP2, PEA15, PHF23, PITX3, PLCXD3, PLXNB1, PPM1B, PRDM14, PSMC5, PTPN4, PVRL1, PVRL3, RASSF10, RCE1, RDH10, REL, RNF19A, RORA, SAMD4A, SEC63, 2-Sep, 9-Sep, SLC25A13, SLC25A28, SLC9A2, SLC9A3R1, SMG5, SNORD56, SOX4, SPECC1, SPHK2, EPPIN, SPRYD3, SPTBN1, STAT6, SUGP2, TAF15, TCEAL8, TCERG1, TCF12, TCF25, TFE3, TJAP1, TLK1, TMTC1, TNRC6B, TOB1, TRIM39, TRIM71, TSC1, UBAP2L, UHRF1, UPF2, UPRT, VEZF1, WAPAL, WIBG, WNT3, YY1, ZBTB20, ZDHHC1, ZDHHC20, ZDHHC5, ZFAND2B, ZFP91, ZHX2, ZMAT1, ZMAT5, ZMYND11, ZNF821 |
| **NFIC** | 52 | CNV-TF-only regulated | B3GALT6, B4GALT5, BTBD1, BUD13, WBP1L, FAM213A, RHNO1, CAMTA2, COX11, DOCK9, E2F3, FAM19A5, HDAC11, HIST1H2AH, HIST1H2AJ, HIST1H2BO, HIST3H2BB, KCNK1, KRT17, LOC595101, LONP1, LTB4R, LTBP3, MBD3, MICALL1, MIF, NTN1, OR5D18, PARVB, PCBP4, PCDH7, PDE5A, PIAS4, PREX2, PTPLAD2, RBBP4, RBBP7, RSBN1, SCARF2, SEMA6C, SLC16A13, SLC25A29, SMAD7, ST5, STT3B, TM9SF2, TOP3A, TRPC4AP, TRUB2, TSC22D2, WDR12, ZBTB16 |
| **NFKB2** | 33 | CNV-TF-only regulated | AMPD2, ARID5A, B9D1, BCL6B, BCOR, C3orf17, CCNL1, CHID1, DZIP1L, FAM134C, GANAB, HIVEP2, HNF1A, IKZF3, JAK1, KIF3C, MAP2K3, MAP3K8, MELK, NFATC1, P2RY10, PDE2A, POU2F2, PPP2R5D, PTPN2, RAP2C, RNF220, ROCK1, SAP130, SBF2, SEZ6L2, SUPT7L, XIAP |
| **NFYB** | 67 | both CNV-TF regulated and CNV-genes enriched | ABCB6, ADHFE1, BNIP3, KANSL2, TMEM256, CAT, CCNB1, CDC23, CYB5B, CYP2R1, DNMT3B, DPYSL2, DUSP19, ELMOD2, ERRFI1, ETF1, FAM162A, STRIP2, FAM73B, GGH, HIBADH, HMGB1, HNRNPUL1, HSPA1A, IBTK, INCENP, ING2, KCTD9, KIF5B, 2-Mar, MSH6, MYH10, MYH9, NDE1, NUP214, NUP62CL, OSBP, PAF1, PDIA4, PDP1, PGP, PIM1, PPP1R15B, PPP2R1A, PTMA, RAB2B, RAB40C, RPS9, SLC25A13, SLC2A1, SNRPF, SPAG9, STMN4, SUN2, TBX19, TMEM180, TOB1, TPCN1, TSC22D1, TSPAN3, UBB, UBP1, UBTF, WDFY1, YARS, ZDHHC5, ZSWIM4 |
| **PATZ1** | 92 | CNV-genes-only enriched | ABCA1, ACTG1, ADCY6, AGAP1, AGPAT1, AMPD2, ANAPC16, AP1S1, ARAP1, ARRB1, C11orf35, CACNA1D, CAMSAP3, CCNJL, CDK2, CHL1, CIC, CLMN, CNN3, CNOT3, CREB1, CSRNP2, DAB2IP, DBN1, DBNDD2, DHFR, DYNLL2, EBAG9, ECE1, ERGIC1, FAM83H, FMNL1, FZD10, GABARAP, GPR153, GPRC5B, GTF3C4, HIF1A, IGF2BP1, INPP5F, IQGAP1, IRX1, ITGA3, ITGB1, FAM214B, KIF1A, LHFP, LMF1, MAGI3, MAPK8IP1, MAPKAPK3, MARCKS, MBD3, MPST, MYL12A, NCKAP1L, NFE2L1, NOTUM, PAX8, PITPNM3, PLEC, PNISR, PPFIA3, PRDM16, PRPF40B, PRRT2, PSME4, PTGES3, PTMA, PWWP2B, RAB8B, RABAC1, RARG, RTN4RL2, RUSC1-AS1, SBF2, SCAMP2, SHANK3, SMARCD3, SPOP, SYNGR1, TEF, TSHZ3, TXNDC12, UBP1, UPK3A, VAV2, VEZF1, VPS35, WIPF2, WT1-AS, ZBTB4 |
| **PAX5** | 153 | CNV-genes-only enriched | ABL1, ACVR1, AP1S1, BCL9L, BIRC6, BTBD1, TMEM256, C1orf43, C1orf50, C3orf58, CCZ1B, C9orf41, CAMK1D, CAMK2N2, CCDC74A, CCNK, CEP44, CMPK1, CNOT2, CNOT7, CRAMP1L, CREG2, CUL4B, CWC15, DCUN1D5, DDB1, DDX6, DMTF1, DR1, E2F4, EFCAB4A, FAF1, FBXO2, FBXW9, FNDC5, FOXL2, GJD2, GNL3, GOLGA4, GSK3B, H3F3AP4, HAUS2, HCFC1, HIST1H2AK, HIST2H2AC, HSPB9, ICA1, INPP5A, IRF2BPL, KCTD12, KCTD6, KHDRBS1, KLF5, LEMD3, ATP6V0E2-AS1, MALT1, MARCKS, MED8, MEX3B, MINPP1, MRPS18B, MST4, MTDH, MTMR3, MTMR4, MYO1E, NDUFB3, NFKBIB, NFKBIZ, NR6A1, NSUN2, NTF3, NUP98, TENM1, PALM2, PCDHGB6, PDF, PGAP2, PHF20L1, PIGV, PIP4K2B, PITX2, PJA2, PLCB3, PLEKHA5, PLEKHH3, PNO1, POLG2, PPA2, PRIMA1, PTGES3, RAB35, RAB7A, RALBP1, RAP2A, RAPGEFL1, RBBP7, RBM12, RFWD2, RHBDL2, RHOA, RNPC3, RNPEP, RPS10, RTF1, RUSC1, RUVBL1, SCAMP1, 10-Sep, 7-Sep, SH3PXD2A, SIX5, SLC32A1, SMAD7, SMARCE1, SNHG10, SNX7, SP4, SPATS2, SRD5A1, SSTR5, SUMF1, SUMO1, SYNC, TBC1D20, TBC1D2B, TCEB1, TCERG1, TCF7L2, TIPARP, TMED7, TMEM145, TMEM59, TNXB, TRA2B, TRAPPC4, TRIM3, TRPM3, TSC22D2, TSPAN31, TTC3, TWF1, UBE2Q2, UBXN4, UHMK1, WBP1, YTHDC1, YWHAG, YY1, ZC3HAV1L, ZCCHC16, ZFR, ZNF622 |
| **POU3F2** | 43 | both CNV-TF regulated and CNV-genes enriched | ACTRT1, BIRC6, BUB3, DYNAP, C9orf78, CD36, CLCA3P, CLLU1, CPNE1, CUL4B, DCX, DYNLL1, FCHO1, FRMD4A, FSHR, HTR4, LIX1, LRRTM2, LTBP1, MARCKS, MBD3L1, NEUROG2, NT5C1B, OR2A12, OR2A14, OR5B21, OR5H2, OR6C75, OR6K6, OR8H1, OR8S1, POU4F1, PPFIA2, RGR, SEC23B, SPECC1, SPNS3, TMED10, TMEM59, TNNI3K, TSHR, XIRP2, ZCCHC5 |
| **PSG1** | 74 | both CNV-TF regulated and CNV-genes enriched | ABLIM3, AFAP1, AGBL4, ATF2, B4GALNT3, BAG1, BRSK1, AAMDC, C17orf85, C9orf41, CALM3, CDH8, CDKL2, CHP1, CKAP4, CKS1B, CORO1C, CSNK1G1, DDIT4L, DENR, DNAH9, DPP6, EIF3H, ERGIC3, FAM49A, FBXO47, FGF14, FKBP8, FOXI2, GABRQ, GJB6, GNL2, GPC5, HOXA4, KCNA1, KCNE1L, KCNG3, KDM6A, KIRREL2, LIMK2, LIN28A, LRRC37A2, MAP2K4, MAP3K7, MPPED2, MTCH2, NEUROD2, NEUROG1, PHF12, PIP5K1B, PON1, PPIP5K1, PTPN14, PTPN7, REM1, RNF165, RUNDC3B, SEMA3B, SEZ6, SLC25A40, SNX14, SOX11, SSX2IP, STAT3, THRA, THSD1, TIPRL, TP53BP2, TRAF3, TSC22D3, UBA1, USP19, ZDHHC15, ZWILCH |
| **SP1** | 172 | CNV-genes-only enriched | ABCC4, ADSS, AGXT2L2, AKAP1, AKT2, ANAPC16, ARGLU1, ARHGAP1, ATP11B, ATP6V0B, ATP6V1A, ATP8B1, B3GNT2, BAZ1B, BCL3, BEX2, C12orf52, C17orf49, TMEM256, C1orf95, CCNI, CCNT2, CD47, CDK2, CEP95, CHKB, CLDND1, CLEC16A, CLIP1, CLOCK, CPT2, CRIP3, CS, CXXC5, DAAM1, DBN1, DCAF7, DCBLD2, DDX42, DDX5, DERA, DYNLL2, E2F3, ECE1, EFS, EIF4E3, FAM105B, FBL, FHL3, FNBP1, GPR50, GSK3B, HCFC1, HIAT1, HNRNPUL1, HS3ST6, HSPA13, IER3IP1, IRF1, ITGB1, KCTD12, KANSL1, FAM214B, KLF15, KLF3, KLHL25, KNTC1, KRT19, LIN54, LPXN, LTBP3, MAPRE1, MECR, MEX3C, MIER1, MKRN1, MLX, MON2, MORC3, MTCH1, MTPN, MUM1, MYCBP2, NAT9, NCK1, NDE1, NDUFAB1, NOC3L, NOTCH1, NSF, NT5C3A, NUDT3, NUFIP2, OSBP, OXR1, PAX2, PCIF1, PDHB, PHF15, PHLDB2, PIK3R2, PLEC, PLSCR3, PPP1R12A, PPP1R9A, PSMC6, PTMA, R3HDM1, RAB11A, RAB2B, RAB35, RAB8B, RABGGTA, RAD23A, RASSF3, RBM14, RBM47, RNF144A, RNF41, RNGTT, ROCK2, RUFY3, S100A13, SCAF1, SCAMP2, 2-Sep, SERTAD2, SFI1, SHKBP1, SIX5, SKI, SLC29A1, SLC43A2, SMARCC1, SMARCE1, SMTN, SNX11, SP4, SPAST, SPECC1L, SPIN4, SRPK1, SRSF2, ST13, STIL, STK3, STK4, STX1A, TADA1, TGIF1, TMEM110, TMEM132E, TMEM59, TRAPPC3, TRIM33, TRIM44, TRIM47, TSC22D2, UBAP2L, UBE2N, UBL3, UBP1, UBXN4, VPS35, VPS39, XPO1, YWHAG, YY1, ZDHHC5, ZFAND2B, ZMYM4, ZNF275 |
| **STAT1** | 96 | CNV-genes-only enriched | AARS, ABI3, ACAP1, ADCK4, AHDC1, AKAP10, ANKS1A, APTX, ARGLU1, ARMC5, ASPSCR1, ATG3, BTF3, SMCO4, PIANP, C19orf43, C19orf59, C1QC, C21orf67, C4A, FAM219A, CBX3, CCDC51, CENPB, CFD, CHRD, COG3, COMMD5, CXCL9, CYP1B1, DAB2, DCTN5, DEF6, DHRS4, DHX40, DPAGT1, EMILIN1, ENSA, EP300, EPB41L1, EVL, FIBP, GRWD1, GSDMD, H3F3A, HARS, IFIT3, IL18BP, ITPRIPL2, KAT6B, KIAA1328, KIF4A, KLHDC3, KLHDC8B, KRT39, LGALS9, LPP, MRPL34, NDUFA1, NDUFA12, NEDD8, NR1H3, PARP10, PDE12, PHF6, PI4K2A, PIGU, PNPLA6, PPAPDC2, PPIF, PSMB1, PSMB8, PTMS, PTPN6, RAP1GAP, RAPGEF6, RBM42, RFC1, RHOC, RPL18A, RSAD2, SETD3, SLC30A7, SLCO2A1, SNORA25, STEAP3, STX12, TMUB2, TRABD, UBA7, UBE2M, ZBTB4, ZNF2, ZNF570, ZNF580, ZNF787 |
| **STAT5B** | 83 | CNV-genes-only enriched | ABCF3, ABTB2, ARL6IP1, ATPIF1, BCL9, PLEKHS1, C19orf43, CBX5, CDC37L1, CDC45, CEBPZ, CFL1, COX5B, DDX6, DHX40, DPYSL2, DTX2, EIF4E, ENY2, ERP44, FAM98A, FITM2, GABPB1, GPR108, HARS, HARS2, KIAA1598, LY6H, MBD1, MCAM, MCFD2, MED1, METTL15, MON1B, MTMR4, NFKBIB, NR1H3, NUP205, OR4D6, OSM, PANK1, PDE11A, PIGV, PRKCH, PSRC1, RANGRF, RAP2C, RBKS, RNF214, RPS6KA3, RRP8, S100PBP, SETD3, SLC30A7, SLC35F1, SYF2, SYNCRIP, SYNPR, TAF1B, TAF9, TCEB1, TCERG1, TUBE1, U2AF2, WDR81, XIRP2, ZFR, ZMAT2, ZNF410, ZNF622, ZNF777, ZNF787, ZYX, CWC22, ABHD17B, KCTD1, KRTAP13-3, LIMS1, MIDN, SLC25A37, SPRY2, TLR6, YTHDF2 |
| **STAT3** | 82 | CNV-genes-only enriched | AARS, ABCC4, AKT2, AMFR, APTX, ARIH2, ASB6, ASTE1, ASXL2, ATP6V0D1, ATP7B, BMF, BTF3, BTG2, C1QC, AAR2, C6orf62, CALM3, CD40LG, CDKL5, CDKN2B, CMAS, CRK, CRLS1, CSTF3, CTLA4, DCTN5, DUS2L, DYNC1I2, FIBP, FOS, GART, GNB2L1, KPNA2, KRT15, LOC595101, LPP, METTL7A, MORN1, MRPL10, MXI1, NBEA, NDUFA1, NDUFA12, NIP7, NPAT, NR3C1, OR2A20P, OR51B6, PDE4D, PDF, PDS5A, PEX13, PGM1, PKDCC, POLA1, PPIE, PSMB5, RPS19, SDCCAG3, SETD8, SLC35B4, SMARCA1, SPNS1, STEAP4, TBX2, TEX10, TMEM187, TOMM70A, TPT1, TRIP12, TTC3, TTLL5, TUBGCP2, VCPIP1, ZBTB41, ZFAND5, ZNF148, ZNF444, ZNF689, ZNF829, ZSCAN12 |
| **TBP** | 25 | CNV-TF-only regulated | AARSD1, ANP32A, ARHGEF12, ARL6IP1, C1orf43, CALM1, CASP2, CREB3L2, FIZ1, GCLC, HES1, HIST1H2BB, HIST3H3, HMGN2, HSPA2, HSPA8, KPNA2, OAZ1, PGBD1, RPS20, SVIL, TAX1BP3, UGDH, YOD1, ZMYND8 |
| **USF1** | 54 | CNV-genes-only enriched | AKAP1, ARF6, ARSA, ATG3, ATP6V0B, CALR, CIC, CROCC, DNAJC11, FAM179B, FBXO33, GABARAP, GATSL3, H2AFZ, HIST3H2BB, HNRNPA3, HOXD9, HSPA9, KIF9, LAPTM4A, MAN2A2, MARS2, MYF5, NPAT, NR3C1, PBXIP1, PDE2A, PFDN6, PI4KA, PKN1, PLBD2, POLH, PPARGC1B, PPIA, PPP2R5C, PTPRS, PTRHD1, RABEP1, RARG, RICTOR, RORC, SCFD2, SEC23IP, SLC16A13, SLC2A4, SLC9A2, SMARCC1, ST13, STT3B, TAF8, TNIP2, USP31, VPS33A, WDR81 |
| **XBP1** | 86 | CNV-genes-only enriched | AHCYL1, AKAP1, ARC, BAZ2B, BMF, BRD2, C5orf51, CCBE1, CD164, CDK2, COPA, COPG1, DDX42, ENTPD4, ENTPD7, EPM2AIP1, GOLPH3, HCFC1, HMGB2, HSPBAP1, IP6K1, KIAA0895L, MON2, NDEL1, NEUROG3, ORC4, PGAP2, PI4KA, PITX2, PLA2G12A, POLE4, PPAP2A, PRELID1, RPS13, RSPH9, SEC61B, SLC25A16, SLC25A46, SLC33A1, SLC36A1, SLC38A10, SLCO3A1, SREK1, SRSF2, TCEAL8, TCF4, TMED9, TMPO, UBXN4, XPO4, YWHAG, ZC3HAV1L, ACTR3, AGPS, AKAP4, BCL11B, GID8, C7orf43, C8orf58, C9orf163, FAM219A, CIT, DCAF6, DNAJA1, FAM120A, FBXO11, FKBP11, GNA13, KCTD12, KLHDC2, KLHL38, MBNL2, MITF, NRG1, NSD1, NUCB2, OR51G1, PKP1, PORCN, PTGES3, RALGAPA1, SCFD1, SERINC4, SLC22A6, SNHG10, TRIM58 |
| **YY1** | 91 | both CNV-TF regulated and CNV-genes enriched | ACTR3, ADAM15, ALDH9A1, APPBP2, ARGLU1, ARHGAP36, ATF6, ATP5B, ATP5G1, ATP6V1A, BRCA1, BROX, C12orf52, MGME1, CHD2, CNOT1, COPA, COX4I1, DAAM1, DCUN1D4, DTWD2, E2F3, FAM120A, FHIT, GNB1, H3F3AP4, HARS2, HNRNPA1P10, HNRNPD, HNRNPR, IFITM3, IKZF5, IL10, IST1, ITCH, GLTSCR1L, KANSL1, KIF5B, LSM1, MAPKAPK3, MGAT4B, MPZL2, MRPL47, MTCH1, MYH10, NACA, NACC2, NCL, NDE1, OXSR1, PAPOLA, PARD6G, PHF5A, PPP1R15B, PPP1R21, PPP2R2A, PRDX6, PSMA4, PTPLAD2, RAB10, RAB2B, RABAC1, RAD21, RAN, RBM12, RHOA, RPS27A, SCAF1, SEPHS2, 2-Sep, 9-Sep, SKIV2L2, SQLE, STK3, SWAP70, SYNCRIP, TBC1D13, TMED10, TPM3, TSC22D2, TXNDC17, UBE2Q2, UBXN4, WDR33, XPO1, YES1, YIPF3, YWHAG, ZFP90, ZNF207, ZNF362 |
| **ZSCAN1** | 123 | both CNV-TF regulated and CNV-genes enriched | ADAMTS8, AEBP2, AMOTL2, AP2M1, APBB1, ARAP1, ARF3, ARFIP2, ARHGEF25, ARL8A, BACE1, BAHD1, BATF3, BLMH, BMPR1A, KANSL1L, C3orf17, C6orf223, CCDC142, CD83, CFTR, CLMP, CPEB3, DLL4, DZIP1, EGFL8, EMX2, ENOX1, EPB41, ERI3, FAM163B, FAM63A, FKBP10, FOXO3, FOXP2, GDF11, GNB4, GRIN2A, HIVEP2, HMHA1, HN1, HPS5, IGF2-AS, IL17D, INSRR, KAT5, KCND1, KCNN4, KCTD1, KDM4A, KLF7, KLHL18, KRCC1, MAN2A1, MAST2, MCAM, MDFI, MED1, MEX3B, MGAT1, MXI1, NARS2, NBL1, NEK6, NOSIP, NRCAM, NUTF2, PBX1, PDE1B, PDE4A, PDGFC, PFKP, PIAS3, PIP5K1A, POLE, PPIG, PROCA1, PRR3, PTGER2, PTPN5, PTPN9, QSER1, RAB3A, RIC8B, RNF5, RPL21, RTN4, SDC1, SENP3, SHISA4, SLC22A17, SLC26A2, SMAD3, SOAT1, SPSB4, SRCAP, SREBF2, STAG2, SWT1, TAB2, TAOK1, TCEAL5, TGFBR1, TMEFF1, TMEM109, TMEM8B, TNFSF11, TOB2, TOP1, TRIM41, TRPC5, TSNAXIP1, UBE2Q2P1, UBE2R2, UNC80, USF2, VAMP1, WDR13, WDTC1, WNT9B, XIAP, ZBTB8OS, ZDHHC2 |

**Full list of 33 regulatory modules of CNV-ICC-TRN.**
