# Supplementary material for: Integrative Analysis of Transcriptional Regulatory Network and Copy Number Variation in Intrahepatic Cholangiocarcinoma
Source: PLoS One. 2014 Jun 4;9(6):e98653. doi: 10.1371/journal.pone.0098653 (PMC4045758; doi:10.1371/journal.pone.0098653)
Supplement: Table S3 — Enriched KEGG signaling pathways of 33 regulatory modules in CNV-ICC-TRN. Enrichment analysis was performed using one-side Fisher's exact test, and significance threshold was FDR<0.05. Modules are represented by their regulators' names. (DOC) [file pone.0098653.s004.doc]

**SI-Table 3.**

| **Module** | **Description** | | **Genes in Gene Set (K)** | **Genes in Overlap (k)** | | **p-value** | | **FDR** | |  | |
| --- | --- | --- | --- | --- | --- | --- | --- | --- | --- | --- | --- |
| **YY1** | Oxidative phosphorylation~0190 | | 132 | 4 | | 0.0057 | | 0.0285 | |  | |
| **DAND5** | Purine metabolism~0230 | | 162 | 5 | | 0.0159 | | 0.0455 | |  | |
| **STAT3** | Purine metabolism~0230 | | 162 | 4 | | 0.0080 | | 0.0314 | |  | |
| **PAX5** | Inositol phosphate metabolism~0562 | | 57 | 4 | | 0.0020 | | 0.0185 | |  | |
| **XBP1** | Ether lipid metabolism~0565 | | 36 | 3 | | 0.0010 | | 0.0128 | |  | |
| **MYCN** | Ribosome biogenesis in eukaryotes~3008 | | 81 | 4 | | 0.0002 | | 0.0107 | |  | |
| **MYCN** | RNA transport~3013 | | 152 | 4 | | 0.0024 | | 0.0191 | |  | |
| **MZF1** | RNA transport~3013 | | 152 | 5 | | 0.0144 | | 0.0430 | |  | |
| **MZF1** | mRNA surveillance pathway~3015 | | 83 | 5 | | 0.0012 | | 0.0144 | |  | |
| **YY1** | RNA degradation~3018 | | 71 | 3 | | 0.0069 | | 0.0311 | |  | |
| **STAT5B** | Spliceosome~3040 | | 128 | 4 | | 0.0037 | | 0.0233 | |  | |
| **CREB1** | Nucleotide excision repair~3420 | | 45 | 4 | | 0.0017 | | 0.0179 | |  | |
| **NFKB2** | MAPK signaling pathway~4010 | | 268 | 3 | | 0.0155 | | 0.0455 | |  | |
| **PSG1** | MAPK signaling pathway~4010 | | 268 | 6 | | 0.0011 | | 0.0139 | |  | |
| **ELK1** | ErbB signaling pathway~4012 | | 87 | 4 | | 0.0020 | | 0.0185 | |  | |
| **SP1** | ErbB signaling pathway~4012 | | 87 | 4 | | 0.0124 | | 0.0395 | |  | |
| **CREB1** | Cell cycle~4110 | | 128 | 5 | | 0.0129 | | 0.0404 | |  | |
| **AHR** | Oocyte meiosis~4144 | | 114 | 4 | | 0.0165 | | 0.0455 | |  | |
| **NFYB** | Oocyte meiosis~4144 | | 114 | 3 | | 0.0107 | | 0.0368 | |  | |
| **AHR** | Ubiquitin mediated proteolysis~4120 | | 139 | 5 | | 0.0069 | | 0.0311 | |  | |
| **HNF4A** | Ubiquitin mediated proteolysis~4120 | | 139 | 3 | | 0.0165 | | 0.0455 | |  | |
| **PAX5** | Ubiquitin mediated proteolysis~4120 | | 139 | 7 | | 0.0003 | | 0.0107 | |  | |
| **AHR** | Protein processing in endoplasmic reticulum~4141 | | 168 | 5 | | 0.0144 | | 0.0430 | |  | |
| **TBP** | Protein processing in endoplasmic reticulum~4141 | | 168 | 3 | | 0.0020 | | 0.0185 | |  | |
| **USF1** | Lysosome~4142 | | 121 | 3 | | 0.0070 | | 0.0311 | |  | |
| **ZSCAN1** | Endocytosis~4144 | | 203 | 5 | | 0.0161 | | 0.0455 | |  | |
| **TFAP2B** | Cardiac muscle contraction~4260 | | 77 | 3 | | 0.0053 | | 0.0270 | |  | |
| **AHR** | Wnt signaling pathway~4310 | | 151 | 5 | | 0.0095 | | 0.0343 | |  | |
| **TFAP2A** | Wnt signaling pathway~4310 | | 151 | 4 | | 0.0087 | | 0.0329 | |  | |
| **NFKB2** | Wnt signaling pathway~4310 | | 151 | 3 | | 0.0033 | | 0.0220 | |  | |
| **PAX5** | Wnt signaling pathway~4310 | | 151 | 5 | | 0.0121 | | 0.0388 | |  | |
| **TFAP2A** | TGF-beta signaling pathway~4350 | | 85 | 5 | | 0.0001 | | 0.0091 | |  | |
| **PAX5** | TGF-beta signaling pathway~4350 | | 85 | 5 | | 0.0012 | | 0.0139 | |  | |
| **MZF1** | Axon guidance~4360 | | 130 | 5 | | 0.0079 | | 0.0314 | |  | |
| **PSG1** | Axon guidance~4360 | | 130 | 4 | | 0.0026 | | 0.0191 | |  | |
| **AHR** | VEGF signaling pathway~4370 | | 76 | 4 | | 0.0043 | | 0.0249 | |  | |
| **E2F1** | Osteoclast differentiation~4380 | | 128 | 4 | | 0.0163 | | 0.0455 | |  | |
| **NFKB2** | Osteoclast differentiation~4380 | | 128 | 3 | | 0.0021 | | 0.0185 | |  | |
| **BACH2** | Focal adhesion~4510 | | 200 | 3 | | 0.0030 | | 0.0204 | |  | |
| **PATZ1** | Focal adhesion~4510 | | 200 | 5 | | 0.0047 | | 0.0252 | |  | |
| **SP1** | Focal adhesion~4510 | | 200 | 6 | | 0.0163 | | 0.0455 | |  | |
| **DAND5** | Tight junction~4530 | | 133 | 5 | | 0.0074 | | 0.0314 | |  | |
| **NFYB** | Tight junction~4530 | | 133 | 3 | | 0.0159 | | 0.0455 | |  | |
| **YY1** | Tight junction~4530 | | 133 | 5 | | 0.0008 | | 0.0128 | |  | |
| **ARNT** | Gap junction~4540 | | 90 | 4 | | 0.0149 | | 0.0442 | |  | |
| **CEBPA** | Complement and coagulation cascades~4610 | | 69 | 3 | | 0.0038 | | 0.0233 | |  | |
| **STAT1** | Complement and coagulation cascades~4610 | | 69 | 3 | | 0.0074 | | 0.0314 | |  | |
| **E2F1** | Toll-like receptor signaling pathway~4620 | | 102 | 4 | | 0.0078 | | 0.0314 | |  | |
| **PSG1** | Toll-like receptor signaling pathway~4620 | | 102 | 3 | | 0.0104 | | 0.0361 | |  | |
| **TFAP2A** | Jak-STAT signaling pathway~4630 | | 155 | 4 | | 0.0095 | | 0.0343 | |  | |
| **E2F1** | T cell receptor signaling pathway~4660 | | 108 | 5 | | 0.0015 | | 0.0159 | |  | |
| **PAX5** | T cell receptor signaling pathway~4660 | | 108 | 4 | | 0.0170 | | 0.0460 | |  | |
| **STAT3** | T cell receptor signaling pathway~4660 | | 108 | 4 | | 0.0020 | | 0.0185 | |  | |
| **PATZ1** | Leukocyte transendothelial migration~4670 | | 117 | 4 | | 0.0039 | | 0.0233 | |  | |
| **AHR** | Neurotrophin signaling pathway~4722 | | 127 | 5 | | 0.0048 | | 0.0252 | |  | |
| **PAX5** | Neurotrophin signaling pathway~4722 | | 127 | 6 | | 0.0011 | | 0.0139 | |  | |
| **ARNT** | Long-term depression~4730 | | 70 | 4 | | 0.0066 | | 0.0311 | |  | |
| **POU3F2** | Olfactory transduction~4740 | | 388 | 8 | | 0.0000 | | 0.0015 | |  | |
| **DAND5** | Regulation of actin cytoskeleton~4810 | | 214 | 7 | | 0.0034 | | 0.0220 | |  | |
| **PATZ1** | Regulation of actin cytoskeleton~4810 | | 214 | 7 | | 0.0002 | | 0.0091 | |  | |
| **ELK1** | Insulin signaling pathway~4910 | | 138 | 4 | | 0.0098 | | 0.0351 | |  | |
| **RFX1** | Insulin signaling pathway~4910 | | 138 | 5 | | 0.0010 | | 0.0128 | |  | |
| **HNF4A** | GnRH signaling pathway~4912 | | 101 | 3 | | 0.0071 | | 0.0311 | |  | |
| **PATZ1** | Melanogenesis~4916 | | 101 | 3 | | 0.0179 | | 0.0481 | |  | |
| **HNF4A** | Maturity onset diabetes of the young~4950 | | 25 | 4 | | 0.0000 | | 0.0015 | |  | |
| **AHR** | Vasopressin-regulated water reabsorption~4962 | | 44 | 3 | | 0.0068 | | 0.0311 | |  | |
| **DAND5** | Vasopressin-regulated water reabsorption~4962 | | 44 | 3 | | 0.0081 | | 0.0314 | |  | |
| **PATZ1** | Vasopressin-regulated water reabsorption~4962 | | 44 | 3 | | 0.0020 | | 0.0185 | |  | |
| **SP1** | Vasopressin-regulated water reabsorption~4962 | | 44 | 3 | | 0.0110 | | 0.0373 | |  | |
| **HNF4A** | Bile secretion~4976 | | 71 | 3 | | 0.0027 | | 0.0197 | |  | |
| **YY1** | Alzheimer's disease~5010 | | 168 | 4 | | 0.0128 | | 0.0403 | |  | |
| **AHR** | Amyotrophic lateral sclerosis (ALS)~5014 | | 54 | 3 | | 0.0116 | | 0.0383 | |  | |
| **TFAP2A** | Bacterial invasion of epithelial cells~5100 | | 71 | 3 | | 0.0067 | | 0.0311 | |  | |
| **CREB1** | Bacterial invasion of epithelial cells~5100 | | 71 | 4 | | 0.0079 | | 0.0314 | |  | |
| **TFAP2A** | Epithelial cell signaling in Helicobacter pylori infection~5120 | | 68 | 3 | | 0.0060 | | 0.0296 | |  | |
| **TFAP2A** | Shigellosis~5131 | | 62 | 3 | | 0.0047 | | 0.0252 | |  | |
| **STAT3** | Chagas disease (American trypanosomiasis)~5142 | | 104 | 3 | | 0.0143 | | 0.0430 | |  | |
| **NFKB2** | Toxoplasmosis~5145 | | 133 | 3 | | 0.0023 | | 0.0191 | |  | |
| **STAT1** | Staphylococcus aureus infection~5150 | | 56 | 3 | | 0.0043 | | 0.0249 | |  | |
| **AHR** | Pathways in cancer~5200 | | 327 | 9 | | 0.0019 | | 0.0185 | |  | |
| **BAX** | Pathways in cancer~5200 | | 327 | 4 | | 0.0001 | | 0.0091 | |  | |
| **E2F1** | Pathways in cancer~5200 | | 327 | 7 | | 0.0118 | | 0.0383 | |  | |
| **PATZ1** | Pathways in cancer~5200 | | 327 | 6 | | 0.0086 | | 0.0329 | |  | |
| **E2F1** | | Pancreaticcancer~5212 | | | 70 | | 3 | | 0.0166 | | 0.0455 |
| **CREB1** | Endometrial cancer~5213 | | 52 | 4 | | 0.0028 | | 0.0197 | |  | |
| **SP1** | Endometrial cancer~5213 | | 52 | 3 | | 0.0169 | | 0.0459 | |  | |
| **E2F1** | Glioma~5214 | | 65 | 3 | | 0.0137 | | 0.0423 | |  | |
| **CREB1** | Prostate cancer~5215 | | 89 | 4 | | 0.0164 | | 0.0455 | |  | |
| **E2F1** | Prostate cancer~5215 | | 89 | 4 | | 0.0049 | | 0.0257 | |  | |
| **MZF1** | Prostate cancer~5215 | | 89 | 4 | | 0.0103 | | 0.0361 | |  | |
| **SP1** | Prostate cancer~5215 | | 89 | 5 | | 0.0023 | | 0.0191 | |  | |
| **MAX** | Basal cell carcinoma~5217 | | 55 | 3 | | 0.0075 | | 0.0314 | |  | |
| **E2F1** | Melanoma~5218 | | 71 | 3 | | 0.0172 | | 0.0464 | |  | |
| **TFAP2A** | Chronic myeloid leukemia~5220 | | 73 | 4 | | 0.0007 | | 0.0128 | |  | |
| **E2F1** | Chronic myeloid leukemia~5220 | | 73 | 3 | | 0.0185 | | 0.0492 | |  | |
| **AHR** | Acute myeloid leukemia~5221 | | 58 | 3 | | 0.0140 | | 0.0428 | |  | |
| **E2F1** | Small cell lung cancer~5222 | | 85 | 5 | | 0.0005 | | 0.0114 | |  | |
| **PATZ1** | Small cell lung cancer~5222 | | 85 | 3 | | 0.0115 | | 0.0381 | |  | |
| **SP1** | Small cell lung cancer~5222 | | 85 | 5 | | 0.0019 | | 0.0185 | |  | |
| **E2F1** | Non-small cell lung cancer~5223 | | 54 | 3 | | 0.0085 | | 0.0328 | |  | |
| **SP1** | Non-small cell lung cancer~5223 | | 54 | 4 | | 0.0025 | | 0.0191 | |  | |
| **ESR1** | Systemic lupus erythematosus~5322 | | 138 | 3 | | 0.0068 | | 0.0311 | |  | |
| **NFIC** | Systemic lupus erythematosus~5322 | | 138 | 4 | | 0.0009 | | 0.0128 | |  | |
| **STAT3** | Rheumatoid arthritis~5323 | | 92 | 3 | | 0.0104 | | 0.0361 | |  | |
| **PATZ1** | Hypertrophic cardiomyopathy (HCM)~5410 | | 83 | 4 | | 0.0012 | | 0.0139 | |  | |
| **PATZ1** | Arrhythmogenic right ventricular cardiomyopathy (ARVC)~5412 | | 74 | 4 | | 0.0008 | | 0.0128 | |  | |
| **TFAP2B** | Dilated cardiomyopathy~5414 | | 90 | 3 | | 0.0080 | | 0.0314 | |  | |
| **DAND5** | Dilated cardiomyopathy~5414 | | 90 | 4 | | 0.0094 | | 0.0343 | |  | |
| **PATZ1** | Dilated cardiomyopathy~5414 | | 90 | 5 | | 0.0002 | | 0.0091 | |  | |

**Enriched KEGG signaling pathways of 33 regulatory modules in CNV-ICC-TRN.**

Enrichment analysis was performed using one-side Fisher’s exact test, and significance threshold was FDR<0.05. Modules are represented by their regulators’ names.
