# Supplementary material for: Integrative Analysis of Transcriptional Regulatory Network and Copy Number Variation in Intrahepatic Cholangiocarcinoma
Source: PLoS One. 2014 Jun 4;9(6):e98653. doi: 10.1371/journal.pone.0098653 (PMC4045758; doi:10.1371/journal.pone.0098653)
Supplement: Table S4 — Samples distribution between ICC classes. Samples distribution of our clustering result and Sia's clustering result. (DOC) [file pone.0098653.s005.doc]

**SI-Table 4.**

| **Samples** | **Our classification** | **Sia el al classification** |
| --- | --- | --- |
| **CCM022** | clusster I | Inflammation |
| **CCM002** | clusster I | Inflammation |
| **CCBCN16** | clusster I | Inflammation |
| **CCBCN21** | clusster I | Inflammation |
| **CCM019** | clusster I | Inflammation |
| **CCM013** | clusster I | Inflammation |
| **CCM008** | clusster I | Inflammation |
| **CCM017** | clusster I | Inflammation |
| **CCM016** | clusster I | Inflammation |
| **CCM018** | clusster I | Inflammation |
| **CCM056** | clusster I | Inflammation |
| **CCNY051** | clusster I | Inflammation |
| **CCM026** | clusster I | Inflammation |
| **CCM042** | clusster I | Inflammation |
| **CCM025** | clusster I | Inflammation |
| **CCM030** | clusster I | Inflammation |
| **CCM044** | clusster I | Inflammation |
| **CCBCN22** | clusster I | Inflammation |
| **CCM071** | clusster I | Inflammation |
| **CCBCN013** | clusster I | Inflammation |
| **CCBCN006** | clusster I | Inflammation |
| **CCBCN011** | clusster I | Inflammation |
| **CCM062** | clusster I | Inflammation |
| **CCM063** | clusster I | Inflammation |
| **CCM064** | clusster I | Inflammation |
| **CCM065** | clusster I | Inflammation |
| **CCM061** | clusster I | Inflammation |
| **CCM070** | clusster I | Inflammation |
| **CCM038** | clusster I | Inflammation |
| **CCM043** | clusster I | Inflammation |
| **CCM027** | clusster I | Inflammation |
| **CCM006** | clusster I | Inflammation |
| **CCM007** | clusster I | Inflammation |
| **CCM005** | clusster I | Inflammation |
| **CCM012** | clusster I | Inflammation |
| **CCM014** | clusster I | Inflammation |
| **CCM029** | clusster I | Inflammation |
| **CCNY012** | clusster I | Inflammation |
| **CCM039** | clusster I | Inflammation |
| **CCM041** | clusster I | Inflammation |
| **CCBCN26** | clusster I | Proliferation |
| **CCM037** | clusster P | Proliferation |
| **CCM020** | clusster P | Proliferation |
| **CCM053** | clusster P | Proliferation |
| **CCM050** | clusster P | Proliferation |
| **CCM058** | clusster P | Proliferation |
| **CCM060** | clusster P | Proliferation |
| **CCBCN24** | clusster P | Proliferation |
| **CCNY005** | clusster P | Proliferation |
| **CCBCN27** | clusster P | Proliferation |
| **CCNY19** | clusster P | Proliferation |
| **CCNY014** | clusster P | Proliferation |
| **CCBCN25** | clusster P | Proliferation |
| **CCM057** | clusster P | Proliferation |
| **CCNY045** | clusster P | Proliferation |
| **CCNY21** | clusster P | Proliferation |
| **CCM031** | clusster P | Proliferation |
| **CCM034** | clusster P | Proliferation |
| **CCNY001** | clusster P | Proliferation |
| **CCNY011** | clusster P | Proliferation |
| **CCM032** | clusster P | Proliferation |
| **CCM033** | clusster P | Proliferation |
| **CCM049** | clusster P | Proliferation |
| **CCM045** | clusster P | Proliferation |
| **CCM046** | clusster P | Proliferation |
| **CCM055** | clusster P | Proliferation |
| **CCM052** | clusster P | Proliferation |
| **CCM054** | clusster P | Proliferation |
| **CCM047** | clusster P | Proliferation |
| **CCM051** | clusster P | Proliferation |
| **CCNY007** | clusster P | Proliferation |
| **CCNY015** | clusster P | Proliferation |
| **CCNY032** | clusster P | Proliferation |
| **CCNY035** | clusster P | Proliferation |
| **CCM040** | clusster P | Proliferation |
| **CCNY048** | clusster P | Proliferation |
| **CCNY047** | clusster P | Proliferation |
| **CCNY054** | clusster P | Proliferation |
| **CCNY038** | clusster P | Proliferation |
| **CCNY039** | clusster P | Proliferation |
| **CCNY044** | clusster P | Proliferation |
| **CCNY042** | clusster P | Proliferation |
| **CCNY057** | clusster P | Proliferation |
| **CCNY061** | clusster P | Proliferation |
| **CCNY062** | clusster P | Proliferation |
| **CCNY052** | clusster P | Proliferation |
| **CCNY049** | clusster P | Proliferation |
| **CCNY046** | clusster P | Proliferation |
| **CCNY060** | clusster P | Proliferation |
| **CCNY059** | clusster P | Proliferation |
| **CCNY064** | clusster P | Proliferation |
| **CCNY18** | clusster P | Proliferation |
| **CCNY031** | clusster P | Proliferation |
| **CCM003** | clusster P | Proliferation |
| **CCM036** | clusster P | Proliferation |
| **CCNY037** | clusster P | Proliferation |
| **CCNY053** | clusster P | Proliferation |
| **CCNY063** | clusster P | Proliferation |
| **CCNY058** | clusster P | Proliferation |
| **CCNY029** | clusster P | Proliferation |
| **CCNY17** | clusster P | Proliferation |
| **CCNY010** | clusster P | Proliferation |
| **CCNY028** | clusster P | Proliferation |
| **CCNY002** | clusster P | Proliferation |
| **CCNY009** | clusster P | Proliferation |
| **CCNY033** | clusster P | Proliferation |
| **CCNY043** | clusster P | Proliferation |
| **CCNY004** | clusster P | Proliferation |
| **CCNY041** | clusster P | Proliferation |
| **CCNY055** | clusster P | Proliferation |
| **CCNY040** | clusster P | Proliferation |
| **CCNY013** | clusster P | Proliferation |
| **CCM059** | clusster I | Proliferation |
| **CCBCN004** | clusster I | Proliferation |
| **CCNY20** | clusster I | Proliferation |
| **CCM048** | clusster I | Proliferation |
| **CCNY034** | clusster I | Proliferation |
| **CCBCN005** | clusster I | Proliferation |
| **CCM004** | clusster I | Proliferation |
| **CCBCN002** | clusster I | Proliferation |
| **CCBCN003** | clusster I | Proliferation |
| **CCM072** | clusster I | Proliferation |
| **CCM021** | clusster I | Proliferation |
| **CCNY016** | clusster I | Proliferation |
| **CCM023** | clusster I | Proliferation |

**Samples distribution between ICC classes.**

Samples distribution among our classification result and Sia et al classification result.
