# Supplementary material for: Integrative Analysis of Transcriptional Regulatory Network and Copy Number Variation in Intrahepatic Cholangiocarcinoma
Source: PLoS One. 2014 Jun 4;9(6):e98653. doi: 10.1371/journal.pone.0098653 (PMC4045758; doi:10.1371/journal.pone.0098653)
Supplement: Table S5 — Leave-one-out cross validation of ICC classes. Modules are represented by their regulators' names. (DOC) [file pone.0098653.s006.doc]

**SI-Table 5**

| **Methods** | **#Correct** | **#Errors** | **Accuracy** |
| --- | --- | --- | --- |
| **CARTXValidation** | 111 | 14 | 89% |
| **KNNXValidation** | 123 | 2 | 98% |
| **WeightedVotingXValidation** | 119 | 6 | 95% |

**Leave-one-out cross validation of ICC classes.**

Modules are represented by their regulators’ names.
