# Supplementary material for: Integrative Analysis of Transcriptional Regulatory Network and Copy Number Variation in Intrahepatic Cholangiocarcinoma
Source: PLoS One. 2014 Jun 4;9(6):e98653. doi: 10.1371/journal.pone.0098653 (PMC4045758; doi:10.1371/journal.pone.0098653)
Supplement: Table S6 — Differentially expressed genes from CNV-ICC-TRN between cluster I and cluster P. Differential expression analysis was tested using t-test, and significance threshold was p-value<0.001. (DOC) [file pone.0098653.s007.doc]

**SI-Table 6.**

| **Symbol** | **Name** | | **t.statistic** | **p.value** | **Class** | **Fold change** |
| --- | --- | --- | --- | --- | --- | --- |
| **AAMP** | angio-associated, migratory cell protein | | 4.557611301 | 1.48E-05 | cluster P | 1.13 |
| **ABCA1** | ATP-binding cassette, sub-family A (ABC1), member 1 | | 11.02079331 | 7.16E-20 | cluster P | 1.89 |
| **ABL1** | c-abl oncogene 1, non-receptor tyrosine kinase | | 4.203678894 | 5.12E-05 | cluster P | 1.4 |
| **ACO1** | aconitase 1, soluble | | 12.6374893 | 5.75E-24 | cluster P | 1.94 |
| **ACTB** | actin, beta | | 6.812417088 | 8.79E-10 | cluster P | 1.34 |
| **ACVR1** | activin A receptor, type I | | 9.84453959 | 3.49E-17 | cluster P | 1.88 |
| **ADAM10** | ADAM metallopeptidase domain 10 | | 8.302907692 | 1.55E-13 | cluster P | 1.3 |
| **ADCY3** | adenylate cyclase 3 | | 12.23836747 | 2.26E-22 | cluster P | 2.12 |
| **ADSS** | adenylosuccinate synthase | | 5.36131288 | 4.25E-07 | cluster P | 1.35 |
| **AHCY** | adenosylhomocysteinase | | 11.22587191 | 3.94E-20 | cluster P | 2.09 |
| **AHR** | aryl hydrocarbon receptor | | 5.185433305 | 8.61E-07 | cluster P | 1.47 |
| **ALDH9A1** | aldehyde dehydrogenase 9 family, member A1 | | 7.940095905 | 5.01E-12 | cluster P | 1.47 |
| **ARF4** | ADP-ribosylation factor 4 | | 4.398892906 | 3.27E-05 | cluster P | 1.2 |
| **RHOA** | ras homolog family member A | | 14.58161061 | 4.68E-25 | cluster P | 2.96 |
| **ARHGAP1** | Rho GTPase activating protein 1 | | 9.398759917 | 1.08E-15 | cluster P | 1.95 |
| **ASPH** | aspartate beta-hydroxylase | | 6.012411987 | 2.04E-08 | cluster P | 1.32 |
| **ATP1B1** | ATPase, Na+/K+ transporting, beta 1 polypeptide | | 7.179163372 | 5.96E-11 | cluster P | 1.8 |
| **ATP5B** | ATP synthase, H+ transporting, mitochondrial F1 complex, beta polypeptide | | 9.306495332 | 1.47E-14 | cluster P | 1.7 |
| **ATP5G1** | ATP synthase, H+ transporting, mitochondrial Fo complex, subunit C1 (subunit 9) | | 8.117890093 | 5.50E-13 | cluster P | 1.68 |
| **ATP6V1A** | ATPase, H+ transporting, lysosomal 70kDa, V1 subunit A | | 12.04600166 | 1.23E-20 | cluster P | 2.26 |
| **ATP6V0B** | ATPase, H+ transporting, lysosomal 21kDa, V0 subunit b | | 6.485298752 | 1.94E-09 | cluster P | 1.45 |
| **BCL3** | B-cell CLL/lymphoma 3 | | 9.043779697 | 4.93E-14 | cluster P | 1.95 |
| **BCL9** | B-cell CLL/lymphoma 9 | | 9.182370158 | 3.88E-14 | cluster P | 2.01 |
| **BLMH** | bleomycin hydrolase | | 3.985652184 | 0.000134016 | cluster P | 1.13 |
| **BNIP3** | BCL2/adenovirus E1B 19kDa interacting protein 3 | | 7.856247339 | 1.87E-11 | cluster P | 2.45 |
| **BRCA1** | breast cancer 1, early onset | | 9.434058921 | 1.26E-15 | cluster P | 1.46 |
| **ZFP36L1** | ZFP36 ring finger protein-like 1 | | 18.14812211 | 1.42E-36 | cluster P | 2.82 |
| **KLF9** | Kruppel-like factor 9 | | 3.497328521 | 0.00065653 | cluster P | 1.12 |
| **KLF5** | Kruppel-like factor 5 (intestinal) | | 8.79616813 | 3.97E-14 | cluster P | 1.89 |
| **PTTG1IP** | pituitary tumor-transforming 1 interacting protein | | 11.0457509 | 4.27E-20 | cluster P | 2.08 |
| **CALM1** | calmodulin 1 (phosphorylase kinase, delta) | | 7.637720179 | 5.36E-12 | cluster P | 1.6 |
| **CALM2** | calmodulin 2 (phosphorylase kinase, delta) | | 11.77331509 | 9.02E-22 | cluster P | 2.13 |
| **CAMK2D** | calcium/calmodulin-dependent protein kinase II delta | | 6.20319163 | 1.17E-08 | cluster P | 1.25 |
| **CAT** | catalase | | 11.42705486 | 2.28E-19 | cluster P | 1.82 |
| **CBLB** | Cbl proto-oncogene, E3 ubiquitin protein ligase B | | 7.959876294 | 1.05E-12 | cluster P | 1.82 |
| **SERPINH1** | serpin peptidase inhibitor, clade H (heat shock protein 47), member 1, (collagen binding protein 1) | | 6.472784632 | 2.06E-09 | cluster P | 1.74 |
| **CCNB1** | cyclin B1 | | 8.953640798 | 7.60E-15 | cluster P | 1.5 |
| **CCNT2** | cyclin T2 | | 6.573305844 | 1.51E-09 | cluster P | 1.34 |
| **CD47** | CD47 molecule | | 6.109867315 | 1.22E-08 | cluster P | 1.25 |
| **CDC5L** | cell division cycle 5-like | | 8.077930211 | 8.73E-13 | cluster P | 1.63 |
| **CDK2** | cyclin-dependent kinase 2 | | 11.19409746 | 2.34E-20 | cluster P | 1.54 |
| **CEBPG** | CCAAT/enhancer binding protein (C/EBP), gamma | | 9.510342306 | 2.90E-16 | cluster P | 1.4 |
| **CENPB** | centromere protein B, 80kDa | | 6.148698433 | 2.56E-08 | cluster P | 1.33 |
| **CFL1** | cofilin 1 (non-muscle) | | 8.599596135 | 3.92E-13 | cluster P | 1.53 |
| **CHD2** | chromodomain helicase DNA binding protein 2 | | 11.13927495 | 2.43E-20 | cluster P | 1.78 |
| **CHD4** | chromodomain helicase DNA binding protein 4 | | 9.734070695 | 5.99E-17 | cluster P | 2.07 |
| **AP1S1** | adaptor-related protein complex 1, sigma 1 subunit | | 12.78084054 | 8.77E-24 | cluster P | 1.93 |
| **CLN3** | ceroid-lipofuscinosis, neuronal 3 | | 5.932142647 | 4.22E-08 | cluster P | 1.46 |
| **CNN3** | calponin 3, acidic | | 11.58803968 | 5.63E-21 | cluster P | 2.28 |
| **COL5A1** | collagen, type V, alpha 1 | | 10.27390801 | 5.43E-18 | cluster P | 1.6 |
| **COMT** | catechol-O-methyltransferase | | 7.470096669 | 1.31E-11 | cluster P | 1.62 |
| **COPA** | coatomer protein complex, subunit alpha | | 9.092017147 | 7.73E-15 | cluster P | 1.84 |
| **COX4I1** | cytochrome c oxidase subunit IV isoform 1 | | 7.902439529 | 2.07E-11 | cluster P | 1.39 |
| **COX5B** | cytochrome c oxidase subunit Vb | | 3.603981717 | 0.000519904 | cluster P | 1.18 |
| **COX11** | cytochrome c oxidase assembly homolog 11 (yeast) | | 5.868903968 | 3.86E-08 | cluster P | 1.27 |
| **CPT2** | carnitine palmitoyltransferase 2 | | 8.826675073 | 1.92E-14 | cluster P | 1.38 |
| **CREBBP** | CREB binding protein | | 7.70503251 | 3.86E-12 | cluster P | 1.35 |
| **CRIP1** | cysteine-rich protein 1 (intestinal) | | 12.24471905 | 6.85E-23 | cluster P | 2.18 |
| **CRY1** | cryptochrome 1 (photolyase-like) | | 16.14692682 | 3.58E-32 | cluster P | 2.4 |
| **CS** | citrate synthase | | 10.82612012 | 2.43E-18 | cluster P | 2.14 |
| **CSK** | c-src tyrosine kinase | | 4.553575291 | 1.34E-05 | cluster P | 1.38 |
| **CSTF3** | cleavage stimulation factor, 3' pre-RNA, subunit 3, 77kDa | | 7.898597105 | 1.34E-12 | cluster P | 1.35 |
| **CTNNA1** | catenin (cadherin-associated protein), alpha 1, 102kDa | | 8.982656158 | 1.60E-14 | cluster P | 2.04 |
| **CYP1B1** | cytochrome P450, family 1, subfamily B, polypeptide 1 | | 5.677322162 | 9.39E-08 | cluster P | 1.24 |
| **DAB2** | Dab, mitogen-responsive phosphoprotein, homolog 2 (Drosophila) | | 3.443840803 | 0.000837016 | cluster P | 1.23 |
| **DDB1** | damage-specific DNA binding protein 1, 127kDa | | 15.12751168 | 2.07E-29 | cluster P | 2.09 |
| **DDX5** | DEAD (Asp-Glu-Ala-Asp) box helicase 5 | | 13.59332635 | 1.72E-23 | cluster P | 2.51 |
| **DDX6** | DEAD (Asp-Glu-Ala-Asp) box helicase 6 | | 9.941439476 | 2.57E-17 | cluster P | 1.79 |
| **TIMM8A** | translocase of inner mitochondrial membrane 8 homolog A (yeast) | | 4.34217623 | 3.02E-05 | cluster P | 1.25 |
| **DHFR** | dihydrofolate reductase | | 5.485860657 | 2.40E-07 | cluster P | 1.37 |
| **DMPK** | dystrophia myotonica-protein kinase | | 8.992236603 | 3.81E-15 | cluster P | 1.49 |
| **DYNC1H1** | dynein, cytoplasmic 1, heavy chain 1 | | 9.746394663 | 5.60E-17 | cluster P | 1.69 |
| **DYNC1I2** | dynein, cytoplasmic 1, intermediate chain 2 | | 6.182829584 | 1.64E-08 | cluster P | 1.2 |
| **DPYSL2** | dihydropyrimidinase-like 2 | | 10.15389143 | 5.88E-18 | cluster P | 2.19 |
| **DR1** | down-regulator of transcription 1, TBP-binding (negative cofactor 2) | | 9.815249853 | 4.04E-17 | cluster P | 1.62 |
| **E2F3** | E2F transcription factor 3 | | 12.29563265 | 2.93E-22 | cluster P | 2.29 |
| **E2F4** | E2F transcription factor 4, p107/p130-binding | | 9.865531048 | 1.58E-16 | cluster P | 1.87 |
| **EFNA1** | ephrin-A1 | | 6.980926176 | 5.48E-10 | cluster P | 1.5 |
| **EIF4E** | eukaryotic translation initiation factor 4E | | 4.611661044 | 1.03E-05 | cluster P | 1.11 |
| **EIF4G2** | eukaryotic translation initiation factor 4 gamma, 2 | | 6.684653017 | 1.57E-09 | cluster P | 1.97 |
| **ELK4** | ELK4, ETS-domain protein (SRF accessory protein 1) | | 9.704541199 | 1.13E-16 | cluster P | 1.6 |
| **EMP3** | epithelial membrane protein 3 | | 3.764025438 | 0.000257797 | cluster P | 1.26 |
| **ENPEP** | glutamyl aminopeptidase (aminopeptidase A) | | 10.6976406 | 3.75E-19 | cluster P | 1.4 |
| **SLC29A1** | solute carrier family 29 (nucleoside transporters), member 1 | | 7.783232331 | 6.36E-12 | cluster P | 1.63 |
| **EPB41L2** | erythrocyte membrane protein band 4.1-like 2 | | 8.445234597 | 1.36E-13 | cluster P | 1.25 |
| **NR2F6** | nuclear receptor subfamily 2, group F, member 6 | | 5.254357372 | 7.03E-07 | cluster P | 1.23 |
| **ERBB3** | v-erb-b2 erythroblastic leukemia viral oncogene homolog 3 (avian) | | 3.499153766 | 0.000659526 | cluster P | 1.2 |
| **FBL** | fibrillarin | | 12.18652687 | 9.59E-21 | cluster P | 1.75 |
| **ETF1** | eukaryotic translation termination factor 1 | | 11.26379623 | 3.50E-19 | cluster P | 1.54 |
| **EWSR1** | Ewing sarcoma breakpoint region 1 | | 15.83722393 | 1.13E-30 | cluster P | 2.36 |
| **F3** | coagulation factor III (thromboplastin, tissue factor) | | 4.291764488 | 4.88E-05 | cluster P | 1.72 |
| **FGB** | fibrinogen beta chain | | 3.819389633 | 0.00021124 | cluster P | 1.29 |
| **FHIT** | fragile histidine triad | | 7.560027508 | 3.64E-11 | cluster P | 1.78 |
| **FKBP2** | FK506 binding protein 2, 13kDa | | 9.516059789 | 2.47E-16 | cluster P | 1.67 |
| **FUCA1** | fucosidase, alpha-L- 1, tissue | | 5.880703818 | 4.03E-08 | cluster P | 1.27 |
| **SLC37A4** | solute carrier family 37 (glucose-6-phosphate transporter), member 4 | | 4.450065283 | 2.02E-05 | cluster P | 1.28 |
| **GATA4** | GATA binding protein 4 | | 8.426249435 | 6.91E-13 | cluster P | 2.22 |
| **GHR** | growth hormone receptor | | 7.275004164 | 1.32E-10 | cluster P | 2.52 |
| **GCLC** | glutamate-cysteine ligase, catalytic subunit | | 6.68441111 | 7.94E-10 | cluster P | 1.47 |
| **GMFB** | glia maturation factor, beta | | 11.17112078 | 2.00E-20 | cluster P | 2.27 |
| **GNB1** | guanine nucleotide binding protein (G protein), beta polypeptide 1 | | 9.048514059 | 1.02E-13 | cluster P | 1.61 |
| **GOLGA4** | golgin A4 | | 8.108436967 | 4.40E-13 | cluster P | 1.38 |
| **GSK3B** | glycogen synthase kinase 3 beta | | 8.57423019 | 3.82E-14 | cluster P | 1.63 |
| **MSH6** | mutS homolog 6 (E. coli) | | 10.74260528 | 8.99E-19 | cluster P | 2.06 |
| **GTF2H1** | general transcription factor IIH, polypeptide 1, 62kDa | | 8.203351992 | 3.10E-13 | cluster P | 1.56 |
| **H2AFZ** | H2A histone family, member Z | | 4.749939983 | 5.58E-06 | cluster P | 1.32 |
| **HIST1H2BB** | histone cluster 1, H2bb | | 4.755928053 | 6.56E-06 | cluster P | 1.36 |
| **HARS** | histidyl-tRNA synthetase | | 4.866050931 | 5.05E-06 | cluster P | 1.2 |
| **HCFC1** | host cell factor C1 (VP16-accessory protein) | | 16.14223702 | 6.45E-32 | cluster P | 2.47 |
| **HHEX** | hematopoietically expressed homeobox | | 5.696387677 | 8.54E-08 | cluster P | 1.27 |
| **HIF1A** | hypoxia inducible factor 1, alpha subunit (basic helix-loop-helix transcription factor) | | 9.961842327 | 6.84E-17 | cluster P | 1.64 |
| **HINT1** | histidine triad nucleotide binding protein 1 | | 7.075100777 | 1.04E-10 | cluster P | 1.55 |
| **HMGB1** | high mobility group box 1 | | 12.00109345 | 2.09E-21 | cluster P | 1.77 |
| **HMGB2** | high mobility group box 2 | | 9.946448274 | 1.62E-15 | cluster P | 2.26 |
| **HNRNPD** | heterogeneous nuclear ribonucleoprotein D (AU-rich element RNA binding protein 1, 37kDa) | | 8.321219694 | 6.84E-12 | cluster P | 1.44 |
| **HNRNPH2** | heterogeneous nuclear ribonucleoprotein H2 (H') | | 6.143796387 | 2.03E-08 | cluster P | 1.23 |
| **HPRT1** | hypoxanthine phosphoribosyltransferase 1 | | 8.961270807 | 1.25E-14 | cluster P | 1.77 |
| **HES1** | hairy and enhancer of split 1, (Drosophila) | | 9.362719951 | 4.90E-16 | cluster P | 1.86 |
| **DNAJA1** | DnaJ (Hsp40) homolog, subfamily A, member 1 | | 7.546594226 | 8.67E-12 | cluster P | 1.54 |
| **HSPA1A** | heat shock 70kDa protein 1A | | 6.363955277 | 3.55E-09 | cluster P | 2.5 |
| **HSPA2** | heat shock 70kDa protein 2 | | 7.556497847 | 1.06E-11 | cluster P | 1.69 |
| **HSPA8** | heat shock 70kDa protein 8 | | 3.899035698 | 0.00017923 | cluster P | 1.17 |
| **ID4** | inhibitor of DNA binding 4, dominant negative helix-loop-helix protein | | 4.761344682 | 5.79E-06 | cluster P | 1.42 |
| **IDI1** | isopentenyl-diphosphate delta isomerase 1 | | 7.265718075 | 4.69E-11 | cluster P | 1.31 |
| **IGFALS** | insulin-like growth factor binding protein, acid labile subunit | | 4.547611445 | 1.31E-05 | cluster P | 1.34 |
| **IMPA1** | inositol(myo)-1(or 4)-monophosphatase 1 | | 9.133840098 | 1.32E-14 | cluster P | 1.66 |
| **INCENP** | inner centromere protein antigens 135/155kDa | | 6.203540845 | 7.74E-09 | cluster P | 1.65 |
| **ING2** | inhibitor of growth family, member 2 | | 8.771657034 | 3.93E-14 | cluster P | 1.46 |
| **INPP5A** | inositol polyphosphate-5-phosphatase, 40kDa | | 11.00330679 | 7.43E-20 | cluster P | 1.41 |
| **IRF1** | interferon regulatory factor 1 | | 9.865488655 | 1.18E-16 | cluster P | 1.48 |
| **IRS1** | insulin receptor substrate 1 | | 6.185390661 | 8.61E-09 | cluster P | 1.47 |
| **ITGA5** | integrin, alpha 5 (fibronectin receptor, alpha polypeptide) | | 9.862814633 | 5.03E-16 | cluster P | 1.6 |
| **ITGB1** | integrin, beta 1 (fibronectin receptor, beta polypeptide, antigen CD29 includes MDF2, MSK12) | | 12.62220392 | 1.14E-23 | cluster P | 2.66 |
| **JUN** | jun proto-oncogene | | 8.323390985 | 1.40E-13 | cluster P | 2.06 |
| **KCNK1** | potassium channel, subfamily K, member 1 | | 8.239122795 | 3.96E-13 | cluster P | 1.74 |
| **KCNS3** | potassium voltage-gated channel, delayed-rectifier, subfamily S, member 3 | | 7.043800126 | 1.17E-10 | cluster P | 1.63 |
| **KIF5B** | kinesin family member 5B | | 10.67037771 | 3.80E-19 | cluster P | 1.71 |
| **KPNB1** | karyopherin (importin) beta 1 | | 13.932443 | 9.85E-27 | cluster P | 2.1 |
| **KPNA2** | karyopherin alpha 2 (RAG cohort 1, importin alpha 1) | | 4.474512893 | 1.72E-05 | cluster P | 1.16 |
| **KRT8** | keratin 8 | | 6.807384992 | 3.99E-10 | cluster P | 1.5 |
| **LAMB1** | laminin, beta 1 | | 9.410203978 | 1.47E-15 | cluster P | 1.55 |
| **LTB** | lymphotoxin beta (TNF superfamily, member 3) | | 5.605840781 | 1.30E-07 | cluster P | 1.66 |
| **LY75** | lymphocyte antigen 75 | | 8.949798019 | 4.78E-15 | cluster P | 1.51 |
| **MARCKS** | myristoylated alanine-rich protein kinase C substrate | | 12.03453972 | 7.95E-21 | cluster P | 2.54 |
| **MXD1** | MAX dimerization protein 1 | | 5.681266114 | 1.02E-07 | cluster P | 1.43 |
| **SMAD7** | SMAD family member 7 | | 7.359892405 | 3.13E-11 | cluster P | 1.35 |
| **MAT2A** | methionine adenosyltransferase II, alpha | | 8.831612229 | 1.06E-14 | cluster P | 1.83 |
| **MCM6** | minichromosome maintenance complex component 6 | | 7.476485073 | 1.29E-11 | cluster P | 1.75 |
| **MFNG** | MFNG O-fucosylpeptide 3-beta-N-acetylglucosaminyltransferase | | 7.699821137 | 4.47E-12 | cluster P | 1.43 |
| **MIF** | macrophage migration inhibitory factor (glycosylation-inhibiting factor) | | 10.63675223 | 4.01E-19 | cluster P | 2.17 |
| **MAP3K11** | mitogen-activated protein kinase kinase kinase 11 | | 6.856130228 | 4.18E-10 | cluster P | 1.75 |
| **MSN** | moesin | | 4.596368857 | 1.06E-05 | cluster P | 1.41 |
| **MTR** | 5-methyltetrahydrofolate-homocysteine methyltransferase | | 4.764140043 | 5.69E-06 | cluster P | 1.17 |
| **MYC** | v-myc myelocytomatosis viral oncogene homolog (avian) | | 3.873874326 | 0.000173535 | cluster P | 1.15 |
| **MYH9** | myosin, heavy chain 9, non-muscle | | 12.19869629 | 2.46E-22 | cluster P | 2.05 |
| **MYH10** | myosin, heavy chain 10, non-muscle | | 16.24438771 | 2.30E-32 | cluster P | 2.79 |
| **MYO1E** | myosin IE | | 7.444663436 | 2.44E-11 | cluster P | 1.32 |
| **PPP1R12A** | protein phosphatase 1, regulatory subunit 12A | | 7.100842873 | 1.27E-10 | cluster P | 1.21 |
| **NACA** | nascent polypeptide-associated complex alpha subunit | | 16.12305457 | 4.09E-32 | cluster P | 2.27 |
| **NCK1** | NCK adaptor protein 1 | | 6.292729917 | 5.41E-09 | cluster P | 1.21 |
| **NCL** | nucleolin | | 12.34320121 | 1.94E-22 | cluster P | 1.84 |
| **NDUFAB1** | NADH dehydrogenase (ubiquinone) 1, alpha/beta subcomplex, 1, 8kDa | | 9.758802457 | 5.22E-17 | cluster P | 1.6 |
| **NDUFB3** | NADH dehydrogenase (ubiquinone) 1 beta subcomplex, 3, 12kDa | | 5.319014975 | 9.15E-07 | cluster P | 1.29 |
| **NDUFB8** | NADH dehydrogenase (ubiquinone) 1 beta subcomplex, 8, 19kDa | | 10.08371587 | 1.57E-16 | cluster P | 1.68 |
| **NDUFB10** | NADH dehydrogenase (ubiquinone) 1 beta subcomplex, 10, 22kDa | | 12.35178416 | 2.77E-23 | cluster P | 1.84 |
| **NFATC2** | nuclear factor of activated T-cells, cytoplasmic, calcineurin-dependent 2 | | 3.571501975 | 0.000510841 | cluster P | 1.18 |
| **NFIA** | nuclear factor I/A | | 7.205744472 | 5.14E-11 | cluster P | 1.44 |
| **NFE2L2** | nuclear factor (erythroid-derived 2)-like 2 | | 7.174943361 | 1.74E-10 | cluster P | 1.52 |
| **NFKB1** | nuclear factor of kappa light polypeptide gene enhancer in B-cells 1 | | 10.87248191 | 1.21E-17 | cluster P | 2.31 |
| **NFKBIB** | nuclear factor of kappa light polypeptide gene enhancer in B-cells inhibitor, beta | | 4.280359823 | 4.00E-05 | cluster P | 1.13 |
| **NME1** | NME/NM23 nucleoside diphosphate kinase 1 | | 4.678094585 | 9.17E-06 | cluster P | 1.37 |
| **CNOT2** | CCR4-NOT transcription complex, subunit 2 | | 7.009880144 | 1.39E-10 | cluster P | 1.5 |
| **NOTCH1** | notch 1 | | 10.29950237 | 7.64E-18 | cluster P | 1.76 |
| **SLC11A2** | solute carrier family 11 (proton-coupled divalent metal ion transporters), member 2 | | 6.880201938 | 2.72E-10 | cluster P | 1.75 |
| **NSF** | N-ethylmaleimide-sensitive factor | | 8.754533379 | 1.55E-14 | cluster P | 1.36 |
| **NUCB2** | nucleobindin 2 | | 6.754445039 | 5.78E-10 | cluster P | 1.45 |
| **OSBP** | oxysterol binding protein | | 11.33135723 | 1.64E-20 | cluster P | 1.82 |
| **PAFAH1B2** | platelet-activating factor acetylhydrolase 1b, catalytic subunit 2 (30kDa) | | 10.53272772 | 1.60E-17 | cluster P | 1.64 |
| **PCBP2** | poly(rC) binding protein 2 | | 18.00335033 | 2.02E-35 | cluster P | 2.36 |
| **PCM1** | pericentriolar material 1 | | 11.27723954 | 1.38E-19 | cluster P | 1.61 |
| **PDHB** | pyruvate dehydrogenase (lipoamide) beta | | 10.91529748 | 1.33E-17 | cluster P | 2.33 |
| **PEX13** | peroxisomal biogenesis factor 13 | | 6.052794803 | 2.45E-08 | cluster P | 1.37 |
| **PFKFB4** | 6-phosphofructo-2-kinase/fructose-2,6-biphosphatase 4 | | 4.829276565 | 4.10E-06 | cluster P | 1.63 |
| **PHKB** | phosphorylase kinase, beta | | 6.4612376 | 2.62E-09 | cluster P | 1.26 |
| **PIK3R2** | phosphoinositide-3-kinase, regulatory subunit 2 (beta) | | 9.388256341 | 4.22E-16 | cluster P | 1.94 |
| **PLCB3** | phospholipase C, beta 3 (phosphatidylinositol-specific) | | 9.31336218 | 7.25E-16 | cluster P | 1.59 |
| **PLXNB1** | plexin B1 | | 4.00184364 | 0.000109859 | cluster P | 1.38 |
| **UBL3** | ubiquitin-like 3 | | 4.192833073 | 5.29E-05 | cluster P | 1.18 |
| **PPIC** | peptidylprolyl isomerase C (cyclophilin C) | | 12.14896576 | 4.58E-21 | cluster P | 2.72 |
| **PPM1A** | protein phosphatase, Mg2+/Mn2+ dependent, 1A | | 5.107032361 | 1.23E-06 | cluster P | 1.25 |
| **PPM1B** | protein phosphatase, Mg2+/Mn2+ dependent, 1B | | 11.03549314 | 7.75E-20 | cluster P | 2.07 |
| **PPP2R1A** | protein phosphatase 2, regulatory subunit A, alpha | | 7.83254251 | 2.02E-12 | cluster P | 1.81 |
| **PPP2R2A** | protein phosphatase 2, regulatory subunit B, alpha | | 12.50552449 | 1.60E-22 | cluster P | 2.34 |
| **PPP3CA** | protein phosphatase 3, catalytic subunit, alpha isozyme | | 7.031065337 | 1.27E-10 | cluster P | 1.32 |
| **PPP6C** | protein phosphatase 6, catalytic subunit | | 14.30510445 | 1.71E-27 | cluster P | 2.24 |
| **PRPS2** | phosphoribosyl pyrophosphate synthetase 2 | | 6.979742514 | 1.96E-10 | cluster P | 1.3 |
| **RELN** | reelin | | 3.930087967 | 0.00014114 | cluster P | 1.2 |
| **PSMA4** | proteasome (prosome, macropain) subunit, alpha type, 4 | | 11.67188438 | 2.60E-21 | cluster P | 1.58 |
| **PSMA6** | proteasome (prosome, macropain) subunit, alpha type, 6 | | 13.69332812 | 2.02E-26 | cluster P | 2.74 |
| **PSMC5** | proteasome (prosome, macropain) 26S subunit, ATPase, 5 | | 14.30710448 | 1.30E-26 | cluster P | 1.76 |
| **PSMC6** | proteasome (prosome, macropain) 26S subunit, ATPase, 6 | | 7.894809063 | 1.38E-12 | cluster P | 1.46 |
| **PTGER4** | prostaglandin E receptor 4 (subtype EP4) | | 4.848288044 | 3.72E-06 | cluster P | 1.18 |
| **TWF1** | twinfilin, actin-binding protein, homolog 1 (Drosophila) | | 11.66801459 | 3.95E-21 | cluster P | 1.67 |
| **PTMA** | prothymosin, alpha | | 11.79870767 | 1.35E-20 | cluster P | 1.94 |
| **PTMS** | parathymosin | | 8.197752256 | 4.73E-13 | cluster P | 2.03 |
| **PTPN3** | protein tyrosine phosphatase, non-receptor type 3 | | 4.890104583 | 3.31E-06 | cluster P | 1.17 |
| **PTPN4** | protein tyrosine phosphatase, non-receptor type 4 (megakaryocyte) | | 9.817973686 | 4.06E-17 | cluster P | 1.35 |
| **PTPN11** | protein tyrosine phosphatase, non-receptor type 11 | | 8.005170571 | 7.63E-13 | cluster P | 1.36 |
| **PYGL** | phosphorylase, glycogen, liver | | 3.783579056 | 0.00027139 | cluster P | 1.16 |
| **RAB6A** | RAB6A, member RAS oncogene family | | 10.76032781 | 1.96E-19 | cluster P | 1.46 |
| **RAD21** | RAD21 homolog (S. pombe) | | 11.4385898 | 1.92E-19 | cluster P | 1.82 |
| **RAD23A** | RAD23 homolog A (S. cerevisiae) | | 12.57306971 | 1.98E-22 | cluster P | 2.48 |
| **RAN** | RAN, member RAS oncogene family | | 6.825322399 | 6.24E-10 | cluster P | 1.43 |
| **RAP2A** | RAP2A, member of RAS oncogene family | | 9.229378973 | 1.97E-15 | cluster P | 1.86 |
| **RBBP4** | retinoblastoma binding protein 4 | | 10.77256049 | 1.09E-17 | cluster P | 1.96 |
| **RBBP7** | retinoblastoma binding protein 7 | | 8.320232376 | 1.71E-13 | cluster P | 1.35 |
| **RCN2** | reticulocalbin 2, EF-hand calcium binding domain | | 8.109210738 | 4.37E-13 | cluster P | 1.29 |
| **RFC1** | replication factor C (activator 1) 1, 145kDa | | 4.573771061 | 1.20E-05 | cluster P | 1.16 |
| **TRIM27** | tripartite motif containing 27 | | 5.466732015 | 2.82E-07 | cluster P | 1.23 |
| **RFX1** | regulatory factor X, 1 (influences HLA class II expression) | | 7.766880474 | 7.44E-12 | cluster P | 1.32 |
| **BRD2** | bromodomain containing 2 | | 10.51994044 | 1.47E-17 | cluster P | 1.61 |
| **RNPEP** | arginyl aminopeptidase (aminopeptidase B) | | 10.04655634 | 3.43E-17 | cluster P | 1.46 |
| **RORA** | RAR-related orphan receptor A | | 4.098031798 | 7.59E-05 | cluster P | 1.17 |
| **RP2** | retinitis pigmentosa 2 (X-linked recessive) | | 6.578097385 | 1.76E-09 | cluster P | 1.44 |
| **RPL23A** | ribosomal protein L23a | | 6.688934067 | 9.83E-10 | cluster P | 1.48 |
| **RPL27A** | ribosomal protein L27a | | 4.587568484 | 1.50E-05 | cluster P | 1.22 |
| **RPL36AL** | ribosomal protein L36a-like | | 13.37663749 | 1.53E-25 | cluster P | 1.9 |
| **RPS6KA3** | ribosomal protein S6 kinase, 90kDa, polypeptide 3 | | 4.670878657 | 9.20E-06 | cluster P | 1.25 |
| **RPS13** | ribosomal protein S13 | | 15.51634413 | 1.07E-30 | cluster P | 2.36 |
| **RPS20** | ribosomal protein S20 | | 7.663687719 | 4.66E-12 | cluster P | 1.61 |
| **RPS27A** | ribosomal protein S27a | | 13.89386547 | 7.79E-26 | cluster P | 2.11 |
| **RRAS** | related RAS viral (r-ras) oncogene homolog | | 8.369720121 | 1.10E-13 | cluster P | 1.92 |
| **CLIP1** | CAP-GLY domain containing linker protein 1 | | 8.43798524 | 7.46E-14 | cluster P | 1.35 |
| **S100A3** | S100 calcium binding protein A3 | | 7.234799859 | 5.74E-11 | cluster P | 1.49 |
| **TSPAN31** | tetraspanin 31 | | 14.17358688 | 3.52E-26 | cluster P | 2.39 |
| **SAT1** | spermidine/spermine N1-acetyltransferase 1 | | 6.983726738 | 1.80E-10 | cluster P | 1.51 |
| **MAP2K4** | mitogen-activated protein kinase kinase 4 | | 10.3939513 | 2.15E-18 | cluster P | 1.68 |
| **STIL** | SCL/TAL1 interrupting locus | | 6.439542662 | 4.48E-09 | cluster P | 1.65 |
| **SIPA1** | signal-induced proliferation-associated 1 | | 8.889342947 | 3.01E-14 | cluster P | 1.45 |
| **SKI** | v-ski sarcoma viral oncogene homolog (avian) | | 5.931494478 | 3.01E-08 | cluster P | 1.31 |
| **SKIL** | SKI-like oncogene | | 6.661807553 | 9.81E-10 | cluster P | 1.79 |
| **SLC2A1** | solute carrier family 2 (facilitated glucose transporter), member 1 | | 5.585359075 | 1.63E-07 | cluster P | 2.04 |
| **SLCO2A1** | solute carrier organic anion transporter family, member 2A1 | | 6.637061591 | 1.48E-09 | cluster P | 1.41 |
| **SMARCA4** | SWI/SNF related, matrix associated, actin dependent regulator of chromatin, subfamily a, member 4 | | 3.977806393 | 0.000119865 | cluster P | 1.16 |
| **SMARCC1** | SWI/SNF related, matrix associated, actin dependent regulator of chromatin, subfamily c, member 1 | | 8.603288109 | 1.21E-13 | cluster P | 2.1 |
| **SMARCD1** | SWI/SNF related, matrix associated, actin dependent regulator of chromatin, subfamily d, member 1 | | 8.369498081 | 1.83E-13 | cluster P | 1.55 |
| **SMARCE1** | SWI/SNF related, matrix associated, actin dependent regulator of chromatin, subfamily e, member 1 | | 8.767367935 | 4.44E-14 | cluster P | 1.46 |
| **SNRPA1** | small nuclear ribonucleoprotein polypeptide A' | | 5.747761615 | 6.71E-08 | cluster P | 1.25 |
| **SNRPF** | small nuclear ribonucleoprotein polypeptide F | | 9.700403384 | 1.72E-16 | cluster P | 2.32 |
| **SNTB2** | syntrophin, beta 2 (dystrophin-associated protein A1, 59kDa, basic component 2) | | 9.779548527 | 4.99E-17 | cluster P | 1.82 |
| **SOAT1** | sterol O-acyltransferase 1 | | 3.948919121 | 0.000133257 | cluster P | 1.11 |
| **SOX4** | SRY (sex determining region Y)-box 4 | | 10.30882335 | 2.64E-17 | cluster P | 1.78 |
| **SP1** | Sp1 transcription factor | | 10.48868633 | 1.09E-16 | cluster P | 1.83 |
| **SP4** | Sp4 transcription factor | | 9.908970255 | 6.43E-17 | cluster P | 1.54 |
| **SPAST** | spastin | | 10.90661546 | 9.86E-20 | cluster P | 1.4 |
| **SPI1** | spleen focus forming virus (SFFV) proviral integration oncogene spi1 | | 9.326681591 | 5.93E-16 | cluster P | 1.72 |
| **SQLE** | squalene epoxidase | | 7.427427227 | 1.92E-11 | cluster P | 1.52 |
| **SRM** | spermidine synthase | | 9.0006104 | 2.45E-14 | cluster P | 2.07 |
| **SRPK1** | SRSF protein kinase 1 | | 9.916730767 | 2.92E-16 | cluster P | 2.05 |
| **ST13** | suppression of tumorigenicity 13 (colon carcinoma) (Hsp70 interacting protein) | | 14.46957212 | 1.15E-27 | cluster P | 2.37 |
| **STAT6** | signal transducer and activator of transcription 6, interleukin-4 induced | | 6.872666255 | 4.84E-10 | cluster P | 1.55 |
| **STK3** | serine/threonine kinase 3 | | 14.24864772 | 5.71E-27 | cluster P | 2.8 |
| **STK4** | serine/threonine kinase 4 | | 11.70541232 | 1.07E-20 | cluster P | 1.94 |
| **SUV39H1** | suppressor of variegation 3-9 homolog 1 (Drosophila) | | 9.441991492 | 8.74E-15 | cluster P | 2.09 |
| **TAF9** | TAF9 RNA polymerase II, TATA box binding protein (TBP)-associated factor, 32kDa | | 7.192809944 | 7.28E-11 | cluster P | 1.37 |
| **TAF10** | TAF10 RNA polymerase II, TATA box binding protein (TBP)-associated factor, 30kDa | | 13.9921317 | 3.56E-27 | cluster P | 2.22 |
| **MAP3K7** | mitogen-activated protein kinase kinase kinase 7 | | 6.301713408 | 5.66E-09 | cluster P | 1.29 |
| **TBX2** | T-box 2 | | 5.411778193 | 3.74E-07 | cluster P | 1.34 |
| **TCEB1** | transcription elongation factor B (SIII), polypeptide 1 (15kDa, elongin C) | | 7.315075675 | 3.00E-11 | cluster P | 1.36 |
| **TCF4** | transcription factor 4 | | 9.328300094 | 5.74E-16 | cluster P | 1.6 |
| **TCF7L2** | transcription factor 7-like 2 (T-cell specific, HMG-box) | | 6.365988804 | 3.52E-09 | cluster P | 1.25 |
| **ZEB1** | zinc finger E-box binding homeobox 1 | | 7.513355452 | 1.03E-11 | cluster P | 1.4 |
| **TCF12** | transcription factor 12 | | 12.69264174 | 3.28E-23 | cluster P | 1.91 |
| **PPP1R11** | protein phosphatase 1, regulatory (inhibitor) subunit 11 | | 6.345001322 | 4.00E-09 | cluster P | 1.57 |
| **TFE3** | transcription factor binding to IGHM enhancer 3 | | 10.90798361 | 8.92E-20 | cluster P | 1.38 |
| **TIAL1** | TIA1 cytotoxic granule-associated RNA binding protein-like 1 | | 9.394410585 | 3.93E-16 | cluster P | 1.44 |
| **TMPO** | thymopoietin | | 11.39513072 | 6.16E-21 | cluster P | 1.74 |
| **HSP90B1** | heat shock protein 90kDa beta (Grp94), member 1 | | 10.14585009 | 3.23E-17 | cluster P | 2.57 |
| **TSN** | translin | | 15.84395472 | 2.59E-31 | cluster P | 2.02 |
| **TSC1** | tuberous sclerosis 1 | | 8.149909768 | 3.71E-13 | cluster P | 1.37 |
| **TTC3** | tetratricopeptide repeat domain 3 | | 8.940298171 | 5.41E-15 | cluster P | 1.42 |
| **TUBA4A** | tubulin, alpha 4a | | 10.32249201 | 2.62E-18 | cluster P | 2.1 |
| **UBB** | ubiquitin B | | 11.28041479 | 1.40E-20 | cluster P | 2.07 |
| **UBE2E1** | ubiquitin-conjugating enzyme E2E 1 | | 7.6088442 | 4.96E-11 | cluster P | 1.38 |
| **UBE2N** | ubiquitin-conjugating enzyme E2N | | 9.314291552 | 1.56E-14 | cluster P | 1.79 |
| **UBE3A** | ubiquitin protein ligase E3A | | 8.858579159 | 1.74E-14 | cluster P | 1.73 |
| **SUMO1** | SMT3 suppressor of mif two 3 homolog 1 (S. cerevisiae) | | 5.654065662 | 1.37E-07 | cluster P | 1.18 |
| **UBP1** | upstream binding protein 1 (LBP-1a) | | 11.33077645 | 3.37E-20 | cluster P | 2.17 |
| **UGDH** | UDP-glucose 6-dehydrogenase | | 8.3013108 | 1.77E-13 | cluster P | 1.83 |
| **VTN** | vitronectin | | 5.194668014 | 1.38E-06 | cluster P | 1.8 |
| **WFS1** | Wolfram syndrome 1 (wolframin) | | 12.64070426 | 2.30E-23 | cluster P | 1.91 |
| **XBP1** | X-box binding protein 1 | | 6.303011698 | 5.64E-09 | cluster P | 1.59 |
| **XPO1** | exportin 1 (CRM1 homolog, yeast) | | 8.216330552 | 2.94E-13 | cluster P | 1.58 |
| **YES1** | v-yes-1 Yamaguchi sarcoma viral oncogene homolog 1 | | 14.39930058 | 2.05E-27 | cluster P | 2.23 |
| **YY1** | YY1 transcription factor | | 14.53338509 | 1.23E-25 | cluster P | 2.54 |
| **YWHAG** | tyrosine 3-monooxygenase/tryptophan 5-monooxygenase activation protein, gamma polypeptide | | 12.75352919 | 5.43E-22 | cluster P | 2.37 |
| **CNBP** | CCHC-type zinc finger, nucleic acid binding protein | | 8.416402095 | 4.43E-13 | cluster P | 1.63 |
| **MZF1** | myeloid zinc finger 1 | | 9.540216886 | 1.90E-16 | cluster P | 1.72 |
| **ZNF131** | zinc finger protein 131 | | 3.917575512 | 0.000173179 | cluster P | 1.15 |
| **ZNF148** | zinc finger protein 148 | | 4.376645673 | 2.60E-05 | cluster P | 1.11 |
| **VEZF1** | vascular endothelial zinc finger 1 | | 9.751277103 | 1.39E-16 | cluster P | 2.05 |
| **ZNF207** | zinc finger protein 207 | | 8.329724158 | 8.97E-13 | cluster P | 1.37 |
| **ZYX** | zyxin | | 9.312131073 | 7.04E-16 | cluster P | 1.7 |
| **PTP4A1** | protein tyrosine phosphatase type IVA, member 1 | | 4.141935313 | 7.32E-05 | cluster P | 1.23 |
| **FZD5** | frizzled family receptor 5 | | 3.639796362 | 0.00040235 | cluster P | 1.28 |
| **MAPKAPK3** | mitogen-activated protein kinase-activated protein kinase 3 | | 9.792174476 | 1.01E-16 | cluster P | 1.62 |
| **RAB7A** | RAB7A, member RAS oncogene family | | 15.52807673 | 8.43E-28 | cluster P | 3.2 |
| **SLC25A16** | solute carrier family 25 (mitochondrial carrier; Graves disease autoantigen), member 16 | | 6.151842173 | 1.17E-08 | cluster P | 1.4 |
| **SHOC2** | soc-2 suppressor of clear homolog (C. elegans) | | 3.441582467 | 0.000866927 | cluster P | 1.11 |
| **SLC7A5** | solute carrier family 7 (amino acid transporter light chain, L system), member 5 | | 4.493600233 | 1.76E-05 | cluster P | 1.66 |
| **AKAP1** | A kinase (PRKA) anchor protein 1 | | 9.817829548 | 7.99E-16 | cluster P | 2.06 |
| **LZTR1** | leucine-zipper-like transcription regulator 1 | | 8.64485455 | 1.36E-13 | cluster P | 1.46 |
| **USP11** | ubiquitin specific peptidase 11 | | 9.224018339 | 1.09E-15 | cluster P | 1.61 |
| **USP9X** | ubiquitin specific peptidase 9, X-linked | | 4.945945091 | 2.44E-06 | cluster P | 1.25 |
| **PICALM** | phosphatidylinositol binding clathrin assembly protein | | 12.20994654 | 8.74E-23 | cluster P | 1.57 |
| **BAP1** | BRCA1 associated protein-1 (ubiquitin carboxy-terminal hydrolase) | | 7.221431539 | 4.76E-11 | cluster P | 1.32 |
| **HIST1H2AJ** | histone cluster 1, H2aj | | 5.101521616 | 1.53E-06 | cluster P | 1.44 |
| **HIST2H2AC** | histone cluster 2, H2ac | | 9.88354801 | 4.10E-16 | cluster P | 2.35 |
| **HIST1H2BO** | histone cluster 1, H2bo | | 5.349554033 | 6.16E-07 | cluster P | 1.46 |
| **SPOP** | speckle-type POZ protein | | 7.680298818 | 8.53E-12 | cluster P | 1.34 |
| **ULK1** | unc-51-like kinase 1 (C. elegans) | | 9.301305381 | 6.61E-16 | cluster P | 1.99 |
| **CUL4B** | cullin 4B | | 10.4771222 | 1.02E-18 | cluster P | 1.75 |
| **KLF11** | Kruppel-like factor 11 | | 7.631646176 | 6.65E-12 | cluster P | 1.48 |
| **AGPS** | alkylglycerone phosphate synthase | | 7.420426779 | 1.68E-11 | cluster P | 1.49 |
| **LMO4** | LIM domain only 4 | | 6.887466476 | 3.01E-10 | cluster P | 1.73 |
| **YARS** | tyrosyl-tRNA synthetase | | 8.996688981 | 7.59E-15 | cluster P | 1.52 |
| **RUVBL1** | RuvB-like 1 (E. coli) | | 6.231843387 | 7.88E-09 | cluster P | 1.33 |
| **PPAP2A** | phosphatidic acid phosphatase type 2A | | 8.944716129 | 5.86E-15 | cluster P | 1.63 |
| **CHRD** | chordin | | 4.556999519 | 1.32E-05 | cluster P | 1.31 |
| **DYNLL1** | dynein, light chain, LC8-type 1 | | 8.068141761 | 6.52E-13 | cluster P | 1.45 |
| **EIF3J** | eukaryotic translation initiation factor 3, subunit J | | 8.5464996 | 4.18E-14 | cluster P | 1.46 |
| **EIF4G3** | eukaryotic translation initiation factor 4 gamma, 3 | | 6.326938079 | 5.19E-09 | cluster P | 1.3 |
| **PEA15** | phosphoprotein enriched in astrocytes 15 | | 14.27548822 | 3.30E-27 | cluster P | 2.24 |
| **CDC23** | cell division cycle 23 | | 10.35758949 | 7.56E-18 | cluster P | 1.76 |
| **B4GALT3** | UDP-Gal:betaGlcNAc beta 1,4- galactosyltransferase, polypeptide 3 | | 11.70762667 | 2.01E-21 | cluster P | 1.98 |
| **RNMT** | RNA (guanine-7-) methyltransferase | | 4.90728783 | 3.65E-06 | cluster P | 1.27 |
| **RNGTT** | RNA guanylyltransferase and 5'-phosphatase | | 13.01831264 | 1.41E-24 | cluster P | 1.75 |
| **CD164** | CD164 molecule, sialomucin | | 7.750106868 | 7.23E-12 | cluster P | 1.57 |
| **RAB11A** | RAB11A, member RAS oncogene family | | 8.200967624 | 3.75E-13 | cluster P | 1.9 |
| **CCNK** | cyclin K | | 12.53741164 | 1.80E-23 | cluster P | 1.73 |
| **GGH** | gamma-glutamyl hydrolase (conjugase, folylpolygammaglutamyl hydrolase) | | 7.283111335 | 2.59E-10 | cluster P | 2.04 |
| **TSC22D1** | TSC22 domain family, member 1 | | 9.035662998 | 2.94E-15 | cluster P | 1.99 |
| **PER2** | period circadian clock 2 | | 5.731101712 | 7.40E-08 | cluster P | 1.43 |
| **MTMR3** | myotubularin related protein 3 | | 4.094779382 | 8.86E-05 | cluster P | 1.17 |
| **USP13** | ubiquitin specific peptidase 13 (isopeptidase T-3) | | 8.756446651 | 1.37E-14 | cluster P | 1.33 |
| **TAF1B** | TATA box binding protein (TBP)-associated factor, RNA polymerase I, B, 63kDa | | 6.967287763 | 1.76E-10 | cluster P | 1.2 |
| **UBE2M** | ubiquitin-conjugating enzyme E2M | | 4.150083385 | 7.15E-05 | cluster P | 1.18 |
| **SPAG9** | sperm associated antigen 9 | | 9.139527551 | 2.76E-15 | cluster P | 1.63 |
| **MAP7** | microtubule-associated protein 7 | | 9.823053753 | 4.53E-17 | cluster P | 1.97 |
| **MTMR4** | myotubularin related protein 4 | | 8.836031796 | 9.58E-15 | cluster P | 1.53 |
| **BUB3** | BUB3 mitotic checkpoint protein | | 4.699255127 | 8.14E-06 | cluster P | 1.2 |
| **SLC33A1** | solute carrier family 33 (acetyl-CoA transporter), member 1 | | 9.387911551 | 9.10E-16 | cluster P | 1.49 |
| **ZMYM4** | zinc finger, MYM-type 4 | | 7.37302556 | 2.92E-11 | cluster P | 1.7 |
| **CYTH3** | cytohesin 3 | | 9.498300434 | 3.33E-16 | cluster P | 1.4 |
| **B4GALT5** | UDP-Gal:betaGlcNAc beta 1,4- galactosyltransferase, polypeptide 5 | | 6.489246536 | 2.07E-09 | cluster P | 1.43 |
| **SLC9A3R2** | solute carrier family 9, subfamily A (NHE3, cation proton antiporter 3), member 3 regulator 2 | | 7.778492553 | 3.27E-12 | cluster P | 1.61 |
| **PPIG** | peptidylprolyl isomerase G (cyclophilin G) | | 3.673374112 | 0.000390213 | cluster P | 1.23 |
| **SLC9A3R1** | solute carrier family 9, subfamily A (NHE3, cation proton antiporter 3), member 3 regulator 1 | | 7.074563911 | 2.21E-10 | cluster P | 1.63 |
| **TM9SF2** | transmembrane 9 superfamily member 2 | | 11.75616707 | 1.00E-21 | cluster P | 1.95 |
| **COX5A** | cytochrome c oxidase subunit Va | | 10.20094117 | 8.46E-17 | cluster P | 1.89 |
| **LPXN** | leupaxin | | 8.718353824 | 3.16E-13 | cluster P | 2.11 |
| **ZRANB2** | zinc finger, RAN-binding domain containing 2 | | 6.95308894 | 2.21E-10 | cluster P | 1.35 |
| **SCAMP1** | secretory carrier membrane protein 1 | | 13.92877705 | 9.79E-27 | cluster P | 2.07 |
| **TMEM59** | transmembrane protein 59 | | 11.14513545 | 7.07E-20 | cluster P | 2.39 |
| **GMFG** | glia maturation factor, gamma | | 8.427658792 | 1.15E-13 | cluster P | 1.55 |
| **APBA3** | amyloid beta (A4) precursor protein-binding, family A, member 3 | | 5.493049772 | 2.59E-07 | cluster P | 1.55 |
| **H2AFY** | H2A histone family, member Y | | 4.242667954 | 5.00E-05 | cluster P | 1.2 |
| **CLOCK** | clock circadian regulator | | 8.60753705 | 3.94E-14 | cluster P | 1.24 |
| **PRDX6** | peroxiredoxin 6 | | 7.965379081 | 1.42E-12 | cluster P | 1.63 |
| **WTAP** | Wilms tumor 1 associated protein | | 4.189956464 | 7.03E-05 | cluster P | 1.22 |
| **NCOR1** | nuclear receptor corepressor 1 | | 3.803672353 | 0.000281222 | cluster P | 1.15 |
| **SH3PXD2A** | SH3 and PX domains 2A | | 10.8303201 | 1.57E-17 | cluster P | 2.53 |
| **MTFR1** | mitochondrial fission regulator 1 | | 6.574938399 | 1.60E-09 | cluster P | 1.36 |
| **EIF5B** | eukaryotic translation initiation factor 5B | | 9.920330904 | 1.84E-15 | cluster P | 2.41 |
| **USP6NL** | USP6 N-terminal like | | 12.08945099 | 7.62E-22 | cluster P | 1.53 |
| **SLK** | STE20-like kinase | | 8.502694498 | 6.57E-14 | cluster P | 1.27 |
| **HDAC4** | histone deacetylase 4 | | 7.41065294 | 1.76E-11 | cluster P | 1.61 |
| **MTSS1** | metastasis suppressor 1 | | 11.2639025 | 4.65E-19 | cluster P | 2.53 |
| **SERTAD2** | SERTA domain containing 2 | | 9.208263171 | 4.58E-15 | cluster P | 1.71 |
| **TSC22D2** | TSC22 domain family, member 2 | | 9.81976797 | 1.42E-15 | cluster P | 1.92 |
| **CUL7** | cullin 7 | | 6.436408752 | 2.51E-09 | cluster P | 1.28 |
| **EPM2AIP1** | EPM2A (laforin) interacting protein 1 | | 5.734183808 | 8.25E-08 | cluster P | 1.3 |
| **PJA2** | praja ring finger 2, E3 ubiquitin protein ligase | | 9.432876684 | 4.99E-16 | cluster P | 1.58 |
| **TLK1** | tousled-like kinase 1 | | 14.30212064 | 6.31E-28 | cluster P | 2.16 |
| **UBAP2L** | ubiquitin associated protein 2-like | | 9.797083778 | 7.70E-17 | cluster P | 1.64 |
| **FAM20B** | family with sequence similarity 20, member B | | 5.875923031 | 5.10E-08 | cluster P | 1.2 |
| **JOSD1** | Josephin domain containing 1 | | 12.88466624 | 5.41E-24 | cluster P | 1.99 |
| **OXSR1** | oxidative-stress responsive 1 | | 6.926101746 | 2.18E-10 | cluster P | 1.5 |
| **AMMECR1** | Alport syndrome, mental retardation, midface hypoplasia and elliptocytosis chromosomal region gene 1 | | 7.660034258 | 4.77E-12 | cluster P | 1.35 |
| **HS3ST3A1** | heparan sulfate (glucosamine) 3-O-sulfotransferase 3A1 | | 4.607248055 | 1.38E-05 | cluster P | 1.49 |
| **DMTF1** | cyclin D binding myb-like transcription factor 1 | | 8.521216337 | 6.39E-14 | cluster P | 1.44 |
| **SLC12A6** | solute carrier family 12 (potassium/chloride transporters), member 6 | | 7.558251971 | 9.68E-12 | cluster P | 1.34 |
| **SAE1** | SUMO1 activating enzyme subunit 1 | | 7.323601133 | 4.90E-11 | cluster P | 1.67 |
| **ABCB6** | ATP-binding cassette, sub-family B (MDR/TAP), member 6 | | 11.45603833 | 9.37E-19 | cluster P | 1.93 |
| **SCAMP3** | secretory carrier membrane protein 3 | | 4.401649405 | 2.72E-05 | cluster P | 1.21 |
| **IL18BP** | interleukin 18 binding protein | | 5.436265822 | 4.76E-07 | cluster P | 1.27 |
| **ACTR3** | ARP3 actin-related protein 3 homolog (yeast) | | 14.84154206 | 4.50E-27 | cluster P | 2.25 |
| **TSPAN3** | tetraspanin 3 | | 6.076300104 | 1.74E-08 | cluster P | 1.45 |
| **ARL4A** | ADP-ribosylation factor-like 4A | | 6.435972563 | 4.38E-09 | cluster P | 1.34 |
| **RBM12** | RNA binding motif protein 12 | | 11.36344617 | 1.01E-20 | cluster P | 1.54 |
| **TOB1** | transducer of ERBB2, 1 | | 6.116972967 | 1.40E-08 | cluster P | 1.54 |
| **MBNL2** | muscleblind-like splicing regulator 2 | | 9.130732109 | 7.36E-14 | cluster P | 1.83 |
| **ABI2** | abl-interactor 2 | | 5.812153545 | 5.92E-08 | cluster P | 1.22 |
| **CEBPZ** | CCAAT/enhancer binding protein (C/EBP), zeta | | 11.11380766 | 6.07E-19 | cluster P | 1.62 |
| **ATP6AP2** | ATPase, H+ transporting, lysosomal accessory protein 2 | | 9.924741686 | 3.12E-17 | cluster P | 1.6 |
| **LPCAT3** | lysophosphatidylcholine acyltransferase 3 | | 7.646017126 | 5.57E-12 | cluster P | 1.49 |
| **SLC25A13** | solute carrier family 25 (aspartate/glutamate carrier), member 13 | | 14.35192741 | 5.54E-28 | cluster P | 1.76 |
| **MPZL2** | myelin protein zero-like 2 | | 10.25233082 | 4.49E-18 | cluster P | 2.13 |
| **CTDSPL** | CTD (carboxy-terminal domain, RNA polymerase II, polypeptide A) small phosphatase-like | | 8.801757731 | 1.15E-14 | cluster P | 1.91 |
| **SPRY2** | sprouty homolog 2 (Drosophila) | | 9.078701262 | 4.01E-15 | cluster P | 1.72 |
| **DENND4A** | DENN/MADD domain containing 4A | | 9.014089996 | 3.44E-15 | cluster P | 1.41 |
| **IKZF1** | IKAROS family zinc finger 1 (Ikaros) | | 7.563004833 | 2.99E-11 | cluster P | 2.17 |
| **TMEM5** | transmembrane protein 5 | | 7.481651138 | 1.83E-11 | cluster P | 1.66 |
| **IFITM3** | interferon induced transmembrane protein 3 | | 9.924774754 | 6.37E-16 | cluster P | 1.74 |
| **YAP1** | Yes-associated protein 1 | | 6.514791919 | 2.43E-09 | cluster P | 1.42 |
| **RBM14** | RNA binding motif protein 14 | | 13.61205512 | 3.29E-26 | cluster P | 2.29 |
| **MCRS1** | microspherule protein 1 | | 11.3509472 | 2.78E-20 | cluster P | 1.78 |
| **MAD2L2** | MAD2 mitotic arrest deficient-like 2 (yeast) | | 4.438626268 | 1.99E-05 | cluster P | 1.37 |
| **PIBF1** | progesterone immunomodulatory binding factor 1 | | 5.411576104 | 3.93E-07 | cluster P | 1.3 |
| **UBE2E3** | ubiquitin-conjugating enzyme E2E 3 | | 6.803248209 | 3.96E-10 | cluster P | 1.33 |
| **SYNCRIP** | synaptotagmin binding, cytoplasmic RNA interacting protein | | 8.954189509 | 9.29E-15 | cluster P | 1.66 |
| **SEMA4B** | sema domain, immunoglobulin domain (Ig), transmembrane domain (TM) and short cytoplasmic domain, (semaphorin) 4B | | 4.167919766 | 5.75E-05 | cluster P | 1.33 |
| **APPBP2** | amyloid beta precursor protein (cytoplasmic tail) binding protein 2 | | 10.07496302 | 9.89E-17 | cluster P | 1.63 |
| **RNASEH2A** | ribonuclease H2, subunit A | | 6.747761546 | 6.16E-10 | cluster P | 1.35 |
| **PRDX4** | peroxiredoxin 4 | | 13.24575166 | 1.79E-23 | cluster P | 2.16 |
| **TRIM3** | tripartite motif containing 3 | | 7.945826619 | 1.69E-12 | cluster P | 1.46 |
| **TXNIP** | thioredoxin interacting protein | | 4.779160573 | 6.68E-06 | cluster P | 1.15 |
| **KHDRBS1** | KH domain containing, RNA binding, signal transduction associated 1 | | 9.78707148 | 6.39E-17 | cluster P | 1.61 |
| **RRAGA** | Ras-related GTP binding A | | 6.269489094 | 5.72E-09 | cluster P | 1.56 |
| **GNA13** | guanine nucleotide binding protein (G protein), alpha 13 | | 12.83905916 | 1.23E-21 | cluster P | 2.65 |
| **B3GNT2** | UDP-GlcNAc:betaGal beta-1,3-N-acetylglucosaminyltransferase 2 | | 5.927793639 | 4.76E-08 | cluster P | 1.43 |
| **CCT8** | chaperonin containing TCP1, subunit 8 (theta) | | 6.078246 | 2.29E-08 | cluster P | 1.25 |
| **NFAT5** | nuclear factor of activated T-cells 5, tonicity-responsive | | 3.679721459 | 0.000355545 | cluster P | 1.17 |
| **PTGES3** | prostaglandin E synthase 3 (cytosolic) | | 14.16260718 | 2.56E-25 | cluster P | 3.02 |
| **YME1L1** | YME1-like 1 (S. cerevisiae) | | 11.81724388 | 5.42E-22 | cluster P | 1.89 |
| **RAI1** | retinoic acid induced 1 | | 7.596252226 | 9.20E-12 | cluster P | 1.44 |
| **AHCYL1** | adenosylhomocysteinase-like 1 | | 11.51521946 | 3.88E-21 | cluster P | 1.74 |
| **ZMYND11** | zinc finger, MYND-type containing 11 | | 6.932396743 | 2.95E-10 | cluster P | 1.29 |
| **IQGAP2** | IQ motif containing GTPase activating protein 2 | | 8.19024567 | 7.06E-13 | cluster P | 1.38 |
| **RAB10** | RAB10, member RAS oncogene family | | 10.99468083 | 2.71E-18 | cluster P | 2.08 |
| **MALT1** | mucosa associated lymphoid tissue lymphoma translocation gene 1 | | 7.950529033 | 1.14E-12 | cluster P | 1.35 |
| **PNPLA6** | patatin-like phospholipase domain containing 6 | | 4.535742597 | 1.90E-05 | cluster P | 1.22 |
| **PAPOLA** | poly(A) polymerase alpha | | 14.61818547 | 1.70E-25 | cluster P | 3.23 |
| **TCERG1** | transcription elongation regulator 1 | | 9.800298195 | 9.06E-17 | cluster P | 1.4 |
| **SUB1** | SUB1 homolog (S. cerevisiae) | | 8.005621389 | 4.22E-12 | cluster P | 1.94 |
| **RALBP1** | ralA binding protein 1 | | 11.74957955 | 7.94E-22 | cluster P | 1.62 |
| **SEC61B** | Sec61 beta subunit | | 10.36161192 | 7.68E-18 | cluster P | 1.71 |
| **TMED2** | transmembrane emp24 domain trafficking protein 2 | | 8.028580003 | 1.68E-12 | cluster P | 1.54 |
| **TMED10** | transmembrane emp24-like trafficking protein 10 (yeast) | | 10.33071514 | 4.98E-18 | cluster P | 1.75 |
| **CCNI** | cyclin I | | 9.80909472 | 3.94E-17 | cluster P | 2.14 |
| **ILVBL** | ilvB (bacterial acetolactate synthase)-like | | 8.292587238 | 2.01E-13 | cluster P | 1.59 |
| **SLC27A3** | solute carrier family 27 (fatty acid transporter), member 3 | | 8.07016322 | 5.88E-13 | cluster P | 1.32 |
| **RAB35** | RAB35, member RAS oncogene family | | 12.47326907 | 4.10E-23 | cluster P | 1.7 |
| **PIM2** | pim-2 oncogene | | 4.280555579 | 4.60E-05 | cluster P | 1.59 |
| **DUS4L** | dihydrouridine synthase 4-like (S. cerevisiae) | | 8.646586785 | 3.89E-14 | cluster P | 1.33 |
| **CYB561D2** | cytochrome b561 family, member D2 | | 6.069921536 | 1.62E-08 | cluster P | 1.38 |
| **RAPGEF4** | Rap guanine nucleotide exchange factor (GEF) 4 | | 7.429434232 | 1.71E-11 | cluster P | 1.22 |
| **HIBADH** | 3-hydroxyisobutyrate dehydrogenase | | 8.552662223 | 1.62E-13 | cluster P | 1.96 |
| **CIT** | citron (rho-interacting, serine/threonine kinase 21) | | 9.076032518 | 5.57E-15 | cluster P | 1.52 |
| **FAF1** | Fas (TNFRSF6) associated factor 1 | | 10.32022088 | 2.84E-18 | cluster P | 1.51 |
| **NUDT3** | nudix (nucleoside diphosphate linked moiety X)-type motif 3 | | 13.53779027 | 2.47E-25 | cluster P | 2.11 |
| **STRAP** | serine/threonine kinase receptor associated protein | | 9.103197382 | 3.68E-15 | cluster P | 1.36 |
| **SEC63** | SEC63 homolog (S. cerevisiae) | | 12.42040475 | 1.20E-22 | cluster P | 1.66 |
| **PDCD10** | programmed cell death 10 | | 7.655651878 | 7.02E-12 | cluster P | 1.21 |
| **PACSIN2** | protein kinase C and casein kinase substrate in neurons 2 | | 5.989101191 | 2.17E-08 | cluster P | 1.85 |
| **DDX42** | DEAD (Asp-Glu-Ala-Asp) box polypeptide 42 | | 7.774377566 | 2.90E-12 | cluster P | 1.36 |
| **U2AF2** | U2 small nuclear RNA auxiliary factor 2 | | 11.46313971 | 4.45E-21 | cluster P | 1.74 |
| **MTF2** | metal response element binding transcription factor 2 | | 5.378306384 | 6.67E-07 | cluster P | 1.31 |
| **PUF60** | poly-U binding splicing factor 60KDa | | 6.278301206 | 5.82E-09 | cluster P | 1.53 |
| **ZHX2** | zinc fingers and homeoboxes 2 | | 3.753177602 | 0.00026867 | cluster P | 1.39 |
| **FOXJ3** | forkhead box J3 | | 6.283810736 | 5.98E-09 | cluster P | 1.82 |
| **MAPRE1** | microtubule-associated protein, RP/EB family, member 1 | | 9.839865563 | 3.33E-17 | cluster P | 1.62 |
| **ATF6** | activating transcription factor 6 | | 8.639098649 | 3.36E-14 | cluster P | 1.8 |
| **SEPHS2** | selenophosphate synthetase 2 | | 11.50468877 | 3.44E-21 | cluster P | 1.8 |
| **RAB3GAP1** | RAB3 GTPase activating protein subunit 1 (catalytic) | | 6.087657641 | 1.89E-08 | cluster P | 1.76 |
| **TCF25** | transcription factor 25 (basic helix-loop-helix) | | 13.51314092 | 4.59E-26 | cluster P | 2.12 |
| **DAAM1** | dishevelled associated activator of morphogenesis 1 | | 8.871198932 | 4.79E-14 | cluster P | 1.48 |
| **KLHDC10** | kelch domain containing 10 | | 5.228318236 | 7.12E-07 | cluster P | 1.27 |
| **CNOT1** | CCR4-NOT transcription complex, subunit 1 | | 9.122046322 | 6.14E-15 | cluster P | 1.63 |
| **MON2** | MON2 homolog (S. cerevisiae) | | 11.97336517 | 1.29E-19 | cluster P | 2.67 |
| **FNBP1** | formin binding protein 1 | | 7.408552308 | 1.86E-11 | cluster P | 1.57 |
| **SWAP70** | SWAP switching B-cell complex 70kDa subunit | | 7.268639968 | 5.77E-11 | cluster P | 1.54 |
| **MYCBP2** | MYC binding protein 2, E3 ubiquitin protein ligase | | 8.882394577 | 1.07E-14 | cluster P | 1.95 |
| **PPRC1** | peroxisome proliferator-activated receptor gamma, coactivator-related 1 | | 12.56169794 | 3.07E-23 | cluster P | 2.21 |
| **TNRC6B** | trinucleotide repeat containing 6B | | 8.863190685 | 9.23E-15 | cluster P | 1.42 |
| **FAM179B** | family with sequence similarity 179, member B | | 7.909942616 | 1.47E-12 | cluster P | 1.6 |
| **DCUN1D4** | DCN1, defective in cullin neddylation 1, domain containing 4 (S. cerevisiae) | | 11.72270998 | 1.26E-21 | cluster P | 1.91 |
| **TBC1D9** | TBC1 domain family, member 9 (with GRAM domain) | | 6.808754502 | 3.87E-10 | cluster P | 1.39 |
| **NUP205** | nucleoporin 205kDa | | 7.856965885 | 2.45E-12 | cluster P | 1.41 |
| **UBXN4** | UBX domain protein 4 | | 15.34466809 | 1.89E-29 | cluster P | 2.32 |
| **FAM120A** | family with sequence similarity 120A | | 18.01015733 | 1.00E-32 | cluster P | 2.28 |
| **FAF2** | Fas associated factor family member 2 | | 12.78915264 | 2.78E-24 | cluster P | 2.21 |
| **PSME4** | proteasome (prosome, macropain) activator subunit 4 | | 6.944210591 | 3.31E-10 | cluster P | 1.6 |
| **ATP11B** | ATPase, class VI, type 11B | | 9.815237231 | 9.13E-17 | cluster P | 1.39 |
| **ARL6IP1** | ADP-ribosylation factor-like 6 interacting protein 1 | | 5.207918142 | 7.91E-07 | cluster P | 1.34 |
| **PDS5A** | PDS5, regulator of cohesion maintenance, homolog A (S. cerevisiae) | | 6.458467211 | 4.16E-09 | cluster P | 1.48 |
| **ANKRD12** | ankyrin repeat domain 12 | | 7.963408227 | 9.69E-13 | cluster P | 1.38 |
| **SCFD1** | sec1 family domain containing 1 | | 10.74101259 | 2.71E-19 | cluster P | 1.54 |
| **PHF15** | PHD finger protein 15 | | 4.235150574 | 4.45E-05 | cluster P | 1.18 |
| **DOCK9** | dedicator of cytokinesis 9 | | 6.705792802 | 6.74E-10 | cluster P | 1.42 |
| **ARHGEF12** | Rho guanine nucleotide exchange factor (GEF) 12 | | 10.06505784 | 1.06E-16 | cluster P | 1.99 |
| **ARHGEF18** | Rho/Rac guanine nucleotide exchange factor (GEF) 18 | | 9.864472146 | 6.38E-17 | cluster P | 2.38 |
| **SMG5** | smg-5 homolog, nonsense mediated mRNA decay factor (C. elegans) | | 10.25999974 | 3.20E-18 | cluster P | 1.74 |
| **SPECC1L** | sperm antigen with calponin homology and coiled-coil domains 1-like | | 13.57198963 | 2.53E-25 | cluster P | 2.6 |
| **RYBP** | RING1 and YY1 binding protein | | 8.21742022 | 7.88E-13 | cluster P | 1.87 |
| **RHOQ** | ras homolog family member Q | | 8.645550852 | 2.97E-14 | cluster P | 1.55 |
| **HARS2** | histidyl-tRNA synthetase 2, mitochondrial | | 12.80782566 | 2.20E-23 | cluster P | 1.84 |
| **CBX5** | chromobox homolog 5 | | 8.124240707 | 7.02E-13 | cluster P | 1.49 |
| **LEPROTL1** | leptin receptor overlapping transcript-like 1 | | 4.069744667 | 8.37E-05 | cluster P | 1.42 |
| **MORC3** | MORC family CW-type zinc finger 3 | | 4.203916472 | 5.04E-05 | cluster P | 1.16 |
| **SKIV2L2** | superkiller viralicidic activity 2-like 2 (S. cerevisiae) | | 9.044713668 | 3.70E-15 | cluster P | 1.59 |
| **R3HDM1** | R3H domain containing 1 | | 9.774171182 | 2.32E-16 | cluster P | 1.58 |
| **SRRM2** | serine/arginine repetitive matrix 2 | | 13.20356994 | 1.42E-23 | cluster P | 2.02 |
| **WBP1** | WW domain binding protein 1 | | 6.69250055 | 1.20E-09 | cluster P | 1.31 |
| **GTPBP4** | GTP binding protein 4 | | 13.53613598 | 6.45E-25 | cluster P | 2.1 |
| **KLHDC2** | kelch domain containing 2 | | 12.23608454 | 2.41E-22 | cluster P | 1.81 |
| **LEMD3** | LEM domain containing 3 | | 9.192697529 | 1.39E-15 | cluster P | 1.72 |
| **CORO1C** | coronin, actin binding protein, 1C | | 7.889256281 | 1.50E-12 | cluster P | 1.32 |
| **MKRN1** | makorin ring finger protein 1 | | 11.33534744 | 8.19E-21 | cluster P | 1.75 |
| **PHLDA3** | pleckstrin homology-like domain, family A, member 3 | | 4.444441103 | 1.95E-05 | cluster P | 1.16 |
| **STX12** | syntaxin 12 | | 5.285327846 | 7.09E-07 | cluster P | 1.22 |
| **SH3BP4** | SH3-domain binding protein 4 | | 10.12501923 | 1.94E-17 | cluster P | 1.97 |
| **MTCH1** | mitochondrial carrier 1 | | 14.95235968 | 2.55E-29 | cluster P | 2.11 |
| **FBXL2** | F-box and leucine-rich repeat protein 2 | | 5.282215586 | 5.85E-07 | cluster P | 1.28 |
| **NIPBL** | Nipped-B homolog (Drosophila) | | 7.3120145 | 7.81E-11 | cluster P | 1.42 |
| **METTL7A** | methyltransferase like 7A | | 5.968626943 | 2.38E-08 | cluster P | 1.54 |
| **YIPF3** | Yip1 domain family, member 3 | | 15.48382637 | 4.42E-29 | cluster P | 2.54 |
| **ABI3BP** | ABI family, member 3 (NESH) binding protein | | 7.065687621 | 1.06E-10 | cluster P | 1.24 |
| **RNF19A** | ring finger protein 19A, E3 ubiquitin protein ligase | | 7.367545881 | 1.14E-10 | cluster P | 1.9 |
| **TMEM158** | transmembrane protein 158 (gene/pseudogene) | | 6.965621602 | 1.88E-10 | cluster P | 1.87 |
| **C1orf43** | chromosome 1 open reading frame 43 | | 5.295411582 | 7.01E-07 | cluster P | 1.2 |
| **ZDHHC5** | zinc finger, DHHC-type containing 5 | | 12.53934372 | 1.14E-23 | cluster P | 1.67 |
| **FAM98A** | family with sequence similarity 98, member A | | 6.908562882 | 6.90E-10 | cluster P | 1.34 |
| **PVRL3** | poliovirus receptor-related 3 | | 8.698825155 | 4.39E-14 | cluster P | 2 |
| **SYF2** | SYF2 homolog, RNA splicing factor (S. cerevisiae) | | 5.611272342 | 3.20E-07 | cluster P | 1.32 |
| **TIPARP** | TCDD-inducible poly(ADP-ribose) polymerase | | 5.673784249 | 9.46E-08 | cluster P | 1.17 |
| **TSKU** | tsukushi, small leucine rich proteoglycan | | 3.847523292 | 0.000199362 | cluster P | 1.28 |
| **IBTK** | inhibitor of Bruton agammaglobulinemia tyrosine kinase | | 11.68680668 | 2.84E-21 | cluster P | 2.05 |
| **ZZZ3** | zinc finger, ZZ-type containing 3 | | 8.059956764 | 6.11E-13 | cluster P | 1.62 |
| **UPF2** | UPF2 regulator of nonsense transcripts homolog (yeast) | | 4.35246769 | 3.35E-05 | cluster P | 1.24 |
| **WSB1** | WD repeat and SOCS box containing 1 | | 3.841576404 | 0.000222108 | cluster P | 1.14 |
| **NAT9** | N-acetyltransferase 9 (GCN5-related, putative) | | 10.46657199 | 1.01E-18 | cluster P | 1.41 |
| **FAM162A** | family with sequence similarity 162, member A | | 7.460526836 | 1.53E-11 | cluster P | 1.71 |
| **BSCL2** | Berardinelli-Seip congenital lipodystrophy 2 (seipin) | | 12.91541002 | 2.60E-24 | cluster P | 2.2 |
| **TRUB2** | TruB pseudouridine (psi) synthase homolog 2 (E. coli) | | 7.00560826 | 1.48E-10 | cluster P | 1.29 |
| **NGFRAP1** | nerve growth factor receptor (TNFRSF16) associated protein 1 | | 5.168338136 | 1.26E-06 | cluster P | 1.24 |
| **PPA2** | pyrophosphatase (inorganic) 2 | | 10.23799001 | 6.05E-18 | cluster P | 1.51 |
| **RPUSD2** | RNA pseudouridylate synthase domain containing 2 | | 8.166026009 | 3.37E-13 | cluster P | 1.63 |
| **TRAPPC3** | trafficking protein particle complex 3 | | 8.414225 | 1.77E-12 | cluster P | 1.44 |
| **FAM155B** | family with sequence similarity 155, member B | | 4.860771602 | 4.82E-06 | cluster P | 1.67 |
| **AFF4** | AF4/FMR2 family, member 4 | | 10.13578828 | 1.99E-17 | cluster P | 1.38 |
| **LSM1** | LSM1 homolog, U6 small nuclear RNA associated (S. cerevisiae) | | 9.200649359 | 1.47E-15 | cluster P | 2.05 |
| **SRPX2** | sushi-repeat containing protein, X-linked 2 | | 4.901547557 | 3.27E-06 | cluster P | 1.32 |
| **MRPS28** | mitochondrial ribosomal protein S28 | | 8.645118308 | 2.74E-14 | cluster P | 1.5 |
| **SPCS1** | signal peptidase complex subunit 1 homolog (S. cerevisiae) | | 5.19502704 | 1.32E-06 | cluster P | 1.18 |
| **MRPS18B** | mitochondrial ribosomal protein S18B | | 10.4415411 | 2.88E-18 | cluster P | 1.33 |
| **ZBTB44** | zinc finger and BTB domain containing 44 | | 4.607832035 | 1.18E-05 | cluster P | 1.22 |
| **RANGRF** | RAN guanine nucleotide release factor | | 4.778023453 | 5.61E-06 | cluster P | 1.18 |
| **ANKRD11** | ankyrin repeat domain 11 | | 6.606856775 | 1.06E-09 | cluster P | 1.26 |
| **GNL2** | guanine nucleotide binding protein-like 2 (nucleolar) | | 4.987351355 | 3.12E-06 | cluster P | 1.26 |
| **SNX11** | sorting nexin 11 | | 13.61478844 | 9.22E-26 | cluster P | 1.77 |
| **BAZ2B** | bromodomain adjacent to zinc finger domain, 2B | | 10.57158468 | 4.86E-18 | cluster P | 1.69 |
| **SLC40A1** | solute carrier family 40 (iron-regulated transporter), member 1 | | 10.30983114 | 1.05E-17 | cluster P | 2.49 |
| **NOX4** | NADPH oxidase 4 | | 8.062533844 | 7.10E-13 | cluster P | 1.99 |
| **AK3** | adenylate kinase 3 | | 10.52243597 | 7.13E-18 | cluster P | 1.83 |
| **TMED5** | transmembrane emp24 protein transport domain containing 5 | | 10.72096516 | 1.11E-18 | cluster P | 1.95 |
| **TMED7** | transmembrane emp24 protein transport domain containing 7 | | 11.64295307 | 1.43E-19 | cluster P | 2.26 |
| **RRP15** | ribosomal RNA processing 15 homolog (S. cerevisiae) | | 4.84604907 | 4.22E-06 | cluster P | 1.56 |
| **GLOD4** | glyoxalase domain containing 4 | | 11.79189727 | 2.16E-20 | cluster P | 2.03 |
| **UBXN1** | UBX domain protein 1 | | 10.00874658 | 1.43E-17 | cluster P | 1.97 |
| **TXNDC12** | thioredoxin domain containing 12 (endoplasmic reticulum) | | 5.468490564 | 2.61E-07 | cluster P | 1.46 |
| **MRPL2** | mitochondrial ribosomal protein L2 | | 4.544588047 | 1.38E-05 | cluster P | 1.2 |
| **SH3GLB1** | SH3-domain GRB2-like endophilin B1 | | 10.79070267 | 6.20E-19 | cluster P | 2.07 |
| **PHF20L1** | PHD finger protein 20-like 1 | | 9.348755747 | 1.31E-15 | cluster P | 1.47 |
| **IER3IP1** | immediate early response 3 interacting protein 1 | | 10.17159343 | 1.49E-17 | cluster P | 1.8 |
| **TUBD1** | tubulin, delta 1 | | 8.34352107 | 1.35E-13 | cluster P | 1.35 |
| **TUBE1** | tubulin, epsilon 1 | | 7.865315713 | 1.61E-12 | cluster P | 1.31 |
| **RAPGEFL1** | Rap guanine nucleotide exchange factor (GEF)-like 1 | | 3.693805734 | 0.000331363 | cluster P | 1.28 |
| **KLF3** | Kruppel-like factor 3 (basic) | | 9.571097421 | 1.48E-16 | cluster P | 1.41 |
| **FKBP11** | FK506 binding protein 11, 19 kDa | | 11.51882922 | 2.19E-20 | cluster P | 2.22 |
| **MEX3C** | mex-3 homolog C (C. elegans) | | 7.656693623 | 1.17E-11 | cluster P | 1.29 |
| **CSAD** | cysteine sulfinic acid decarboxylase | | 4.686188335 | 7.76E-06 | cluster P | 1.31 |
| **NIP7** | NIP7, nucleolar pre-rRNA processing protein | | 7.71702293 | 9.81E-12 | cluster P | 1.5 |
| **TRAPPC4** | trafficking protein particle complex 4 | | 8.449816791 | 2.12E-13 | cluster P | 1.49 |
| **PPME1** | protein phosphatase methylesterase 1 | | 8.95010193 | 1.43E-14 | cluster P | 1.82 |
| **YTHDF2** | YTH domain family, member 2 | | 6.002985325 | 2.72E-08 | cluster P | 1.32 |
| **LIMA1** | LIM domain and actin binding 1 | | 3.675783503 | 0.000368902 | cluster P | 1.3 |
| **CWC15** | CWC15 spliceosome-associated protein homolog (S. cerevisiae) | | 9.217153919 | 1.17E-15 | cluster P | 1.57 |
| **DTL** | denticleless E3 ubiquitin protein ligase homolog (Drosophila) | | 5.044028206 | 1.65E-06 | cluster P | 1.37 |
| **CXXC5** | CXXC finger protein 5 | | 6.322195499 | 4.80E-09 | cluster P | 1.42 |
| **C20orf111** | chromosome 20 open reading frame 111 | | 4.937575826 | 2.57E-06 | cluster P | 1.25 |
| **YPEL5** | yippee-like 5 (Drosophila) | | 9.072012017 | 2.39E-15 | cluster P | 1.72 |
| **ZFR** | zinc finger RNA binding protein | | 10.97861868 | 6.30E-20 | cluster P | 1.72 |
| **CMPK1** | cytidine monophosphate (UMP-CMP) kinase 1, cytosolic | | 7.760992593 | 3.95E-12 | cluster P | 1.46 |
| **ARID4B** | AT rich interactive domain 4B (RBP1-like) | | 5.748354882 | 7.56E-08 | cluster P | 1.29 |
| **C9orf78** | chromosome 9 open reading frame 78 | | 8.698398187 | 3.14E-14 | cluster P | 1.41 |
| **RAB8B** | RAB8B, member RAS oncogene family | | 10.46376772 | 1.59E-16 | cluster P | 2.36 |
| **MST4** | serine/threonine protein kinase MST4 | | 8.626167951 | 6.19E-14 | cluster P | 1.39 |
| **BTBD1** | BTB (POZ) domain containing 1 | | 9.229110747 | 1.47E-15 | cluster P | 1.41 |
| **CSNK1G1** | casein kinase 1, gamma 1 | | 5.572893898 | 1.63E-07 | cluster P | 1.21 |
| **POLE3** | polymerase (DNA directed), epsilon 3, accessory subunit | | 7.503176614 | 7.80E-11 | cluster P | 2.49 |
| **DNAJC10** | DnaJ (Hsp40) homolog, subfamily C, member 10 | | 12.37691144 | 4.18E-22 | cluster P | 2.03 |
| **PLEKHA5** | pleckstrin homology domain containing, family A member 5 | | 8.829407551 | 1.46E-13 | cluster P | 1.61 |
| **DDIT4** | DNA-damage-inducible transcript 4 | | 6.401604646 | 3.15E-09 | cluster P | 1.46 |
| **PAF1** | Paf1, RNA polymerase II associated factor, homolog (S. cerevisiae) | | 11.99772831 | 1.08E-21 | cluster P | 1.72 |
| **TBC1D13** | TBC1 domain family, member 13 | | 8.076633171 | 5.91E-13 | cluster P | 1.87 |
| **RSBN1** | round spermatid basic protein 1 | | 5.407108639 | 3.21E-07 | cluster P | 1.32 |
| **TMED9** | transmembrane emp24 protein transport domain containing 9 | | 7.133717256 | 1.05E-10 | cluster P | 1.44 |
| **KLHDC4** | kelch domain containing 4 | | 11.68799681 | 2.58E-21 | cluster P | 2.06 |
| **KCTD9** | potassium channel tetramerisation domain containing 9 | | 10.20569576 | 2.37E-17 | cluster P | 1.73 |
| **BNC2** | basonuclin 2 | | 5.433249351 | 2.85E-07 | cluster P | 1.28 |
| **GATAD2A** | GATA zinc finger domain containing 2A | | 5.028584947 | 1.96E-06 | cluster P | 1.38 |
| **NDE1** | nudE nuclear distribution E homolog 1 (A. nidulans) | | 13.10420533 | 7.76E-25 | cluster P | 1.72 |
| **NUP62CL** | nucleoporin 62kDa C-terminal like | | 6.539893719 | 1.83E-09 | cluster P | 1.67 |
| **GON4L** | gon-4-like (C. elegans) | | 4.077178423 | 9.66E-05 | cluster P | 1.18 |
| **NSUN2** | NOP2/Sun RNA methyltransferase family, member 2 | | 16.27818793 | 6.91E-32 | cluster P | 1.97 |
| **TTC19** | tetratricopeptide repeat domain 19 | | 11.24956259 | 1.65E-20 | cluster P | 2.28 |
| **DUS2L** | dihydrouridine synthase 2-like, SMM1 homolog (S. cerevisiae) | | 9.858543182 | 3.01E-17 | cluster P | 1.53 |
| **LPCAT2** | lysophosphatidylcholine acyltransferase 2 | | 3.995332525 | 0.000115924 | cluster P | 1.2 |
| **VPS37C** | vacuolar protein sorting 37 homolog C (S. cerevisiae) | | 7.608623867 | 6.24E-11 | cluster P | 1.46 |
| **OXR1** | oxidation resistance 1 | | 12.43958686 | 2.01E-20 | cluster P | 3.31 |
| **ARGLU1** | arginine and glutamate rich 1 | | 9.652197256 | 1.93E-15 | cluster P | 1.96 |
| **NAT10** | N-acetyltransferase 10 (GCN5-related) | | 8.621416647 | 3.34E-14 | cluster P | 1.49 |
| **UBA6** | ubiquitin-like modifier activating enzyme 6 | | 9.043157776 | 2.90E-15 | cluster P | 1.45 |
| **SLC38A7** | solute carrier family 38, member 7 | | 5.704433032 | 8.25E-08 | cluster P | 1.33 |
| **ASXL2** | additional sex combs like 2 (Drosophila) | | 4.582256422 | 1.12E-05 | cluster P | 1.3 |
| **C7orf43** | chromosome 7 open reading frame 43 | | 4.585829215 | 1.13E-05 | cluster P | 1.25 |
| **WDR33** | WD repeat domain 33 | | 8.257693716 | 2.45E-13 | cluster P | 1.49 |
| **SLC22A15** | solute carrier family 22, member 15 | | 9.061925696 | 2.57E-14 | cluster P | 2.3 |
| **LRRC59** | leucine rich repeat containing 59 | | 7.237833107 | 6.46E-11 | cluster P | 1.32 |
| **C17orf85** | chromosome 17 open reading frame 85 | | 3.627508289 | 0.000453014 | cluster P | 1.22 |
| **YOD1** | YOD1 OTU deubiquinating enzyme 1 homolog (S. cerevisiae) | | 5.963442508 | 2.56E-08 | cluster P | 1.37 |
| **HES6** | hairy and enhancer of split 6 (Drosophila) | | 6.780157894 | 7.34E-10 | cluster P | 1.71 |
| **ZNF821** | zinc finger protein 821 | | 6.011287667 | 2.23E-08 | cluster P | 1.53 |
| **UBE2Q1** | ubiquitin-conjugating enzyme E2Q family member 1 | | 8.123154977 | 6.55E-13 | cluster P | 1.57 |
| **RNPC3** | RNA-binding region (RNP1, RRM) containing 3 | | 5.664118574 | 9.90E-08 | cluster P | 1.22 |
| **PPP1R9A** | protein phosphatase 1, regulatory subunit 9A | | 6.165478166 | 1.40E-08 | cluster P | 1.47 |
| **CHD7** | chromodomain helicase DNA binding protein 7 | | 9.814637831 | 3.92E-17 | cluster P | 1.75 |
| **PIGV** | phosphatidylinositol glycan anchor biosynthesis, class V | | 9.104025446 | 1.96E-15 | cluster P | 1.47 |
| **CDC37L1** | cell division cycle 37-like 1 | | 15.23843536 | 1.79E-28 | cluster P | 2.11 |
| **IWS1** | IWS1 homolog (S. cerevisiae) | | 6.497360704 | 2.87E-09 | cluster P | 1.27 |
| **SCYL2** | SCY1-like 2 (S. cerevisiae) | | 12.08061328 | 1.85E-21 | cluster P | 2.05 |
| **FRMD4A** | FERM domain containing 4A | | 9.581914831 | 3.33E-15 | cluster P | 2.66 |
| **FAR2** | fatty acyl CoA reductase 2 | | 6.195479548 | 1.02E-08 | cluster P | 1.5 |
| **VPS35** | vacuolar protein sorting 35 homolog (S. cerevisiae) | | 9.105081178 | 4.53E-14 | cluster P | 2.08 |
| **EDEM2** | ER degradation enhancer, mannosidase alpha-like 2 | | 5.927804967 | 3.84E-08 | cluster P | 1.34 |
| **H2AFJ** | H2A histone family, member J | | 7.603059967 | 1.18E-11 | cluster P | 1.28 |
| **CAND1** | cullin-associated and neddylation-dissociated 1 | | 3.926203407 | 0.000172856 | cluster P | 1.17 |
| **WSB2** | WD repeat and SOCS box containing 2 | | 15.13263566 | 9.76E-30 | cluster P | 2.01 |
| **GNG12** | guanine nucleotide binding protein (G protein), gamma 12 | | 7.529662184 | 1.06E-11 | cluster P | 1.61 |
| **SLC25A40** | solute carrier family 25, member 40 | | 4.270087951 | 3.87E-05 | cluster P | 1.28 |
| **CPXM1** | carboxypeptidase X (M14 family), member 1 | | 5.781211744 | 6.58E-08 | cluster P | 1.3 |
| **EIF4ENIF1** | eukaryotic translation initiation factor 4E nuclear import factor 1 | | 4.310210225 | 3.59E-05 | cluster P | 1.23 |
| **CLDND1** | claudin domain containing 1 | | 4.212725485 | 4.84E-05 | cluster P | 1.18 |
| **POLE4** | polymerase (DNA-directed), epsilon 4, accessory subunit | | 6.262023659 | 6.06E-09 | cluster P | 1.44 |
| **TRIM39** | tripartite motif containing 39 | | 4.894697495 | 4.67E-06 | cluster P | 1.24 |
| **TMEM9B** | TMEM9 domain family, member B | | 10.1884468 | 8.04E-18 | cluster P | 1.68 |
| **SPHK2** | sphingosine kinase 2 | | 8.195013659 | 3.06E-13 | cluster P | 1.83 |
| **PNO1** | partner of NOB1 homolog (S. cerevisiae) | | 11.81686005 | 5.41E-22 | cluster P | 1.59 |
| **C1GALT1** | core 1 synthase, glycoprotein-N-acetylgalactosamine 3-beta-galactosyltransferase, 1 | | 9.369215861 | 1.01E-15 | cluster P | 1.6 |
| **SMARCAD1** | SWI/SNF-related, matrix-associated actin-dependent regulator of chromatin, subfamily a, containing DEAD/H box 1 | | 4.100252696 | 8.36E-05 | cluster P | 1.19 |
| **ENY2** | enhancer of yellow 2 homolog (Drosophila) | | 3.787794594 | 0.000271638 | cluster P | 1.2 |
| **PRDM8** | PR domain containing 8 | | 6.113009492 | 1.20E-08 | cluster P | 1.52 |
| **PLSCR3** | phospholipid scramblase 3 | | 11.16122894 | 2.09E-20 | cluster P | 2.36 |
| **ENTPD7** | ectonucleoside triphosphate diphosphohydrolase 7 | | 13.5815062 | 4.79E-26 | cluster P | 1.53 |
| **MRPL47** | mitochondrial ribosomal protein L47 | | 7.705652614 | 3.78E-12 | cluster P | 1.38 |
| **LYRM1** | LYR motif containing 1 | | 8.877041446 | 1.24E-14 | cluster P | 1.67 |
| **SNX14** | sorting nexin 14 | | 5.073560844 | 1.58E-06 | cluster P | 1.22 |
| **BIRC6** | baculoviral IAP repeat containing 6 | | 13.55751542 | 8.35E-26 | cluster P | 1.61 |
| **AARS2** | alanyl-tRNA synthetase 2, mitochondrial | | 7.906267831 | 1.54E-12 | cluster P | 1.31 |
| **NUFIP2** | nuclear fragile X mental retardation protein interacting protein 2 | | 11.89473967 | 3.48E-22 | cluster P | 2.03 |
| **WDFY1** | WD repeat and FYVE domain containing 1 | | 19.30408654 | 7.92E-39 | cluster P | 2.6 |
| **TSHZ3** | teashirt zinc finger homeobox 3 | | 13.39041038 | 3.62E-23 | cluster P | 2.37 |
| **KIAA1598** | KIAA1598 | | 4.848614533 | 3.82E-06 | cluster P | 1.31 |
| **MIER1** | mesoderm induction early response 1 homolog (Xenopus laevis) | | 10.40366375 | 2.16E-18 | cluster P | 1.51 |
| **RAP2C** | RAP2C, member of RAS oncogene family | | 5.085050713 | 1.82E-06 | cluster P | 1.22 |
| **ZNF410** | zinc finger protein 410 | | 9.814568036 | 4.29E-17 | cluster P | 1.62 |
| **SCAF1** | SR-related CTD-associated factor 1 | | 8.91116936 | 1.63E-14 | cluster P | 1.61 |
| **RBM25** | RNA binding motif protein 25 | | 13.46529713 | 7.89E-26 | cluster P | 1.74 |
| **MID1IP1** | MID1 interacting protein 1 | | 7.26195468 | 5.08E-11 | cluster P | 1.52 |
| **GPBP1L1** | GC-rich promoter binding protein 1-like 1 | | 4.319105562 | 3.47E-05 | cluster P | 1.13 |
| **RNF123** | ring finger protein 123 | | 9.860598031 | 3.12E-17 | cluster P | 1.8 |
| **PERP** | PERP, TP53 apoptosis effector | | 8.420725278 | 8.14E-14 | cluster P | 1.29 |
| **RBKS** | ribokinase | | 3.794538988 | 0.000231081 | cluster P | 1.15 |
| **GOLPH3** | golgi phosphoprotein 3 (coat-protein) | | 10.1137284 | 3.15E-17 | cluster P | 1.81 |
| **XYLT2** | xylosyltransferase II | | 11.46556995 | 3.84E-21 | cluster P | 1.72 |
| **PDF** | peptide deformylase (mitochondrial) | | 7.692580139 | 5.64E-12 | cluster P | 1.5 |
| **NOC3L** | nucleolar complex associated 3 homolog (S. cerevisiae) | | 10.3353895 | 9.21E-17 | cluster P | 2.3 |
| **NSD1** | nuclear receptor binding SET domain protein 1 | | 8.496981441 | 6.64E-14 | cluster P | 1.43 |
| **RFWD2** | ring finger and WD repeat domain 2, E3 ubiquitin protein ligase | | 11.07236934 | 8.19E-19 | cluster P | 1.99 |
| **XPO4** | exportin 4 | | 11.95414861 | 2.89E-21 | cluster P | 2.03 |
| **ARHGAP9** | Rho GTPase activating protein 9 | | 4.292547561 | 3.58E-05 | cluster P | 1.21 |
| **IKZF5** | IKAROS family zinc finger 5 (Pegasus) | | 9.212957708 | 1.15E-15 | cluster P | 1.66 |
| **AKTIP** | AKT interacting protein | | 6.701339786 | 7.10E-10 | cluster P | 1.41 |
| **HIAT1** | hippocampus abundant transcript 1 | | 6.785169944 | 4.89E-10 | cluster P | 1.19 |
| **MFSD1** | major facilitator superfamily domain containing 1 | | 10.1762363 | 8.46E-18 | cluster P | 1.69 |
| **S100PBP** | S100P binding protein | | 8.016454718 | 1.13E-12 | cluster P | 1.31 |
| **SPATS2** | spermatogenesis associated, serine-rich 2 | | 8.488053313 | 5.72E-14 | cluster P | 1.41 |
| **C7orf25** | chromosome 7 open reading frame 25 | | 3.783136411 | 0.000269272 | cluster P | 1.18 |
| **KCTD15** | potassium channel tetramerisation domain containing 15 | | 7.583612893 | 7.54E-12 | cluster P | 1.46 |
| **C1orf50** | chromosome 1 open reading frame 50 | | 13.95983815 | 4.00E-26 | cluster P | 2.06 |
| **PHF23** | PHD finger protein 23 | | 12.28421206 | 5.06E-23 | cluster P | 1.68 |
| **CRELD2** | cysteine-rich with EGF-like domains 2 | | 8.537065988 | 2.50E-13 | cluster P | 1.67 |
| **PANK3** | pantothenate kinase 3 | | 4.583857011 | 1.22E-05 | cluster P | 1.19 |
| **HSPBAP1** | HSPB (heat shock 27kDa) associated protein 1 | | 12.67826234 | 2.52E-23 | cluster P | 1.99 |
| **NLRX1** | NLR family member X1 | | 3.399402043 | 0.000934206 | cluster P | 1.31 |
| **ZDHHC14** | zinc finger, DHHC-type containing 14 | | 5.63019215 | 1.21E-07 | cluster P | 1.42 |
| **NPEPL1** | aminopeptidase-like 1 | | 7.092282938 | 1.24E-10 | cluster P | 1.39 |
| **TMEM180** | transmembrane protein 180 | | 5.648593184 | 1.10E-07 | cluster P | 1.42 |
| **HDAC11** | histone deacetylase 11 | | 6.359167785 | 4.35E-09 | cluster P | 1.28 |
| **LPCAT1** | lysophosphatidylcholine acyltransferase 1 | | 3.983491856 | 0.000136056 | cluster P | 1.18 |
| **RPP21** | ribonuclease P/MRP 21kDa subunit | | 12.09694886 | 2.24E-22 | cluster P | 2.13 |
| **MRM1** | mitochondrial rRNA methyltransferase 1 homolog (S. cerevisiae) | | 4.782157439 | 4.97E-06 | cluster P | 1.24 |
| **PLEKHH3** | pleckstrin homology domain containing, family H (with MyTH4 domain) member 3 | | 10.48137361 | 1.63E-18 | cluster P | 1.41 |
| **ELOVL7** | ELOVL fatty acid elongase 7 | | 3.451683646 | 0.000766201 | cluster P | 1.21 |
| **VCPIP1** | valosin containing protein (p97)/p47 complex interacting protein 1 | | 7.43265076 | 1.90E-11 | cluster P | 1.26 |
| **THOC7** | THO complex 7 homolog (Drosophila) | | 5.941351475 | 2.72E-08 | cluster P | 1.41 |
| **IFT74** | intraflagellar transport 74 homolog (Chlamydomonas) | | 8.187716789 | 2.91E-13 | cluster P | 1.56 |
| **MUS81** | MUS81 endonuclease homolog (S. cerevisiae) | | 7.30613471 | 3.05E-11 | cluster P | 1.27 |
| **FBXO11** | F-box protein 11 | | 5.284600201 | 6.77E-07 | cluster P | 1.25 |
| **EDEM3** | ER degradation enhancer, mannosidase alpha-like 3 | | 3.953887844 | 0.000140854 | cluster P | 1.13 |
| **CYB5B** | cytochrome b5 type B (outer mitochondrial membrane) | | 11.84780532 | 4.20E-21 | cluster P | 1.97 |
| **ZFP91** | ZFP91 zinc finger protein | | 9.010104255 | 3.47E-14 | cluster P | 1.39 |
| **ITFG1** | integrin alpha FG-GAP repeat containing 1 | | 7.201369983 | 5.50E-11 | cluster P | 1.23 |
| **APOLD1** | apolipoprotein L domain containing 1 | | 5.623580387 | 1.23E-07 | cluster P | 1.56 |
| **TRIM8** | tripartite motif containing 8 | | 5.29232955 | 5.84E-07 | cluster P | 1.34 |
| **URM1** | ubiquitin related modifier 1 | | 8.662946704 | 8.33E-14 | cluster P | 1.99 |
| **C6orf62** | chromosome 6 open reading frame 62 | | 12.58629929 | 7.47E-24 | cluster P | 1.97 |
| **SLC25A28** | solute carrier family 25 (mitochondrial iron transporter), member 28 | | 10.35217654 | 2.06E-17 | cluster P | 1.61 |
| **COG3** | component of oligomeric golgi complex 3 | | 7.198463455 | 1.78E-10 | cluster P | 1.3 |
| **INHBE** | inhibin, beta E | | 4.073334249 | 8.24E-05 | cluster P | 1.23 |
| **ITCH** | itchy E3 ubiquitin protein ligase | | 12.51706543 | 1.17E-23 | cluster P | 1.95 |
| **GRWD1** | glutamate-rich WD repeat containing 1 | | 6.840027353 | 7.28E-10 | cluster P | 1.45 |
| **SPNS1** | spinster homolog 1 (Drosophila) | | 7.782545324 | 2.75E-12 | cluster P | 1.33 |
| **NCALD** | neurocalcin delta | | 5.345990415 | 4.50E-07 | cluster P | 1.53 |
| **ZRANB3** | zinc finger, RAN-binding domain containing 3 | | 3.714490043 | 0.000326484 | cluster P | 1.14 |
| **TMEM164** | transmembrane protein 164 | | 4.055277498 | 9.52E-05 | cluster P | 1.16 |
| **RHBDD1** | rhomboid domain containing 1 | | 7.671479762 | 6.06E-12 | cluster P | 1.55 |
| **DCUN1D5** | DCN1, defective in cullin neddylation 1, domain containing 5 (S. cerevisiae) | | 6.672182402 | 2.38E-09 | cluster P | 1.63 |
| **FBXW9** | F-box and WD repeat domain containing 9 | | 7.748518941 | 3.01E-12 | cluster P | 1.33 |
| **WIBG** | within bgcn homolog (Drosophila) | | 12.64251976 | 2.54E-23 | cluster P | 1.9 |
| **C7orf50** | chromosome 7 open reading frame 50 | | 7.823104157 | 1.94E-11 | cluster P | 1.39 |
| **SYVN1** | synovial apoptosis inhibitor 1, synoviolin | | 4.886385554 | 3.46E-06 | cluster P | 1.24 |
| **ZMAT1** | zinc finger, matrin-type 1 | | 4.207886542 | 5.08E-05 | cluster P | 1.33 |
| **BEX2** | brain expressed X-linked 2 | | 6.243810262 | 1.88E-08 | cluster P | 1.98 |
| **PCGF1** | polycomb group ring finger 1 | | 7.18956017 | 5.65E-11 | cluster P | 1.3 |
| **TUBA1C** | tubulin, alpha 1c | | 8.417990436 | 9.09E-14 | cluster P | 1.68 |
| **AGPAT9** | 1-acylglycerol-3-phosphate O-acyltransferase 9 | | 8.504469249 | 1.58E-13 | cluster P | 1.45 |
| **TXNDC17** | thioredoxin domain containing 17 | | 14.37425435 | 3.28E-26 | cluster P | 2.67 |
| **ADO** | 2-aminoethanethiol (cysteamine) dioxygenase | | 10.47723414 | 6.98E-18 | cluster P | 1.71 |
| **SLC35B4** | solute carrier family 35, member B4 | | 9.065792845 | 2.66E-15 | cluster P | 1.57 |
| **PPP1R15B** | protein phosphatase 1, regulatory subunit 15B | | 9.79175242 | 5.08E-17 | cluster P | 1.41 |
| **FIZ1** | FLT3-interacting zinc finger 1 | | 6.433672496 | 2.73E-09 | cluster P | 1.57 |
| **SPRYD3** | SPRY domain containing 3 | | 13.90623295 | 9.53E-26 | cluster P | 2.63 |
| **RAB2B** | RAB2B, member RAS oncogene family | | 9.80059664 | 2.21E-16 | cluster P | 2.26 |
| **C12orf52** | chromosome 12 open reading frame 52 | | 11.34028415 | 8.78E-20 | cluster P | 2.01 |
| **MUM1** | melanoma associated antigen (mutated) 1 | | 8.935436274 | 5.08E-15 | cluster P | 1.5 |
| **TCEAL3** | transcription elongation factor A (SII)-like 3 | | 6.000577813 | 2.20E-08 | cluster P | 1.46 |
| **HIST1H2AH** | histone cluster 1, H2ah | | 4.390170668 | 2.48E-05 | cluster P | 1.46 |
| **MIDN** | midnolin | | 12.78996442 | 8.92E-24 | cluster P | 2.26 |
| **PHLDB2** | pleckstrin homology-like domain, family B, member 2 | | 8.230887331 | 3.01E-13 | cluster P | 1.69 |
| **FAM105B** | family with sequence similarity 105, member B | | 8.821719679 | 9.48E-15 | cluster P | 1.6 |
| **MCFD2** | multiple coagulation factor deficiency 2 | | 7.585793876 | 8.31E-12 | cluster P | 1.48 |
| **ZNF622** | zinc finger protein 622 | | 14.54880248 | 1.67E-28 | cluster P | 2 |
| **GADD45GIP1** | growth arrest and DNA-damage-inducible, gamma interacting protein 1 | | 12.11616231 | 1.08E-22 | cluster P | 1.55 |
| **SEC11C** | SEC11 homolog C (S. cerevisiae) | | 5.784114766 | 9.09E-08 | cluster P | 1.2 |
| **TCEAL8** | transcription elongation factor A (SII)-like 8 | | 12.77525883 | 2.09E-21 | cluster P | 1.82 |
| **TRIM47** | tripartite motif containing 47 | | 7.806462291 | 2.91E-12 | cluster P | 1.61 |
| **SLC25A46** | solute carrier family 25, member 46 | | 9.414399718 | 1.36E-15 | cluster P | 1.74 |
| **YTHDC1** | YTH domain containing 1 | | 9.072581097 | 1.43E-14 | cluster P | 1.75 |
| **MTDH** | metadherin | | 5.160219576 | 9.80E-07 | cluster P | 1.21 |
| **MRRF** | mitochondrial ribosome recycling factor | | 6.08509796 | 1.38E-08 | cluster P | 1.3 |
| **SPECC1** | sperm antigen with calponin homology and coiled-coil domains 1 | | 7.080167112 | 9.82E-11 | cluster P | 1.34 |
| **CCDC64** | coiled-coil domain containing 64 | | 5.751600894 | 6.63E-08 | cluster P | 1.39 |
| **SHKBP1** | SH3KBP1 binding protein 1 | | 5.003312165 | 1.91E-06 | cluster P | 1.3 |
| **HNRPLL** | heterogeneous nuclear ribonucleoprotein L-like | | 7.762832368 | 2.95E-12 | cluster P | 1.44 |
| **UBE2Q2** | ubiquitin-conjugating enzyme E2Q family member 2 | | 7.8094923 | 1.60E-11 | cluster P | 1.72 |
| **ORAI3** | ORAI calcium release-activated calcium modulator 3 | | 6.05290197 | 2.18E-08 | cluster P | 1.35 |
| **TJAP1** | tight junction associated protein 1 (peripheral) | | 11.31040411 | 2.91E-20 | cluster P | 1.94 |
| **CADPS2** | Ca++-dependent secretion activator 2 | | 7.822360577 | 3.04E-12 | cluster P | 1.41 |
| **ATPIF1** | ATPase inhibitory factor 1 | | 6.237534953 | 9.75E-09 | cluster P | 1.22 |
| **EGLN3** | egl nine homolog 3 (C. elegans) | | 7.535811601 | 4.08E-11 | cluster P | 1.88 |
| **C1orf85** | chromosome 1 open reading frame 85 | | 14.03857653 | 7.51E-25 | cluster P | 2.59 |
| **TEX261** | testis expressed 261 | | 7.452929952 | 3.03E-11 | cluster P | 1.74 |
| **DTX2** | deltex homolog 2 (Drosophila) | | 8.360072002 | 2.66E-13 | cluster P | 1.7 |
| **MRFAP1L1** | Morf4 family associated protein 1-like 1 | | 7.873375043 | 1.56E-12 | cluster P | 1.67 |
| **KCTD12** | potassium channel tetramerisation domain containing 12 | | 9.443797408 | 4.72E-15 | cluster P | 1.72 |
| **CTHRC1** | collagen triple helix repeat containing 1 | | 9.677297654 | 1.03E-16 | cluster P | 1.44 |
| **KLHDC3** | kelch domain containing 3 | | 6.940536188 | 1.08E-09 | cluster P | 1.35 |
| **AEBP2** | AE binding protein 2 | | 3.500138677 | 0.000652299 | cluster P | 1.1 |
| **TC2N** | tandem C2 domains, nuclear | | 6.761922088 | 7.67E-10 | cluster P | 1.26 |
| **SLC38A10** | solute carrier family 38, member 10 | | 4.797836125 | 4.96E-06 | cluster P | 1.31 |
| **SLC43A2** | solute carrier family 43, member 2 | | 6.026010699 | 1.87E-08 | cluster P | 1.48 |
| **C17orf49** | chromosome 17 open reading frame 49 | | 7.42399119 | 1.90E-11 | cluster P | 1.69 |
| **MRPL10** | mitochondrial ribosomal protein L10 | | 5.866000443 | 3.94E-08 | cluster P | 1.27 |
| **NR2C2AP** | nuclear receptor 2C2-associated protein | | 11.02033911 | 5.91E-20 | cluster P | 1.84 |
| **UHMK1** | U2AF homology motif (UHM) kinase 1 | | 11.42634312 | 1.83E-20 | cluster P | 2.06 |
| **TBC1D20** | TBC1 domain family, member 20 | | 9.809754674 | 4.04E-17 | cluster P | 1.63 |
| **PIGU** | phosphatidylinositol glycan anchor biosynthesis, class U | | 4.553734161 | 2.14E-05 | cluster P | 1.19 |
| **ZFAND2B** | zinc finger, AN1-type domain 2B | | 9.492865199 | 2.68E-16 | cluster P | 1.73 |
| **AHSA2** | AHA1, activator of heat shock 90kDa protein ATPase homolog 2 (yeast) | | 7.24327037 | 6.83E-11 | cluster P | 1.33 |
| **DCBLD2** | discoidin, CUB and LCCL domain containing 2 | | 5.961757045 | 7.66E-08 | cluster P | 2.27 |
| **LIN54** | lin-54 homolog (C. elegans) | | 4.76885726 | 5.59E-06 | cluster P | 1.17 |
| **STXBP5** | syntaxin binding protein 5 (tomosyn) | | 3.983088151 | 0.000127689 | cluster P | 1.31 |
| **NCOA7** | nuclear receptor coactivator 7 | | 4.807096209 | 4.38E-06 | cluster P | 1.44 |
| **MTPN** | myotrophin | | 13.46531722 | 7.04E-23 | cluster P | 3.18 |
| **UPRT** | uracil phosphoribosyltransferase (FUR1) homolog (S. cerevisiae) | | 13.92376805 | 4.18E-26 | cluster P | 2.14 |
| **SPIN4** | spindlin family, member 4 | | 12.38223986 | 3.04E-21 | cluster P | 2.54 |
| **ASB6** | ankyrin repeat and SOCS box containing 6 | | 3.53331252 | 0.000608258 | cluster P | 1.16 |
| **CWF19L2** | CWF19-like 2, cell cycle control (S. pombe) | | 4.802480982 | 5.17E-06 | cluster P | 1.29 |
| **ZNF664** | zinc finger protein 664 | | 6.211795255 | 8.76E-09 | cluster P | 1.3 |
| **ZFP90** | ZFP90 zinc finger protein | | 11.94271815 | 1.00E-20 | cluster P | 1.95 |
| **ZNF785** | zinc finger protein 785 | | 4.754660706 | 6.25E-06 | cluster P | 1.15 |
| **SIX5** | SIX homeobox 5 | | 12.79458192 | 2.89E-23 | cluster P | 2.14 |
| **CDC42EP5** | CDC42 effector protein (Rho GTPase binding) 5 | | 3.987074569 | 0.000117623 | cluster P | 1.43 |
| **C1orf52** | chromosome 1 open reading frame 52 | | 13.96957355 | 2.40E-25 | cluster P | 2.2 |
| **SLC30A7** | solute carrier family 30 (zinc transporter), member 7 | | 7.658353411 | 1.04E-11 | cluster P | 1.43 |
| **ZNF362** | zinc finger protein 362 | | 8.178043709 | 3.45E-13 | cluster P | 1.84 |
| **PDIK1L** | PDLIM1 interacting kinase 1 like | | 7.890517978 | 1.49E-12 | cluster P | 1.49 |
| **SRFBP1** | serum response factor binding protein 1 | | 9.340463258 | 8.07E-16 | cluster P | 1.77 |
| **ZMAT2** | zinc finger, matrin-type 2 | | 6.970520073 | 4.76E-10 | cluster P | 1.35 |
| **VKORC1L1** | vitamin K epoxide reductase complex, subunit 1-like 1 | | 5.039253425 | 1.64E-06 | cluster P | 1.37 |
| **RDH10** | retinol dehydrogenase 10 (all-trans) | | 9.57167512 | 4.97E-16 | cluster P | 1.97 |
| **SASS6** | spindle assembly 6 homolog (C. elegans) | | 8.073132683 | 6.92E-13 | cluster P | 1.6 |
| **FAM76A** | family with sequence similarity 76, member A | | 6.496806281 | 3.78E-09 | cluster P | 1.73 |
| **KCTD6** | potassium channel tetramerisation domain containing 6 | | 7.96418319 | 2.41E-12 | cluster P | 1.52 |
| **ARL13B** | ADP-ribosylation factor-like 13B | | 11.94287832 | 4.71E-22 | cluster P | 1.47 |
| **STT3B** | STT3B, subunit of the oligosaccharyltransferase complex (catalytic) | | 7.404070738 | 2.44E-11 | cluster P | 1.5 |
| **PDE12** | phosphodiesterase 12 | | 7.022031704 | 1.54E-10 | cluster P | 1.32 |
| **ANKS6** | ankyrin repeat and sterile alpha motif domain containing 6 | | 3.840393156 | 0.000202054 | cluster P | 1.15 |
| **C3orf58** | chromosome 3 open reading frame 58 | | 10.45942882 | 1.52E-18 | cluster P | 1.77 |
| **SLC36A1** | solute carrier family 36 (proton/amino acid symporter), member 1 | | 4.379326576 | 3.07E-05 | cluster P | 1.35 |
| **LEMD2** | LEM domain containing 2 | | 7.884492633 | 3.14E-12 | cluster P | 1.52 |
| **C7orf41** | chromosome 7 open reading frame 41 | | 8.508139683 | 1.81E-13 | cluster P | 2.13 |
| **BRWD3** | bromodomain and WD repeat domain containing 3 | | 8.787640732 | 1.23E-14 | cluster P | 1.41 |
| **ELMOD2** | ELMO/CED-12 domain containing 2 | | 7.503106196 | 1.09E-11 | cluster P | 1.41 |
| **RNF214** | ring finger protein 214 | | 10.15260572 | 7.65E-18 | cluster P | 1.39 |
| **TIPRL** | TIP41, TOR signaling pathway regulator-like (S. cerevisiae) | | 4.245337931 | 5.23E-05 | cluster P | 1.17 |
| **EFCAB4A** | EF-hand calcium binding domain 4A | | 3.65550709 | 0.000380319 | cluster P | 1.38 |
| **RASSF3** | Ras association (RalGDS/AF-6) domain family member 3 | | 4.743669841 | 5.72E-06 | cluster P | 1.32 |
| **RNF149** | ring finger protein 149 | | 5.304086283 | 5.72E-07 | cluster P | 1.24 |
| **SUMF1** | sulfatase modifying factor 1 | | 7.142649689 | 7.91E-11 | cluster P | 1.52 |
| **FRYL** | FRY-like | | 6.862988585 | 2.93E-10 | cluster P | 1.23 |
| **DCBLD1** | discoidin, CUB and LCCL domain containing 1 | | 8.4200638 | 9.08E-14 | cluster P | 1.65 |
| **SLC35B2** | solute carrier family 35, member B2 | | 4.45248145 | 1.89E-05 | cluster P | 1.4 |
| **LRRC37A3** | leucine rich repeat containing 37, member A3 | | 3.694573801 | 0.000334859 | cluster P | 1.31 |
| **NOTCH2NL** | notch 2 N-terminal like | | 9.469554282 | 4.27E-16 | cluster P | 1.59 |
| **BOLA3** | bolA homolog 3 (E. coli) | | 4.404503998 | 2.47E-05 | cluster P | 1.53 |
| **CUEDC1** | CUE domain containing 1 | | 9.963891776 | 2.93E-16 | cluster P | 2.37 |
| **DNLZ** | DNL-type zinc finger | | 6.656577463 | 1.49E-09 | cluster P | 1.48 |
| **DENND6A** | DENN/MADD domain containing 6A | | 7.031814873 | 1.44E-10 | cluster P | 1.25 |
| **HNRNPR** | heterogeneous nuclear ribonucleoprotein R | | 5.97259308 | 2.40E-08 | cluster P | 1.36 |
| **EMC1** | ER membrane protein complex subunit 1 | | 12.40747859 | 4.01E-23 | cluster P | 2.07 |
| **PDP1** | pyruvate dehyrogenase phosphatase catalytic subunit 1 | | 4.960925357 | 2.30E-06 | cluster P | 1.21 |
| **CAMSAP2** | calmodulin regulated spectrin-associated protein family, member 2 | | 9.533696765 | 5.80E-15 | cluster P | 2.15 |
| **DRAM2** | DNA-damage regulated autophagy modulator 2 | | 8.752259458 | 2.30E-14 | cluster P | 1.48 |
| **PAXBP1** | PAX3 and PAX7 binding protein 1 | | 8.347810559 | 2.42E-13 | cluster P | 1.57 |
| **IRF2BPL** | interferon regulatory factor 2 binding protein-like | | 10.94558341 | 1.34E-19 | cluster P | 1.65 |
| **FAM219A** | family with sequence similarity 219, member A | | 5.010195153 | 1.88E-06 | cluster P | 1.18 |
| **KDM5B** | lysine (K)-specific demethylase 5B | | 7.108980905 | 1.18E-10 | cluster P | 1.48 |
| **NOP58** | NOP58 ribonucleoprotein | | 6.148313671 | 2.63E-08 | cluster P | 1.28 |
| **UBE2Q2P1** | ubiquitin-conjugating enzyme E2Q family member 2 pseudogene 1 | | 3.665527478 | 0.000408032 | cluster P | 1.14 |
| **WDR83** | WD repeat domain 83 | | 5.090622307 | 1.41E-06 | cluster P | 1.38 |
| **ZNF777** | zinc finger protein 777 | | 4.433797721 | 2.59E-05 | cluster P | 1.13 |
| **ATL1** | atlastin GTPase 1 | | 4.539220649 | 1.45E-05 | cluster P | 1.31 |
| **CES4A** | carboxylesterase 4A | | 5.688795305 | 9.12E-08 | cluster P | 1.27 |
| **H3F3AP4** | H3 histone, family 3A, pseudogene 4 | | 9.281863243 | 8.89E-16 | cluster P | 1.72 |
| **N4BP2L2** | NEDD4 binding protein 2-like 2 | | 5.579656115 | 1.71E-07 | cluster P | 1.49 |
| **ORC4** | origin recognition complex, subunit 4 | | 3.775357117 | 0.000248211 | cluster P | 1.21 |
| **ORC5** | origin recognition complex, subunit 5 | | 6.261774033 | 6.25E-09 | cluster P | 1.4 |
| **RNF115** | ring finger protein 115 | | 13.78734564 | 5.57E-26 | cluster P | 2.54 |
| **URGCP** | upregulator of cell proliferation | | 7.902463773 | 1.33E-12 | cluster P | 1.44 |
| **ANAPC16** | anaphase promoting complex subunit 16 | | 11.45176299 | 1.56E-19 | cluster P | 1.79 |
| **CMC2** | COX assembly mitochondrial protein 2 homolog (S. cerevisiae) | | 14.60501291 | 1.37E-28 | cluster P | 2.51 |
| **TMEM256** | transmembrane protein 256 | | 15.69280903 | 1.94E-30 | cluster P | 2.64 |
| **ESYT2** | extended synaptotagmin-like protein 2 | | 7.613392726 | 6.27E-12 | cluster P | 2.1 |
| **FAM117B** | family with sequence similarity 117, member B | | 10.77231419 | 2.13E-19 | cluster P | 1.95 |
| **KANSL1** | KAT8 regulatory NSL complex subunit 1 | | 13.00050367 | 5.77E-24 | cluster P | 1.83 |
| **MOK** | MOK protein kinase | | 6.960121531 | 2.10E-10 | cluster P | 1.53 |
| **PI4KA** | phosphatidylinositol 4-kinase, catalytic, alpha | | 11.45530971 | 1.00E-19 | cluster P | 1.82 |
| **PRMT10** | protein arginine methyltransferase 10 (putative) | | 6.097356327 | 1.56E-08 | cluster P | 1.47 |
| **RALGAPA1** | Ral GTPase activating protein, alpha subunit 1 (catalytic) | | 11.92103619 | 3.08E-22 | cluster P | 2.06 |
| **RBM47** | RNA binding motif protein 47 | | 9.189288927 | 2.16E-14 | cluster P | 2.53 |
| **2-Sep** | septin 2 | | 12.25756296 | 7.68E-22 | cluster P | 2.12 |
| **SZRD1** | SUZ RNA binding domain containing 1 | | 7.171538962 | 9.72E-11 | cluster P | 1.48 |
| **CYTH1** | cytohesin 1 | | 4.365447896 | 2.91E-05 | cluster P | 1.27 |
| **SRSF2** | serine/arginine-rich splicing factor 2 | | 9.923449885 | 3.13E-17 | cluster P | 1.41 |
| **LAMTOR1** | late endosomal/lysosomal adaptor, MAPK and MTOR activator 1 | | 8.502170119 | 5.65E-14 | cluster P | 1.85 |
| **NAA15** | N(alpha)-acetyltransferase 15, NatA auxiliary subunit | | 9.645203915 | 1.09E-16 | cluster P | 1.71 |
| **9-Sep** | septin 9 | | 10.62638076 | 2.92E-16 | cluster P | 1.69 |
| **NACC2** | NACC family member 2, BEN and BTB (POZ) domain containing | | 6.893456586 | 2.53E-10 | cluster P | 1.52 |
| **KANSL2** | KAT8 regulatory NSL complex subunit 2 | | 10.88904622 | 9.63E-20 | cluster P | 1.94 |
| **CPED1** | cadherin-like and PC-esterase domain containing 1 | | 7.869859492 | 1.61E-12 | cluster P | 1.87 |
| **DCAF7** | DDB1 and CUL4 associated factor 7 | | 5.639027049 | 1.36E-07 | cluster P | 1.34 |
| **HNRNPUL1** | heterogeneous nuclear ribonucleoprotein U-like 1 | | 9.689341986 | 9.45E-17 | cluster P | 1.89 |
| **MTPAP** | mitochondrial poly(A) polymerase | | 7.57576065 | 7.41E-12 | cluster P | 1.47 |
| **MYL12A** | myosin, light chain 12A, regulatory, non-sarcomeric | | 11.79081851 | 6.05E-19 | cluster P | 1.82 |
| **XXYLT1** | xyloside xylosyltransferase 1 | | 7.712493593 | 4.45E-12 | cluster P | 1.55 |
| **SLC35F6** | solute carrier family 35, member F6 | | 4.441255688 | 1.98E-05 | cluster P | 1.29 |
| **LGALSL** | lectin, galactoside-binding-like | | 8.257823609 | 2.08E-13 | cluster P | 1.38 |
| **2-Mar** | mitochondrial amidoxime reducing component 2 | | 8.611726947 | 2.90E-14 | cluster P | 1.5 |
| **TRAPPC8** | trafficking protein particle complex 8 | | 8.579474572 | 2.76E-13 | cluster P | 1.61 |
| **ARHGEF35** | Rho guanine nucleotide exchange factor (GEF) 35 | | 6.328529651 | 5.48E-09 | cluster P | 1.8 |
| **PCED1A** | PC-esterase domain containing 1A | | 3.519288681 | 0.000641314 | cluster P | 1.16 |
| **PGAP2** | post-GPI attachment to proteins 2 | | 11.54518541 | 3.85E-21 | cluster P | 1.5 |
| **RNASEK** | ribonuclease, RNase K | | 9.256818366 | 4.32E-15 | cluster P | 2.21 |
| **SUGP2** | SURP and G patch domain containing 2 | | 5.811029155 | 5.27E-08 | cluster P | 1.26 |
| **BRIX1** | BRX1, biogenesis of ribosomes, homolog (S. cerevisiae) | | 14.01866925 | 3.93E-27 | cluster P | 1.85 |
| **NAA50** | N(alpha)-acetyltransferase 50, NatE catalytic subunit | | 7.081416172 | 1.05E-10 | cluster P | 1.27 |
| **RSL24D1** | ribosomal L24 domain containing 1 | | 15.27352531 | 1.82E-27 | cluster P | 2.47 |
| **TPGS2** | tubulin polyglutamylase complex subunit 2 | | 8.12267259 | 6.47E-13 | cluster P | 1.42 |
| **SNRNP27** | small nuclear ribonucleoprotein 27kDa (U4/U6.U5) | | 7.290923831 | 3.55E-11 | cluster P | 1.33 |
| **TADA1** | transcriptional adaptor 1 | | 8.943083702 | 4.90E-15 | cluster P | 1.46 |
| **RHNO1** | RAD9-HUS1-RAD1 interacting nuclear orphan 1 | | 10.03935088 | 1.10E-17 | cluster P | 1.78 |
| **MGME1** | mitochondrial genome maintenance exonuclease 1 | | 12.3568243 | 3.86E-23 | cluster P | 2.28 |
| **CELF1** | CUGBP, Elav-like family member 1 | | 7.85270639 | 1.74E-12 | cluster P | 1.53 |
| **HNRNPA1P10** | heterogeneous nuclear ribonucleoprotein A1 pseudogene 10 | | 10.26221017 | 3.68E-17 | cluster P | 1.57 |
| **SRSF9** | serine/arginine-rich splicing factor 9 | | 5.144650993 | 1.04E-06 | cluster P | 1.31 |
| **LUC7L3** | LUC7-like 3 (S. cerevisiae) | | 12.64791718 | 9.02E-24 | cluster P | 1.94 |
| **WAPAL** | wings apart-like homolog (Drosophila) | | 6.258345702 | 8.37E-09 | cluster P | 1.28 |
| **SUN2** | Sad1 and UNC84 domain containing 2 | | 6.951021401 | 7.87E-10 | cluster P | 1.56 |
| **FAM214B** | family with sequence similarity 214, member B | | 8.884947844 | 1.45E-14 | cluster P | 1.78 |
| **CCZ1B** | CCZ1 vacuolar protein trafficking and biogenesis associated homolog B (S. cerevisiae) | | 14.16038498 | 1.25E-25 | cluster P | 2.87 |
| **CEP44** | centrosomal protein 44kDa | | 9.648986034 | 1.25E-16 | cluster P | 1.54 |
| **10-Sep** | septin 10 | | 10.98406176 | 7.44E-20 | cluster P | 1.76 |
| **7-Sep** | septin 7 | | 9.04970456 | 2.74E-15 | cluster P | 1.49 |
| **TRA2B** | transformer 2 beta homolog (Drosophila) | | 10.88521178 | 2.22E-18 | cluster P | 2.18 |
| **CEP95** | centrosomal protein 95kDa | | 9.505559735 | 2.35E-16 | cluster P | 1.59 |
| **HSPA13** | heat shock protein 70kDa family, member 13 | | 10.14131131 | 1.08E-15 | cluster P | 2.31 |
| **NT5C3A** | 5'-nucleotidase, cytosolic IIIA | | 8.434413275 | 1.59E-13 | cluster P | 1.81 |
| **RNF144A** | ring finger protein 144A | | 4.652284864 | 8.61E-06 | cluster P | 1.31 |
| **SMCO4** | single-pass membrane protein with coiled-coil domains 4 | | 7.072635663 | 1.01E-10 | cluster P | 1.26 |
| **ITPRIPL2** | inositol 1,4,5-trisphosphate receptor interacting protein-like 2 | | 4.779337904 | 5.24E-06 | cluster P | 1.3 |
| **ERP44** | endoplasmic reticulum protein 44 | | 9.115543467 | 2.26E-15 | cluster P | 1.45 |
| **GABPB1** | GA binding protein transcription factor, beta subunit 1 | | 6.645725773 | 1.61E-09 | cluster P | 1.22 |
| **MED1** | mediator complex subunit 1 | | 3.426191605 | 0.000901775 | cluster P | 1.14 |
| **CWC22** | CWC22 spliceosome-associated protein homolog (S. cerevisiae) | | 9.406543989 | 3.98E-16 | cluster P | 1.42 |
| **PTRHD1** | peptidyl-tRNA hydrolase domain containing 1 | | 7.511973893 | 1.05E-11 | cluster P | 1.46 |
| **C5orf51** | chromosome 5 open reading frame 51 | | 10.43681668 | 1.19E-18 | cluster P | 1.44 |
| **COPG1** | coatomer protein complex, subunit gamma 1 | | 8.851685252 | 1.10E-14 | cluster P | 1.77 |
| **SREK1** | splicing regulatory glutamine/lysine-rich protein 1 | | 7.403517558 | 1.95E-11 | cluster P | 1.3 |
| **GID8** | GID complex subunit 8 homolog (S. cerevisiae) | | 15.23369431 | 1.35E-29 | cluster P | 3.05 |
| **BROX** | BRO1 domain and CAAX motif containing | | 8.542164755 | 4.89E-14 | cluster P | 1.52 |
| **IST1** | increased sodium tolerance 1 homolog (yeast) | | 12.69966466 | 1.90E-23 | cluster P | 1.91 |
| **GLTSCR1L** | GLTSCR1-like | | 9.888248863 | 2.87E-16 | cluster P | 1.61 |
| **PPP1R21** | protein phosphatase 1, regulatory subunit 21 | | 10.69592904 | 5.83E-19 | cluster P | 1.54 |
| **TAB2** | TGF-beta activated kinase 1/MAP3K7 binding protein 2 | | 3.928646452 | 0.000159021 | cluster P | 1.19 |
| **ACTG1** | actin, gamma 1 | | -8.851596125 | 6.23E-14 | cluster I | 1.26 |
| **ACTN2** | actinin, alpha 2 | | -5.19860909 | 1.52E-06 | cluster I | 1.14 |
| **ADCY6** | adenylate cyclase 6 | | -8.745079169 | 1.51E-13 | cluster I | 1.5 |
| **AKT2** | v-akt murine thymoma viral oncogene homolog 2 | | -7.186264705 | 4.13E-10 | cluster I | 1.72 |
| **ATP7B** | ATPase, Cu++ transporting, beta polypeptide | | -4.540462621 | 1.61E-05 | cluster I | 1.52 |
| **AUP1** | ancient ubiquitous protein 1 | | -6.37533062 | 1.15E-08 | cluster I | 1.24 |
| **BAD** | BCL2-associated agonist of cell death | | -4.079486039 | 0.000116325 | cluster I | 1.43 |
| **BCR** | breakpoint cluster region | | -4.498025038 | 2.19E-05 | cluster I | 1.34 |
| **FOXL2** | forkhead box L2 | | -4.279585942 | 6.39E-05 | cluster I | 1.24 |
| **C1QC** | complement component 1, q subcomponent, C chain | | -6.082159984 | 3.47E-08 | cluster I | 1.62 |
| **CAMK2A** | calcium/calmodulin-dependent protein kinase II alpha | | -3.675950523 | 0.000465594 | cluster I | 1.47 |
| **CDX2** | caudal type homeobox 2 | | -5.163119377 | 9.48E-07 | cluster I | 1.48 |
| **CHKB** | choline kinase beta | | -6.582873892 | 2.24E-09 | cluster I | 1.35 |
| **CLU** | clusterin | | -3.928173446 | 0.000201317 | cluster I | 1.31 |
| **LTB4R** | leukotriene B4 receptor | | -5.321035787 | 1.03E-06 | cluster I | 1.62 |
| **COL4A3** | collagen, type IV, alpha 3 (Goodpasture antigen) | | -6.527989695 | 6.86E-09 | cluster I | 1.93 |
| **CLDN4** | claudin 4 | | -3.800726276 | 0.000264373 | cluster I | 1.13 |
| **CREB1** | cAMP responsive element binding protein 1 | | -5.147977966 | 3.02E-06 | cluster I | 1.45 |
| **DGKG** | diacylglycerol kinase, gamma 90kDa | | -3.563121681 | 0.000656837 | cluster I | 1.31 |
| **DBC1** | deleted in bladder cancer 1 | | -3.700898985 | 0.000322832 | cluster I | 1.32 |
| **DBN1** | drebrin 1 | | -10.55060336 | 1.81E-17 | cluster I | 1.45 |
| **DCX** | doublecortin | | -4.256888366 | 5.63E-05 | cluster I | 1.45 |
| **DLX1** | distal-less homeobox 1 | | -4.477159576 | 2.49E-05 | cluster I | 1.35 |
| **DMD** | dystrophin | | -3.775450791 | 0.000302302 | cluster I | 1.16 |
| **DNAH9** | dynein, axonemal, heavy chain 9 | | -4.68820352 | 1.45E-05 | cluster I | 1.34 |
| **DPP6** | dipeptidyl-peptidase 6 | | -5.18672112 | 2.30E-06 | cluster I | 1.71 |
| **DTNA** | dystrobrevin, alpha | | -5.115763238 | 1.37E-06 | cluster I | 1.57 |
| **ECE1** | endothelin converting enzyme 1 | | -7.468008195 | 3.41E-11 | cluster I | 1.58 |
| **EFNA5** | ephrin-A5 | | -4.724103819 | 7.34E-06 | cluster I | 1.56 |
| **EPB41L1** | erythrocyte membrane protein band 4.1-like 1 | | -5.933084874 | 7.54E-08 | cluster I | 1.32 |
| **EPHB2** | EPH receptor B2 | | -3.589617124 | 0.000565746 | cluster I | 1.24 |
| **F10** | coagulation factor X | | -5.564650366 | 2.29E-07 | cluster I | 1.5 |
| **FSHR** | follicle stimulating hormone receptor | | -4.992401164 | 4.15E-06 | cluster I | 1.35 |
| **GAST** | gastrin | | -4.541001441 | 2.32E-05 | cluster I | 1.43 |
| **GBX2** | gastrulation brain homeobox 2 | | -6.295762143 | 4.15E-08 | cluster I | 2.43 |
| **GCK** | glucokinase (hexokinase 4) | | -4.138536203 | 0.000106029 | cluster I | 1.43 |
| **NR6A1** | nuclear receptor subfamily 6, group A, member 1 | | -3.902804484 | 0.000196465 | cluster I | 1.29 |
| **FFAR2** | free fatty acid receptor 2 | | -3.977718296 | 0.00020469 | cluster I | 2.7 |
| **GPX3** | glutathione peroxidase 3 (plasma) | | -4.413208542 | 2.69E-05 | cluster I | 1.15 |
| **GRID1** | glutamate receptor, ionotropic, delta 1 | | -4.963289703 | 4.94E-06 | cluster I | 1.6 |
| **GRM6** | glutamate receptor, metabotropic 6 | | -9.032450408 | 3.21E-14 | cluster I | 1.44 |
| **GSR** | glutathione reductase | | -5.472417636 | 7.11E-07 | cluster I | 1.68 |
| **HMGA1** | high mobility group AT-hook 1 | | -4.165374073 | 7.54E-05 | cluster I | 1.13 |
| **HNF4A** | hepatocyte nuclear factor 4, alpha | | -5.824951409 | 9.25E-08 | cluster I | 1.43 |
| **HNRNPA1** | heterogeneous nuclear ribonucleoprotein A1 | | -6.403314284 | 7.86E-09 | cluster I | 1.23 |
| **HOXA4** | homeobox A4 | | -4.363492965 | 4.43E-05 | cluster I | 1.6 |
| **AGFG2** | ArfGAP with FG repeats 2 | | -6.991823303 | 1.23E-09 | cluster I | 1.59 |
| **HSPA9** | heat shock 70kDa protein 9 (mortalin) | | -5.086999541 | 2.68E-06 | cluster I | 1.29 |
| **HTR1B** | 5-hydroxytryptamine (serotonin) receptor 1B, G protein-coupled | | -3.722458906 | 0.000388553 | cluster I | 1.29 |
| **HTR4** | 5-hydroxytryptamine (serotonin) receptor 4, G protein-coupled | | -5.401967593 | 7.10E-07 | cluster I | 1.23 |
| **IL10** | interleukin 10 | | -12.60462344 | 3.34E-19 | cluster I | 2.54 |
| **INSL3** | insulin-like 3 (Leydig cell) | | -3.714077097 | 0.000411381 | cluster I | 1.43 |
| **ITGA3** | integrin, alpha 3 (antigen CD49C, alpha 3 subunit of VLA-3 receptor) | | -7.096439132 | 1.64E-10 | cluster I | 1.62 |
| **KCNQ2** | potassium voltage-gated channel, KQT-like subfamily, member 2 | | -3.935755224 | 0.000186338 | cluster I | 1.28 |
| **KRT9** | keratin 9 | | -4.149096304 | 9.41E-05 | cluster I | 1.73 |
| **KRT19** | keratin 19 | | -4.771477649 | 5.53E-06 | cluster I | 1.66 |
| **KRT85** | keratin 85 | | -4.294163298 | 6.43E-05 | cluster I | 1.92 |
| **LGALS9** | lectin, galactoside-binding, soluble, 9 | | -5.785061565 | 1.49E-07 | cluster I | 1.56 |
| **FADS3** | fatty acid desaturase 3 | | -6.165148228 | 5.68E-08 | cluster I | 1.68 |
| **LPP** | LIM domain containing preferred translocation partner in lipoma | | -4.417884971 | 2.76E-05 | cluster I | 1.18 |
| **SMAD1** | SMAD family member 1 | | -5.171231806 | 2.31E-06 | cluster I | 1.72 |
| **MLH1** | mutL homolog 1, colon cancer, nonpolyposis type 2 (E. coli) | | -4.397984546 | 3.06E-05 | cluster I | 1.28 |
| **MSH3** | mutS homolog 3 (E. coli) | | -4.931095547 | 4.22E-06 | cluster I | 1.23 |
| **NEUROD2** | neuronal differentiation 2 | | -5.134372251 | 3.62E-06 | cluster I | 2.23 |
| **NFE2L1** | nuclear factor (erythroid-derived 2)-like 1 | | -9.158593609 | 8.32E-15 | cluster I | 1.5 |
| **NFIC** | nuclear factor I/C (CCAAT-binding transcription factor) | | -3.93410363 | 0.000187147 | cluster I | 1.49 |
| **CNOT3** | CCR4-NOT transcription complex, subunit 3 | | -5.633241301 | 2.70E-07 | cluster I | 1.35 |
| **OTX2** | orthodenticle homeobox 2 | | -5.24132548 | 1.07E-06 | cluster I | 1.15 |
| **PAX2** | paired box 2 | | -5.390911521 | 7.63E-07 | cluster I | 1.31 |
| **PAX5** | paired box 5 | | -5.60478423 | 4.12E-07 | cluster I | 2.22 |
| **PCCA** | propionyl CoA carboxylase, alpha polypeptide | | -8.164500587 | 1.20E-11 | cluster I | 1.76 |
| **ATP8B1** | ATPase, aminophospholipid transporter, class I, type 8B, member 1 | | -6.169167988 | 4.71E-08 | cluster I | 1.84 |
| **PKLR** | pyruvate kinase, liver and RBC | | -3.437731279 | 0.000952916 | cluster I | 1.44 |
| **PKP1** | plakophilin 1 (ectodermal dysplasia/skin fragility syndrome) | | -5.948096821 | 1.16E-07 | cluster I | 1.69 |
| **POLH** | polymerase (DNA directed), eta | | -4.205382141 | 6.37E-05 | cluster I | 1.16 |
| **POLR2K** | polymerase (RNA) II (DNA directed) polypeptide K, 7.0kDa | | -4.030609409 | 0.000138282 | cluster I | 1.2 |
| **POU3F2** | POU class 3 homeobox 2 | | -4.903810671 | 5.53E-06 | cluster I | 1.37 |
| **POU3F4** | POU class 3 homeobox 4 | | -4.167879582 | 0.000102292 | cluster I | 1.57 |
| **POU4F1** | POU class 4 homeobox 1 | | -6.264836968 | 2.06E-08 | cluster I | 2.2 |
| **PPARA** | peroxisome proliferator-activated receptor alpha | | -6.474754462 | 1.61E-08 | cluster I | 1.81 |
| **PPAT** | phosphoribosyl pyrophosphate amidotransferase | | -5.149428396 | 1.75E-06 | cluster I | 1.35 |
| **PRKCG** | protein kinase C, gamma | | -3.921802338 | 0.000160187 | cluster I | 1.19 |
| **PRKCH** | protein kinase C, eta | | -4.826201514 | 8.09E-06 | cluster I | 1.57 |
| **PKN1** | protein kinase N1 | | -8.772174171 | 7.98E-13 | cluster I | 1.52 |
| **PSMB5** | proteasome (prosome, macropain) subunit, beta type, 5 | | -5.889340915 | 7.98E-08 | cluster I | 1.37 |
| **PTPRS** | protein tyrosine phosphatase, receptor type, S | | -4.334908026 | 4.24E-05 | cluster I | 1.18 |
| **RABGGTA** | Rab geranylgeranyltransferase, alpha subunit | | -6.397599948 | 1.83E-08 | cluster I | 1.57 |
| **RARG** | retinoic acid receptor, gamma | | -4.798837836 | 7.85E-06 | cluster I | 1.61 |
| **REV3L** | REV3-like, polymerase (DNA directed), zeta, catalytic subunit | | -4.391037083 | 3.40E-05 | cluster I | 1.38 |
| **RGR** | retinal G protein coupled receptor | | -5.416300077 | 9.01E-07 | cluster I | 1.79 |
| **ROCK1** | Rho-associated, coiled-coil containing protein kinase 1 | | -4.015552448 | 0.00011636 | cluster I | 1.21 |
| **RPL18A** | ribosomal protein L18a | | -5.861405618 | 4.57E-08 | cluster I | 1.12 |
| **RPS9** | ribosomal protein S9 | | -14.43191203 | 6.65E-26 | cluster I | 1.48 |
| **RPS10** | ribosomal protein S10 | | -13.21495599 | 3.60E-24 | cluster I | 1.48 |
| **RPS25** | ribosomal protein S25 | | -9.923296566 | 5.70E-17 | cluster I | 1.25 |
| **S100A13** | S100 calcium binding protein A13 | | -6.71966662 | 1.48E-09 | cluster I | 1.34 |
| **SCN1B** | sodium channel, voltage-gated, type I, beta subunit | | -5.224566437 | 1.71E-06 | cluster I | 1.53 |
| **SCN5A** | sodium channel, voltage-gated, type V, alpha subunit | | -3.909459805 | 0.000183955 | cluster I | 1.38 |
| **SLC2A4** | solute carrier family 2 (facilitated glucose transporter), member 4 | | -5.038310609 | 3.20E-06 | cluster I | 1.18 |
| **SMTN** | smoothelin | | -5.528882125 | 3.27E-07 | cluster I | 1.59 |
| **SLC6A11** | solute carrier family 6 (neurotransmitter transporter, GABA), member 11 | | -6.098305385 | 6.15E-08 | cluster I | 2.02 |
| **SLC9A2** | solute carrier family 9, subfamily A (NHE2, cation proton antiporter 2), member 2 | | -6.595573463 | 2.28E-09 | cluster I | 1.31 |
| **SMARCA1** | SWI/SNF related, matrix associated, actin dependent regulator of chromatin, subfamily a, member 1 | | -3.748874764 | 0.000335908 | cluster I | 1.3 |
| **SUMO2** | SMT3 suppressor of mif two 3 homolog 2 (S. cerevisiae) | | -7.451475184 | 3.75E-11 | cluster I | 1.2 |
| **SOX9** | SRY (sex determining region Y)-box 9 | | -3.695128495 | 0.000369541 | cluster I | 1.42 |
| **SRD5A1** | steroid-5-alpha-reductase, alpha polypeptide 1 (3-oxo-5 alpha-steroid delta 4-dehydrogenase alpha 1) | | -4.017329712 | 0.000162497 | cluster I | 1.84 |
| **SRP68** | signal recognition particle 68kDa | | -5.407034326 | 5.08E-07 | cluster I | 1.33 |
| **SSTR5** | somatostatin receptor 5 | | -3.743494248 | 0.000375269 | cluster I | 1.41 |
| **ST5** | suppression of tumorigenicity 5 | | -7.645370928 | 3.52E-11 | cluster I | 1.59 |
| **CDKL5** | cyclin-dependent kinase-like 5 | | -3.439405077 | 0.000878343 | cluster I | 1.19 |
| **STX1A** | syntaxin 1A (brain) | | -4.292728541 | 6.72E-05 | cluster I | 2.17 |
| **TAF11** | TAF11 RNA polymerase II, TATA box binding protein (TBP)-associated factor, 28kDa | | -5.592154279 | 4.51E-07 | cluster I | 1.61 |
| **TAL1** | T-cell acute lymphocytic leukemia 1 | | -3.793592902 | 0.000254002 | cluster I | 1.13 |
| **TFAP2A** | transcription factor AP-2 alpha (activating enhancer binding protein 2 alpha) | | -4.225494193 | 5.52E-05 | cluster I | 1.44 |
| **TGIF1** | TGFB-induced factor homeobox 1 | | -5.987336775 | 3.04E-08 | cluster I | 1.4 |
| **TNNI1** | troponin I type 1 (skeletal, slow) | | -4.219607063 | 6.82E-05 | cluster I | 1.45 |
| **TNXB** | tenascin XB | | -5.990929524 | 1.22E-07 | cluster I | 1.92 |
| **TOP3A** | topoisomerase (DNA) III alpha | | -4.549884601 | 1.46E-05 | cluster I | 1.19 |
| **TP53BP2** | tumor protein p53 binding protein, 2 | | -8.254084205 | 6.98E-12 | cluster I | 1.37 |
| **TPM3** | tropomyosin 3 | | -9.206716592 | 4.10E-15 | cluster I | 1.23 |
| **TPM4** | tropomyosin 4 | | -5.400427626 | 6.33E-07 | cluster I | 1.19 |
| **TSHR** | thyroid stimulating hormone receptor | | -4.043391857 | 0.000125352 | cluster I | 1.14 |
| **UBE2G1** | ubiquitin-conjugating enzyme E2G 1 | | -3.997012382 | 0.000121017 | cluster I | 1.12 |
| **USP4** | ubiquitin specific peptidase 4 (proto-oncogene) | | -3.468575791 | 0.00079451 | cluster I | 1.15 |
| **UPK3A** | uroplakin 3A | | -4.170438727 | 6.99E-05 | cluster I | 1.13 |
| **ZBTB16** | zinc finger and BTB domain containing 16 | | -4.512543839 | 1.80E-05 | cluster I | 1.67 |
| **TAF15** | TAF15 RNA polymerase II, TATA box binding protein (TBP)-associated factor, 68kDa | | -9.113175773 | 4.27E-15 | cluster I | 1.34 |
| **NR0B2** | nuclear receptor subfamily 0, group B, member 2 | | -6.010578657 | 3.88E-08 | cluster I | 1.6 |
| **PPFIA3** | protein tyrosine phosphatase, receptor type, f polypeptide (PTPRF), interacting protein (liprin), alpha 3 | | -3.871903965 | 0.000208486 | cluster I | 1.13 |
| **DENR** | density-regulated protein | | -8.440303618 | 3.10E-12 | cluster I | 1.6 |
| **SSNA1** | Sjogren syndrome nuclear autoantigen 1 | | -4.677475386 | 1.04E-05 | cluster I | 1.32 |
| **GALNT4** | UDP-N-acetyl-alpha-D-galactosamine:polypeptide N-acetylgalactosaminyltransferase 4 (GalNAc-T4) | | -4.274890501 | 4.75E-05 | cluster I | 1.4 |
| **EDF1** | endothelial differentiation-related factor 1 | | -5.000238834 | 3.04E-06 | cluster I | 1.17 |
| **ADAM15** | ADAM metallopeptidase domain 15 | | -7.897564753 | 1.05E-11 | cluster I | 2.27 |
| **APLN** | apelin | | -6.015191528 | 9.55E-08 | cluster I | 1.77 |
| **ATP6V0D1** | ATPase, H+ transporting, lysosomal 38kDa, V0 subunit d1 | | -4.322124649 | 4.83E-05 | cluster I | 1.49 |
| **NEURL** | neuralized homolog (Drosophila) | | -4.993356241 | 3.66E-06 | cluster I | 1.16 |
| **GTF3C4** | general transcription factor IIIC, polypeptide 4, 90kDa | | -6.948396008 | 1.42E-09 | cluster I | 1.54 |
| **SLC22A6** | solute carrier family 22 (organic anion transporter), member 6 | | -7.862286303 | 1.12E-10 | cluster I | 2.83 |
| **LONP1** | lon peptidase 1, mitochondrial | | -6.390126422 | 1.57E-08 | cluster I | 1.52 |
| **PPT2** | palmitoyl-protein thioesterase 2 | | -4.198114549 | 8.33E-05 | cluster I | 1.47 |
| **CIAO1** | cytosolic iron-sulfur protein assembly 1 | | -4.890329555 | 4.74E-06 | cluster I | 1.14 |
| **ROCK2** | Rho-associated, coiled-coil containing protein kinase 2 | | -13.06383767 | 2.64E-24 | cluster I | 1.23 |
| **CELSR1** | cadherin, EGF LAG seven-pass G-type receptor 1 | | -4.53555564 | 2.35E-05 | cluster I | 1.33 |
| **SART3** | squamous cell carcinoma antigen recognized by T cells 3 | | -3.694354739 | 0.000441501 | cluster I | 1.14 |
| **ACAP1** | ArfGAP with coiled-coil, ankyrin repeat and PH domains 1 | | -4.046625387 | 0.000113876 | cluster I | 1.56 |
| **RCE1** | RCE1 homolog, prenyl protein protease (S. cerevisiae) | | -7.531816348 | 8.27E-11 | cluster I | 1.65 |
| **SCAMP2** | secretory carrier membrane protein 2 | | -6.453912383 | 5.97E-09 | cluster I | 1.66 |
| **RNF41** | ring finger protein 41 | | -7.051250222 | 3.10E-10 | cluster I | 1.29 |
| **CDK2AP2** | cyclin-dependent kinase 2 associated protein 2 | | -4.901059055 | 5.11E-06 | cluster I | 1.32 |
| **TLR6** | toll-like receptor 6 | | -4.654959803 | 1.68E-05 | cluster I | 1.61 |
| **DLC1** | deleted in liver cancer 1 | | -6.978034585 | 2.44E-10 | cluster I | 1.26 |
| **GNB2L1** | guanine nucleotide binding protein (G protein), beta polypeptide 2-like 1 | -8.477885942 | | 8.53E-14 | cluster I | 1.32 |
| **TESK2** | testis-specific kinase 2 | | -5.390075288 | 6.89E-07 | cluster I | 1.34 |
| **SEMA6C** | sema domain, transmembrane domain (TM), and cytoplasmic domain, (semaphorin) 6C | | -6.229519875 | 1.91E-08 | cluster I | 1.44 |
| **HYOU1** | hypoxia up-regulated 1 | | -6.066965588 | 6.71E-08 | cluster I | 1.74 |
| **RABAC1** | Rab acceptor 1 (prenylated) | | -9.570545067 | 6.79E-16 | cluster I | 1.6 |
| **IGF2BP1** | insulin-like growth factor 2 mRNA binding protein 1 | | -6.034035375 | 4.10E-08 | cluster I | 1.22 |
| **SIX2** | SIX homeobox 2 | | -3.495446363 | 0.000785321 | cluster I | 1.12 |
| **CHL1** | cell adhesion molecule with homology to L1CAM (close homolog of L1) | | -4.187180203 | 7.81E-05 | cluster I | 1.13 |
| **SDCCAG3** | serologically defined colon cancer antigen 3 | | -5.0947094 | 2.94E-06 | cluster I | 1.35 |
| **ZNF275** | zinc finger protein 275 | | -7.258374706 | 1.06E-10 | cluster I | 1.46 |
| **ME3** | malic enzyme 3, NADP(+)-dependent, mitochondrial | | -4.646036744 | 1.44E-05 | cluster I | 1.65 |
| **SPINK5** | serine peptidase inhibitor, Kazal type 5 | | -4.305611952 | 5.64E-05 | cluster I | 1.85 |
| **CCDC85B** | coiled-coil domain containing 85B | | -5.547391759 | 2.91E-07 | cluster I | 1.29 |
| **WDR45** | WD repeat domain 45 | | -4.525495247 | 1.93E-05 | cluster I | 1.28 |
| **BAZ1A** | bromodomain adjacent to zinc finger domain, 1A | | -6.182480888 | 1.70E-08 | cluster I | 1.37 |
| **SEC23IP** | SEC23 interacting protein | | -4.389982569 | 3.76E-05 | cluster I | 1.38 |
| **FZD10** | frizzled family receptor 10 | | -4.440055131 | 3.15E-05 | cluster I | 1.13 |
| **AKAP10** | A kinase (PRKA) anchor protein 10 | | -7.748493057 | 2.16E-11 | cluster I | 1.65 |
| **POU6F2** | POU class 6 homeobox 2 | | -5.03729317 | 3.91E-06 | cluster I | 2.13 |
| **MGAT4B** | mannosyl (alpha-1,3-)-glycoprotein beta-1,4-N-acetylglucosaminyltransferase, isozyme B | | -8.348168982 | 4.99E-12 | cluster I | 1.93 |
| **GABARAP** | GABA(A) receptor-associated protein | | -4.452316673 | 3.13E-05 | cluster I | 1.19 |
| **CNKSR2** | connector enhancer of kinase suppressor of Ras 2 | | -4.109672108 | 0.00010038 | cluster I | 1.12 |
| **INPP5F** | inositol polyphosphate-5-phosphatase F | | -4.228362062 | 7.20E-05 | cluster I | 1.16 |
| **MON1B** | MON1 homolog B (yeast) | | -4.6330254 | 2.15E-05 | cluster I | 2.76 |
| **RUFY3** | RUN and FYVE domain containing 3 | | -5.040432688 | 2.82E-06 | cluster I | 1.48 |
| **TBC1D2B** | TBC1 domain family, member 2B | | -5.574609488 | 5.18E-07 | cluster I | 1.73 |
| **CAMTA2** | calmodulin binding transcription activator 2 | | -3.828293494 | 0.000269887 | cluster I | 1.49 |
| **CIC** | capicua transcriptional repressor | | -5.377993107 | 7.14E-07 | cluster I | 1.29 |
| **NCDN** | neurochondrin | | -4.29693754 | 3.77E-05 | cluster I | 1.17 |
| **GANAB** | glucosidase, alpha; neutral AB | | -3.712149614 | 0.000367489 | cluster I | 1.12 |
| **ARC** | activity-regulated cytoskeleton-associated protein | | -3.751666352 | 0.000353204 | cluster I | 1.33 |
| **CLASP1** | cytoplasmic linker associated protein 1 | | -5.51779703 | 2.69E-07 | cluster I | 1.34 |
| **VPS39** | vacuolar protein sorting 39 homolog (S. cerevisiae) | | -4.958579622 | 5.22E-06 | cluster I | 1.57 |
| **CDC42EP4** | CDC42 effector protein (Rho GTPase binding) 4 | | -4.561894952 | 1.38E-05 | cluster I | 1.34 |
| **PRPF6** | PRP6 pre-mRNA processing factor 6 homolog (S. cerevisiae) | | -4.796778308 | 7.25E-06 | cluster I | 1.22 |
| **TNFAIP8** | tumor necrosis factor, alpha-induced protein 8 | | -5.838550494 | 9.08E-08 | cluster I | 1.32 |
| **FAM19A5** | family with sequence similarity 19 (chemokine (C-C motif)-like), member A5 | | -6.873805014 | 1.09E-09 | cluster I | 1.83 |
| **DNAJB5** | DnaJ (Hsp40) homolog, subfamily B, member 5 | | -5.836771013 | 1.03E-07 | cluster I | 1.47 |
| **USP49** | ubiquitin specific peptidase 49 | | -7.236135856 | 7.09E-10 | cluster I | 1.81 |
| **GPSM1** | G-protein signaling modulator 1 | | -5.13279471 | 2.38E-06 | cluster I | 1.45 |
| **TRPC4AP** | transient receptor potential cation channel, subfamily C, member 4 associated protein | | -9.63695528 | 1.00E-15 | cluster I | 1.39 |
| **TIAM2** | T-cell lymphoma invasion and metastasis 2 | | -3.718047362 | 0.000376921 | cluster I | 1.47 |
| **FBXO24** | F-box protein 24 | | -5.536290567 | 5.45E-07 | cluster I | 1.66 |
| **LHX6** | LIM homeobox 6 | | -3.967442089 | 0.00016603 | cluster I | 1.29 |
| **SEZ6L2** | seizure related 6 homolog (mouse)-like 2 | | -3.879149214 | 0.000232393 | cluster I | 1.29 |
| **DAZAP1** | DAZ associated protein 1 | | -4.216830507 | 6.59E-05 | cluster I | 1.16 |
| **SNORD56** | small nucleolar RNA, C/D box 56 | | -7.336795082 | 3.82E-11 | cluster I | 1.42 |
| **KLF15** | Kruppel-like factor 15 | | -4.801547831 | 6.23E-06 | cluster I | 1.48 |
| **UHRF1** | ubiquitin-like with PHD and ring finger domains 1 | | -4.687999111 | 9.23E-06 | cluster I | 1.34 |
| **PARVB** | parvin, beta | | -5.974514485 | 7.50E-08 | cluster I | 1.55 |
| **ANAPC2** | anaphase promoting complex subunit 2 | | -4.988368438 | 3.64E-06 | cluster I | 1.38 |
| **TRHDE** | thyrotropin-releasing hormone degrading enzyme | | -4.537162203 | 2.48E-05 | cluster I | 2.32 |
| **CYHR1** | cysteine/histidine-rich 1 | | -8.465552736 | 2.54E-12 | cluster I | 2.39 |
| **NEUROG3** | neurogenin 3 | | -3.983860593 | 0.000169063 | cluster I | 1.39 |
| **ST8SIA3** | ST8 alpha-N-acetyl-neuraminide alpha-2,8-sialyltransferase 3 | | -4.791618106 | 6.92E-06 | cluster I | 1.3 |
| **TNNI3K** | TNNI3 interacting kinase | | -5.311901139 | 1.32E-06 | cluster I | 1.57 |
| **MECR** | mitochondrial trans-2-enoyl-CoA reductase | | -7.404149288 | 2.94E-10 | cluster I | 1.76 |
| **C3orf18** | chromosome 3 open reading frame 18 | | -8.30004982 | 4.70E-12 | cluster I | 1.8 |
| **DUSP13** | dual specificity phosphatase 13 | | -6.198092615 | 4.64E-08 | cluster I | 1.35 |
| **ZNF771** | zinc finger protein 771 | | -5.772060711 | 1.00E-07 | cluster I | 1.38 |
| **WNT16** | wingless-type MMTV integration site family, member 16 | | -5.149231548 | 1.51E-06 | cluster I | 1.36 |
| **PIAS4** | protein inhibitor of activated STAT, 4 | | -6.774209517 | 5.45E-10 | cluster I | 1.12 |
| **GPRC5B** | G protein-coupled receptor, family C, group 5, member B | | -4.427781506 | 2.95E-05 | cluster I | 1.16 |
| **TPCN1** | two pore segment channel 1 | | -5.306826348 | 5.72E-07 | cluster I | 1.35 |
| **MBD3** | methyl-CpG binding domain protein 3 | | -5.573385754 | 2.82E-07 | cluster I | 1.44 |
| **RIPK4** | receptor-interacting serine-threonine kinase 4 | | -6.09509738 | 4.69E-08 | cluster I | 1.23 |
| **MBTD1** | mbt domain containing 1 | | -10.65573966 | 2.30E-17 | cluster I | 1.35 |
| **APTX** | aprataxin | | -4.173943552 | 5.96E-05 | cluster I | 1.31 |
| **MKS1** | Meckel syndrome, type 1 | | -5.146032975 | 2.11E-06 | cluster I | 1.17 |
| **C14orf119** | chromosome 14 open reading frame 119 | | -6.15493023 | 5.05E-08 | cluster I | 1.34 |
| **SAMD4B** | sterile alpha motif domain containing 4B | | -4.645132828 | 1.12E-05 | cluster I | 1.29 |
| **GPATCH2** | G patch domain containing 2 | | -4.350037913 | 3.93E-05 | cluster I | 1.45 |
| **DALRD3** | DALR anticodon binding domain containing 3 | | -3.847938942 | 0.000310668 | cluster I | 1.53 |
| **ABCF3** | ATP-binding cassette, sub-family F (GCN20), member 3 | | -7.269154 | 3.92E-10 | cluster I | 1.82 |
| **SLC39A9** | solute carrier family 39 (zinc transporter), member 9 | | -5.240961405 | 1.19E-06 | cluster I | 1.32 |
| **C4orf21** | chromosome 4 open reading frame 21 | | -4.526511285 | 2.73E-05 | cluster I | 1.62 |
| **ZCCHC8** | zinc finger, CCHC domain containing 8 | | -4.55911521 | 2.07E-05 | cluster I | 1.35 |
| **LMBR1L** | limb region 1 homolog (mouse)-like | | -4.836565937 | 7.13E-06 | cluster I | 1.19 |
| **WDR12** | WD repeat domain 12 | | -5.571471672 | 4.13E-07 | cluster I | 1.63 |
| **FAM63A** | family with sequence similarity 63, member A | | -3.797998679 | 0.000303852 | cluster I | 1.15 |
| **LMO3** | LIM domain only 3 (rhombotin-like 2) | | -3.439561494 | 0.00097292 | cluster I | 1.19 |
| **ZMAT5** | zinc finger, matrin-type 5 | | -9.441511594 | 1.83E-14 | cluster I | 1.65 |
| **NDUFA12** | NADH dehydrogenase (ubiquinone) 1 alpha subcomplex, 12 | | -4.231890553 | 6.08E-05 | cluster I | 1.13 |
| **PCDHGB6** | protocadherin gamma subfamily B, 6 | | -4.48905699 | 2.35E-05 | cluster I | 1.55 |
| **KCNK12** | potassium channel, subfamily K, member 12 | | -4.761585562 | 6.16E-06 | cluster I | 1.43 |
| **PCBP4** | poly(rC) binding protein 4 | | -3.937061091 | 0.000160708 | cluster I | 1.28 |
| **ERGIC1** | endoplasmic reticulum-golgi intermediate compartment (ERGIC) 1 | | -3.932266549 | 0.000176193 | cluster I | 1.19 |
| **GJD2** | gap junction protein, delta 2, 36kDa | | -5.047242023 | 2.16E-06 | cluster I | 1.24 |
| **KIAA1161** | KIAA1161 | | -4.334098253 | 4.98E-05 | cluster I | 1.23 |
| **HHATL** | hedgehog acyltransferase-like | | -5.253742406 | 1.13E-06 | cluster I | 1.23 |
| **USP31** | ubiquitin specific peptidase 31 | | -6.135366181 | 4.31E-08 | cluster I | 1.57 |
| **XPO5** | exportin 5 | | -5.849260223 | 8.33E-08 | cluster I | 1.3 |
| **CRAMP1L** | Crm, cramped-like (Drosophila) | | -5.559162345 | 4.92E-07 | cluster I | 1.96 |
| **CACNG7** | calcium channel, voltage-dependent, gamma subunit 7 | | -4.426433294 | 3.78E-05 | cluster I | 1.57 |
| **BACH2** | BTB and CNC homology 1, basic leucine zipper transcription factor 2 | | -4.140198853 | 0.000102338 | cluster I | 1.81 |
| **PCIF1** | PDX1 C-terminal inhibiting factor 1 | | -6.005592193 | 8.27E-08 | cluster I | 1.72 |
| **NEUROG2** | neurogenin 2 | | -4.708590302 | 1.39E-05 | cluster I | 2.21 |
| **PRDM14** | PR domain containing 14 | | -3.710063899 | 0.000409637 | cluster I | 1.34 |
| **CDH24** | cadherin 24, type 2 | | -5.108746762 | 1.49E-06 | cluster I | 1.12 |
| **HS3ST6** | heparan sulfate (glucosamine) 3-O-sulfotransferase 6 | | -4.478484261 | 3.70E-05 | cluster I | 2.37 |
| **EPS8L2** | EPS8-like 2 | | -8.338540414 | 6.67E-12 | cluster I | 2.02 |
| **LMF1** | lipase maturation factor 1 | | -4.247960803 | 6.48E-05 | cluster I | 1.6 |
| **ELOVL1** | ELOVL fatty acid elongase 1 | | -7.468909416 | 4.62E-11 | cluster I | 1.46 |
| **PORCN** | porcupine homolog (Drosophila) | | -4.570300941 | 1.80E-05 | cluster I | 1.31 |
| **MRPL34** | mitochondrial ribosomal protein L34 | | -9.890528832 | 3.91E-16 | cluster I | 1.57 |
| **VPS33A** | vacuolar protein sorting 33 homolog A (S. cerevisiae) | | -4.516171998 | 2.60E-05 | cluster I | 1.17 |
| **ZSWIM4** | zinc finger, SWIM-type containing 4 | | -9.204988461 | 3.93E-15 | cluster I | 1.23 |
| **MMP28** | matrix metallopeptidase 28 | | -4.395281575 | 2.41E-05 | cluster I | 1.47 |
| **TNIP2** | TNFAIP3 interacting protein 2 | | -6.032199748 | 4.40E-08 | cluster I | 1.38 |
| **RBM42** | RNA binding motif protein 42 | | -7.670846864 | 4.30E-11 | cluster I | 1.26 |
| **IRX1** | iroquois homeobox 1 | | -4.029901742 | 0.000123964 | cluster I | 1.15 |
| **TTLL7** | tubulin tyrosine ligase-like family, member 7 | | -4.302171484 | 6.21E-05 | cluster I | 1.9 |
| **GSDMD** | gasdermin D | | -5.46513842 | 4.49E-07 | cluster I | 1.17 |
| **ARMC5** | armadillo repeat containing 5 | | -3.980566614 | 0.000134461 | cluster I | 1.2 |
| **SNX22** | sorting nexin 22 | | -7.778653658 | 1.00E-11 | cluster I | 1.42 |
| **TRPM3** | transient receptor potential cation channel, subfamily M, member 3 | | -4.005804098 | 0.000131048 | cluster I | 1.54 |
| **KCNH6** | potassium voltage-gated channel, subfamily H (eag-related), member 6 | | -6.09171303 | 2.95E-08 | cluster I | 1.25 |
| **WBSCR16** | Williams-Beuren syndrome chromosome region 16 | | -4.948921666 | 3.83E-06 | cluster I | 1.33 |
| **TSSK3** | testis-specific serine kinase 3 | | -5.525206575 | 3.63E-07 | cluster I | 1.29 |
| **TIGD6** | tigger transposable element derived 6 | | -5.31446787 | 8.34E-07 | cluster I | 1.25 |
| **TFAP2D** | transcription factor AP-2 delta (activating enhancer binding protein 2 delta) | | -4.470437409 | 3.58E-05 | cluster I | 2.06 |
| **PHF6** | PHD finger protein 6 | | -3.788775737 | 0.000274286 | cluster I | 1.14 |
| **BRSK1** | BR serine/threonine kinase 1 | | -3.90904314 | 0.00015919 | cluster I | 1.26 |
| **DCTN5** | dynactin 5 (p25) | | -7.771995614 | 1.08E-11 | cluster I | 1.45 |
| **C21orf67** | chromosome 21 open reading frame 67 | | -4.418104607 | 3.45E-05 | cluster I | 1.16 |
| **PARD6G** | par-6 partitioning defective 6 homolog gamma (C. elegans) | | -8.143153322 | 3.90E-12 | cluster I | 2.06 |
| **PHF5A** | PHD finger protein 5A | | -8.901663752 | 2.81E-14 | cluster I | 1.36 |
| **PARP10** | poly (ADP-ribose) polymerase family, member 10 | | -9.467563859 | 1.71E-14 | cluster I | 1.39 |
| **ZNF341** | zinc finger protein 341 | | -4.883656089 | 7.31E-06 | cluster I | 1.69 |
| **ZNRF1** | zinc and ring finger 1, E3 ubiquitin protein ligase | | -4.18190976 | 7.34E-05 | cluster I | 1.14 |
| **AGXT2L2** | alanine-glyoxylate aminotransferase 2-like 2 | | -8.849813077 | 9.98E-13 | cluster I | 1.66 |
| **SHANK3** | SH3 and multiple ankyrin repeat domains 3 | | -5.185503706 | 1.91E-06 | cluster I | 1.45 |
| **COX19** | cytochrome c oxidase assembly homolog 19 (S. cerevisiae) | | -3.802686236 | 0.000240834 | cluster I | 1.19 |
| **PYGO2** | pygopus homolog 2 (Drosophila) | | -9.634878853 | 2.19E-14 | cluster I | 2.03 |
| **ZC3HAV1L** | zinc finger CCCH-type, antiviral 1-like | | -11.46521343 | 1.01E-16 | cluster I | 2.23 |
| **MARS2** | methionyl-tRNA synthetase 2, mitochondrial | | -5.468334375 | 6.86E-07 | cluster I | 1.17 |
| **NT5C1B** | 5'-nucleotidase, cytosolic IB | | -6.107747717 | 4.75E-08 | cluster I | 1.79 |
| **PALM2** | paralemmin 2 | | -3.600997003 | 0.000591419 | cluster I | 1.18 |
| **BTBD9** | BTB (POZ) domain containing 9 | | -4.008691338 | 0.000135172 | cluster I | 1.13 |
| **DDIT4L** | DNA-damage-inducible transcript 4-like | | -4.334874624 | 5.50E-05 | cluster I | 2.15 |
| **CYP2R1** | cytochrome P450, family 2, subfamily R, polypeptide 1 | | -7.942733966 | 2.91E-11 | cluster I | 1.94 |
| **JDP2** | Jun dimerization protein 2 | | -4.061906698 | 0.000102471 | cluster I | 1.16 |
| **SLC25A29** | solute carrier family 25 (mitochondrial carnitine/acylcarnitine carrier), member 29 | | -4.323090096 | 4.94E-05 | cluster I | 1.34 |
| **CANT1** | calcium activated nucleotidase 1 | | -3.77160267 | 0.000291189 | cluster I | 1.28 |
| **EID2B** | EP300 interacting inhibitor of differentiation 2B | | -10.82018908 | 9.98E-16 | cluster I | 1.89 |
| **UBXN10** | UBX domain protein 10 | | -4.900233535 | 6.27E-06 | cluster I | 1.16 |
| **ARL8A** | ADP-ribosylation factor-like 8A | | -4.073936961 | 0.000101253 | cluster I | 1.17 |
| **IQGAP3** | IQ motif containing GTPase activating protein 3 | | -6.8980807 | 8.50E-10 | cluster I | 1.2 |
| **ANKRD54** | ankyrin repeat domain 54 | | -7.536030965 | 1.85E-11 | cluster I | 1.44 |
| **TRIM71** | tripartite motif containing 71, E3 ubiquitin protein ligase | | -3.522086513 | 0.000658591 | cluster I | 1.26 |
| **LSM11** | LSM11, U7 small nuclear RNA associated | | -5.841276417 | 4.79E-08 | cluster I | 1.28 |
| **OR2A14** | olfactory receptor, family 2, subfamily A, member 14 | | -3.696709477 | 0.00049864 | cluster I | 1.57 |
| **C9orf41** | chromosome 9 open reading frame 41 | | -6.957385191 | 2.80E-09 | cluster I | 2.91 |
| **ACTRT1** | actin-related protein T1 | | -6.96094294 | 1.64E-09 | cluster I | 1.46 |
| **SLC32A1** | solute carrier family 32 (GABA vesicular transporter), member 1 | | -4.927536841 | 5.84E-06 | cluster I | 1.85 |
| **DYNLL2** | dynein, light chain, LC8-type 2 | | -9.347712635 | 3.25E-14 | cluster I | 1.63 |
| **DUSP19** | dual specificity phosphatase 19 | | -7.172186911 | 4.87E-10 | cluster I | 1.52 |
| **LDLRAD3** | low density lipoprotein receptor class A domain containing 3 | | -4.93067014 | 3.82E-06 | cluster I | 1.44 |
| **PRIMA1** | proline rich membrane anchor 1 | | -3.763720761 | 0.000335721 | cluster I | 1.57 |
| **NOTUM** | notum pectinacetylesterase homolog (Drosophila) | | -3.678359502 | 0.00045999 | cluster I | 1.42 |
| **WIPF2** | WAS/WASL interacting protein family, member 2 | | -5.815924045 | 1.41E-07 | cluster I | 1.22 |
| **CCBE1** | collagen and calcium binding EGF domains 1 | | -14.20927366 | 6.23E-23 | cluster I | 1.69 |
| **PPM1K** | protein phosphatase, Mg2+/Mn2+ dependent, 1K | | -9.324145273 | 6.71E-14 | cluster I | 1.41 |
| **C9orf163** | chromosome 9 open reading frame 163 | | -4.355780081 | 4.78E-05 | cluster I | 2.39 |
| **TTC16** | tetratricopeptide repeat domain 16 | | -4.273501915 | 4.93E-05 | cluster I | 1.19 |
| **SLC16A11** | solute carrier family 16, member 11 (monocarboxylic acid transporter 11) | | -5.089572088 | 2.30E-06 | cluster I | 1.74 |
| **C1orf65** | chromosome 1 open reading frame 65 | | -4.768385871 | 1.04E-05 | cluster I | 1.73 |
| **CABP7** | calcium binding protein 7 | | -7.420002168 | 1.78E-10 | cluster I | 1.73 |
| **LIX1** | Lix1 homolog (chicken) | | -4.957670362 | 5.44E-06 | cluster I | 1.92 |
| **PWWP2B** | PWWP domain containing 2B | | -4.924456066 | 4.61E-06 | cluster I | 1.31 |
| **DAND5** | DAN domain family member 5, BMP antagonist | | -5.415549207 | 4.42E-07 | cluster I | 1.5 |
| **SLC16A13** | solute carrier family 16, member 13 (monocarboxylic acid transporter 13) | | -6.972491519 | 1.50E-09 | cluster I | 1.68 |
| **OR8H1** | olfactory receptor, family 8, subfamily H, member 1 | | -3.770518065 | 0.000328824 | cluster I | 1.18 |
| **OR5B21** | olfactory receptor, family 5, subfamily B, member 21 | | -3.836580283 | 0.000305256 | cluster I | 1.58 |
| **OR4D6** | olfactory receptor, family 4, subfamily D, member 6 | | -7.46358292 | 2.24E-10 | cluster I | 2.57 |
| **RNF152** | ring finger protein 152 | | -4.642780737 | 1.36E-05 | cluster I | 1.43 |
| **HNRNPA3** | heterogeneous nuclear ribonucleoprotein A3 | | -6.41917912 | 9.30E-09 | cluster I | 1.35 |
| **LRRN4CL** | LRRN4 C-terminal like | | -6.238457152 | 3.93E-08 | cluster I | 1.91 |
| **HIST1H2AA** | histone cluster 1, H2aa | | -3.994349849 | 0.000181302 | cluster I | 2.17 |
| **C11orf35** | chromosome 11 open reading frame 35 | | -4.60688793 | 1.79E-05 | cluster I | 1.52 |
| **MAGI3** | membrane associated guanylate kinase, WW and PDZ domain containing 3 | | -8.88045982 | 4.28E-13 | cluster I | 2.55 |
| **SNHG10** | small nucleolar RNA host gene 10 (non-protein coding) | | -10.5130148 | 3.09E-16 | cluster I | 1.34 |
| **C15orf37** | chromosome 15 open reading frame 37 | | -10.32003671 | 4.06E-15 | cluster I | 1.79 |
| **DTWD2** | DTW domain containing 2 | | -8.377744693 | 1.36E-12 | cluster I | 1.26 |
| **FAM83H** | family with sequence similarity 83, member H | | -7.637870031 | 6.30E-11 | cluster I | 1.66 |
| **RSPO2** | R-spondin 2 | | -5.760706039 | 2.31E-07 | cluster I | 1.24 |
| **ZCCHC16** | zinc finger, CCHC domain containing 16 | | -4.307541879 | 6.04E-05 | cluster I | 1.96 |
| **VWA2** | von Willebrand factor A domain containing 2 | | -3.81980984 | 0.000306786 | cluster I | 1.84 |
| **OR8S1** | olfactory receptor, family 8, subfamily S, member 1 | | -3.601535191 | 0.000598527 | cluster I | 1.54 |
| **TCTEX1D4** | Tctex1 domain containing 4 | | -4.504886663 | 2.40E-05 | cluster I | 1.3 |
| **HSD11B1L** | hydroxysteroid (11-beta) dehydrogenase 1-like | | -5.007336538 | 3.55E-06 | cluster I | 1.38 |
| **C1orf95** | chromosome 1 open reading frame 95 | | -8.112380574 | 7.55E-12 | cluster I | 1.87 |
| **FAM89A** | family with sequence similarity 89, member A | | -3.576486188 | 0.000605799 | cluster I | 1.18 |
| **TMEM110** | transmembrane protein 110 | | -7.74434148 | 2.87E-11 | cluster I | 1.87 |
| **FOXI2** | forkhead box I2 | | -5.108011378 | 2.70E-06 | cluster I | 1.61 |
| **CRIP3** | cysteine-rich protein 3 | | -7.031858067 | 2.32E-10 | cluster I | 1.44 |
| **PTPLAD2** | protein tyrosine phosphatase-like A domain containing 2 | | -12.21938363 | 2.95E-20 | cluster I | 2.06 |
| **HERC2P4** | hect domain and RLD 2 pseudogene 4 | | -3.964931016 | 0.00017484 | cluster I | 1.35 |
| **NRARP** | NOTCH-regulated ankyrin repeat protein | | -9.05146146 | 3.04E-14 | cluster I | 2.58 |
| **LRRC37A2** | leucine rich repeat containing 37, member A2 | | -6.103671498 | 5.54E-08 | cluster I | 2.04 |
| **RNF165** | ring finger protein 165 | | -4.861966477 | 7.03E-06 | cluster I | 1.93 |
| **LOC595101** | smg-1 homolog, phosphatidylinositol 3-kinase-related kinase (C. elegans) pseudogene | | -4.140165893 | 9.37E-05 | cluster I | 1.42 |
| **SERINC4** | serine incorporator 4 | | -4.298999718 | 4.80E-05 | cluster I | 1.25 |
| **RASSF10** | Ras association (RalGDS/AF-6) domain family (N-terminal) member 10 | | -3.684069691 | 0.000451452 | cluster I | 1.85 |
| **GALNT18** | UDP-N-acetyl-alpha-D-galactosamine:polypeptide N-acetylgalactosaminyltransferase 18 | | -3.80098656 | 0.000306599 | cluster I | 1.52 |
| **RBFOX1** | RNA binding protein, fox-1 homolog (C. elegans) 1 | | -3.522023916 | 0.000732158 | cluster I | 1.38 |
| **TMEM259** | transmembrane protein 259 | | -7.658780925 | 2.94E-11 | cluster I | 1.42 |
| **ASIC2** | acid-sensing (proton-gated) ion channel 2 | | -5.706384739 | 1.91E-07 | cluster I | 1.72 |
| **AMER2** | APC membrane recruitment protein 2 | | -5.171195044 | 2.20E-06 | cluster I | 1.35 |
| **GREB1L** | growth regulation by estrogen in breast cancer-like | | -5.163126044 | 2.01E-06 | cluster I | 1.84 |
| **IGDCC3** | immunoglobulin superfamily, DCC subclass, member 3 | | -4.168363135 | 7.25E-05 | cluster I | 1.71 |
| **PABPC1L2B** | poly(A) binding protein, cytoplasmic 1-like 2B | | -4.600105217 | 2.46E-05 | cluster I | 1.35 |
| **ASIC4** | acid-sensing (proton-gated) ion channel family member 4 | | -3.858999376 | 0.000241354 | cluster I | 1.17 |
| **C17orf104** | chromosome 17 open reading frame 104 | | -9.17948022 | 3.71E-13 | cluster I | 1.59 |
| **C7orf55** | chromosome 7 open reading frame 55 | | -9.071170533 | 3.96E-13 | cluster I | 1.54 |
| **FAM189B** | family with sequence similarity 189, member B | | -4.312884922 | 3.31E-05 | cluster I | 1.23 |
| **HAUS2** | HAUS augmin-like complex, subunit 2 | | -12.38824403 | 2.32E-20 | cluster I | 1.84 |
| **TRMT2A** | tRNA methyltransferase 2 homolog A (S. cerevisiae) | | -3.734161609 | 0.00031424 | cluster I | 1.31 |
| **MGARP** | mitochondria-localized glutamic acid-rich protein | | -4.4559462 | 3.09E-05 | cluster I | 1.6 |
| **LINC00303** | long intergenic non-protein coding RNA 303 | | -3.621744005 | 0.000535461 | cluster I | 1.27 |
| **NTPCR** | nucleoside-triphosphatase, cancer-related | | -5.983572729 | 5.77E-08 | cluster I | 1.39 |
| **ZBTB14** | zinc finger and BTB domain containing 14 | | -5.121072957 | 2.36E-06 | cluster I | 1.43 |
| **KDM2B** | lysine (K)-specific demethylase 2B | | -4.023827585 | 0.000112259 | cluster I | 1.13 |
| **NOP2** | NOP2 nucleolar protein | | -4.189914631 | 7.53E-05 | cluster I | 1.17 |
| **U2SURP** | U2 snRNP-associated SURP domain containing | | -4.544881225 | 1.85E-05 | cluster I | 1.28 |
| **CAMSAP3** | calmodulin regulated spectrin-associated protein family, member 3 | | -6.523644296 | 4.45E-09 | cluster I | 1.54 |
| **CDHR5** | cadherin-related family member 5 | | -5.296095499 | 1.38E-06 | cluster I | 1.71 |
| **BEND3** | BEN domain containing 3 | | -9.687760145 | 1.50E-15 | cluster I | 2.1 |
| **TMEM204** | transmembrane protein 204 | | -3.599253717 | 0.00054893 | cluster I | 1.61 |
| **KIAA1211L** | KIAA1211-like | | -5.10514176 | 3.36E-06 | cluster I | 2.28 |
| **LURAP1** | leucine rich adaptor protein 1 | | -3.858939532 | 0.000245881 | cluster I | 1.47 |
| **PTCHD4** | patched domain containing 4 | | -4.829536012 | 6.19E-06 | cluster I | 1.36 |
| **EPPIN** | epididymal peptidase inhibitor | | -4.902060161 | 6.45E-06 | cluster I | 1.21 |
| **FAM213A** | family with sequence similarity 213, member A | | -12.41389561 | 6.14E-23 | cluster I | 1.35 |
| **STRIP2** | striatin interacting protein 2 | | -5.412934222 | 6.54E-07 | cluster I | 1.33 |
| **PLEC** | plectin | | -4.619388912 | 1.08E-05 | cluster I | 1.26 |
| **RUSC1-AS1** | RUSC1 antisense RNA 1 | | -4.904660212 | 4.80E-06 | cluster I | 1.15 |
| **XIRP2** | xin actin-binding repeat containing 2 | | -4.079633381 | 0.000126952 | cluster I | 1.46 |
| **LIN28A** | lin-28 homolog A (C. elegans) | | -4.735322201 | 1.09E-05 | cluster I | 1.93 |
| **PIANP** | PILR alpha associated neural protein | | -4.478714182 | 3.60E-05 | cluster I | 1.87 |
| **FITM2** | fat storage-inducing transmembrane protein 2 | | -3.930527181 | 0.000219267 | cluster I | 1.95 |
| **AAR2** | AAR2 splicing factor homolog (S. cerevisiae) | | -5.237490334 | 1.54E-06 | cluster I | 1.68 |
| **GATSL3** | GATS protein-like 3 | | -5.908003099 | 1.15E-07 | cluster I | 1.25 |
| **RSPH9** | radial spoke head 9 homolog (Chlamydomonas) | | -5.119866085 | 3.55E-06 | cluster I | 1.7 |
| **KLHL38** | kelch-like family member 38 | | -5.044090293 | 4.21E-06 | cluster I | 1.65 |
| **ARHGAP36** | Rho GTPase activating protein 36 | | -4.56184664 | 2.26E-05 | cluster I | 1.49 |

**Differentially expressed genes from CNV-ICC-TRN between cluster I and cluster P.**

Differential expression analysis was tested using t-test, and significance threshold was p-value<0.001.
