# Supplementary material for: Integrative Analysis of Transcriptional Regulatory Network and Copy Number Variation in Intrahepatic Cholangiocarcinoma
Source: PLoS One. 2014 Jun 4;9(6):e98653. doi: 10.1371/journal.pone.0098653 (PMC4045758; doi:10.1371/journal.pone.0098653)
Supplement: Table S7 — KEGG pathways enriched by each class's relatively high-expressed genes. Enrichment analysis was performed using one-side Fisher's exact test, and significance threshold was p-value<0.05. (DOC) [file pone.0098653.s008.doc]

**SI-Table 7.**

| **Class** | **Annotation** | **Genes in Gene Set (K)** | **Genes in Overlap (k)** | **p-value** |
| --- | --- | --- | --- | --- |
| **cluster I** | Arrhythmogenic right ventricular cardiomyopathy (ARVC)~5412 | 74 | 5 | 0.026 |
| **cluster I** | Focal adhesion~4510 | 200 | 10 | 0.015 |
| **cluster I** | Tight junction~4530 | 133 | 9 | 0.003 |
| **cluster I** | Leukocyte transendothelial migration~4670 | 117 | 6 | 0.047 |
| **cluster I** | Hypertrophic cardiomyopathy (HCM)~5410 | 83 | 6 | 0.012 |
| **cluster I** | Dilated cardiomyopathy~5414 | 90 | 7 | 0.005 |
| **cluster I** | Melanogenesis~4916 | 101 | 6 | 0.027 |
| **cluster I** | Vasopressin-regulated water reabsorption~4962 | 44 | 4 | 0.019 |
| **cluster I** | Maturity onset diabetes of the young~4950 | 25 | 4 | 0.003 |
| **cluster P** | MAPK signaling pathway~4010 | 268 | 21 | 0.048 |
| **cluster P** | ErbB signaling pathway~4012 | 87 | 10 | 0.021 |
| **cluster P** | mTOR signaling pathway~4150 | 52 | 7 | 0.025 |
| **cluster P** | T cell receptor signaling pathway~4660 | 108 | 13 | 0.006 |
| **cluster P** | B cell receptor signaling pathway~4662 | 75 | 9 | 0.022 |
| **cluster P** | Neurotrophin signaling pathway~4722 | 127 | 16 | 0.002 |
| **cluster P** | Pathways in cancer~5200 | 327 | 25 | 0.042 |
| **cluster P** | Renal cell carcinoma~5211 | 70 | 8 | 0.037 |
| **cluster P** | RNA transport~3013 | 152 | 15 | 0.018 |
| **cluster P** | Ether lipid metabolism~0565 | 36 | 5 | 0.049 |
| **cluster P** | Wnt signaling pathway~4310 | 151 | 16 | 0.008 |
| **cluster P** | Protein processing in endoplasmic reticulum~4141 | 168 | 20 | 0.001 |
| **cluster P** | Nucleotide excision repair~3420 | 45 | 7 | 0.013 |
| **cluster P** | Ubiquitin mediated proteolysis~4120 | 139 | 20 | 0 |
| **cluster P** | Cell cycle~4110 | 128 | 16 | 0.002 |
| **cluster P** | Oocyte meiosis~4144 | 114 | 13 | 0.01 |
| **cluster P** | TGF-beta signaling pathway~4350 | 85 | 11 | 0.007 |
| **cluster P** | Circadian rhythm - mammal~4710 | 23 | 4 | 0.041 |
| **cluster P** | Pathogenic Escherichia coli infection~5130 | 58 | 8 | 0.015 |
| **cluster P** | Bacterial invasion of epithelial cells~5100 | 71 | 8 | 0.04 |
| **cluster P** | Oxidative phosphorylation~0190 | 132 | 13 | 0.026 |
| **cluster P** | Spliceosome~3040 | 128 | 19 | 0 |
| **cluster P** | Regulation of actin cytoskeleton~4810 | 214 | 18 | 0.037 |
| **cluster P** | Shigellosis~5131 | 62 | 8 | 0.021 |
| **cluster P** | Axon guidance~4360 | 130 | 14 | 0.011 |
| **cluster P** | Adherens junction~4520 | 73 | 8 | 0.045 |
| **cluster P** | mRNA surveillance pathway~3015 | 83 | 10 | 0.016 |
| **cluster P** | Alzheimer's disease~5010 | 168 | 16 | 0.019 |
| **cluster P** | Epithelial cell signaling in Helicobacter pylori infection~5120 | 68 | 8 | 0.033 |
| **cluster P** | Cysteine and methionine metabolism~270 | 36 | 5 | 0.049 |
| **cluster P** | Long-term potentiation~4720 | 70 | 8 | 0.037 |
| **cluster P** | Vasopressin-regulated water reabsorption~4962 | 44 | 6 | 0.035 |
| **cluster P** | DNA replication~3030 | 36 | 5 | 0.049 |
| **cluster P** | Protein export~3060 | 23 | 4 | 0.041 |

**KEGG pathways enriched by each class’s relatively high-expressed genes.**

Enrichment analysis was performed using one-side Fisher’s exact test, and significance threshold was p-value<0.05**.**
