# Supplementary material for: Integrative Analysis of Transcriptional Regulatory Network and Copy Number Variation in Intrahepatic Cholangiocarcinoma
Source: PLoS One. 2014 Jun 4;9(6):e98653. doi: 10.1371/journal.pone.0098653 (PMC4045758; doi:10.1371/journal.pone.0098653)
Supplement: File S1 — Addition to the method section. (DOC) [file pone.0098653.s009.doc]

## The forward-and-reverse combined engineering

The forward engineering infers regulatory relationships between TFs and their putative targets based the matching or complementary of motif or seed sequences. In our work, we used sequence based TF-gene pairs from cGRNB (<http://www.scbit.org/cgrnb/>) which predicts TF-gene regulatory relationships according to whether TFs’ binding sites are in the range of 1kb upstream to 0.5kb downstream of genes’ transcription start sites.[[[1]](#endnote-2)]

The reverse engineering reveals the structure of the gene regulatory network in a biological system by reasoning backward directly from expression data.[[[2]](#endnote-3)] Several methods have been used reversed engineering prediction, like linear regression model, Bayesian networks and dynamic Bayesian networks, methods based on mutual information, and so on. Pairs in network predicted by this method are expression correlated.

In our work, we used forward-and-reverse combined method to predict transcriptional regulatory network, pairs of which are not only sequence correlated but also expression correlated.

## The Context Likelihood of Relatedness network inference algorithm

The CLR algorithm is an extension of the relevance network approach which was proposed in 2007 by Faity el al.[[[3]](#endnote-4)] This algorithm computes the mutual information (MI) for each pair of genes and derives a score related to the empirical distribution of the MI values. Given two random gene variables X and Y with respective expression ranges *x_i_∈R_X_*, *y_j_∈R_Y_* and probability distribution functions *p(x_i_)* and *p(y_j_)*, the MI between to genes’ expression patterns is given by

$$MI(X,Y)=\sum_{i,j} P(x_{i},y_{j})log\frac{P(x_{i},y_{j})}{P(x_{i})P(y_{j})}$$

The MI is always non-negative. It equals zero only if *X* and *Y* are statistically independent.[[[4]](#endnote-5)]The MI possesses the power of detecting the interactions that are not linear correlated. The CLR computed the statistical likelihood of MI between a TF and a potential target by comparing by comparing to background distribution of all MI scores of any two gene pairs. The most probable interactions are those whose mutual information scores stand significantly above the background distribution of mutual information scores.

## Nonnegative matrix factorization and leave-one-out cross-validation

Nonnegative matrix factorization (NMF) is an unsupervised, parts-based learning paradigm which decomposes a nonnegative matrix *V* into two nonnegative matrices *W* and *H*, *V~WH*, via multiplicative updates algorithm[[[5]](#endnote-6)]. In the context of *p×n* expression matrix *V* consisting *p* genes’ expression profiles in *n* samples, *W* is a *p×k* metagenes matrix of which each column represents a metagene, and *H* is a *k×n* expression matrix of which each row is expression pattern of a metagene in *n* samples. The rank *k* of the factorization represents the number of latent factors in the decomposition (in our case, *k* is the number of clusters). The application of NMF in biology involves in molecular pattern discovery, class comparison and prediction, and functional characterization of genes and biomedical informatics.

Leave-one-out cross-validation (LOOCV) is a kind of cross validation methods which are used for assessing how the results of a statistical analysis will generalize to an independent data set. In each run, LOOCV takes a single observation from the original sample as the validation data, and the remaining observations as the training data.

In our study, we used KMF-based consensus clustering, an unsupervised clustering method, to divide 125 ICC samples. And the robustness of clustering result was estimated by three LOOCV-based validation methods: KNNXValidation, WeightedVotingXValidation, and CARTXValidation.

1. **Reference**

   [?] Xu H, Yu H, Tu K, Shi Q, Wei C, et al. (2013) cGRNB: a web server for building combinatorial gene regulatory networks through integrated engineering of seed-matching sequence information and gene expression datasets. BMC Systems Biology 7: S7. [↑](#endnote-ref-2)
2. [?] He F, Balling R, Zeng A-P (2009) Reverse engineering and verification of gene networks: principles, assumptions, and limitations of present methods and future perspectives. Journal of biotechnology 144: 190-203. [↑](#endnote-ref-3)
3. [?] Faith JJ, Hayete B, Thaden JT, Mogno I, Wierzbowski J, et al. (2007) Large-scale mapping and validation of Escherichia coli transcriptional regulation from a compendium of expression profiles. PLoS biology 5: e8. [↑](#endnote-ref-4)
4. [?] Priness I, Maimon O, Ben-Gal I (2007) Evaluation of gene-expression clustering via mutual information distance measure. BMC bioinformatics 8: 111. [↑](#endnote-ref-5)
5. [?] Devarajan K (2008) Nonnegative matrix factorization: an analytical and interpretive tool in computational biology. PLoS computational biology 4: e1000029. [↑](#endnote-ref-6)
